# Comparison of defense responses of transgenic potato lines expressing three different *Rpi* genes to specific *Phytophthora infestans* races based on transcriptome profiling

**DOI:** 10.7717/peerj.9096

**Published:** 2020-05-05

**Authors:** Xiaohui Yang, Xiao Guo, Guangxia Chen, Daofeng Dong, Fang Liu, Yuanjun Yang, Yu Yang, Guangcun Li

**Affiliations:** 1 Institute of Vegetables and Flowers, Shandong Academy of Agricultural Sciences, Molecular Biology Key Laboratory of Shandong Facility Vegetable/National Vegetable Improvement Center Shandong Subcenter/ Huang-Huai-Hai Region Scientific Observation and Experimental Station of Tuber and Root Crop, Ministry of Agriculture and Rural Affairs, Jinan, China; 2 Institute of Vegetables and Flowers, Chinese Academy of Agricultural Sciences; Key Laboratory of Biology and Genetic Improvement of Tuber and Root Crop, Ministry of Agriculture and Rural Affairs, Beijing, China

**Keywords:** Potato, Late blight, Resistance, Mechanisms

## Abstract

Potato late blight, one of the most devastating diseases in potato, is caused by the oomycete *Phytophthora infestans*. Over 20 resistance genes have been cloned including *R1*, *R3a*, and *R3b*. The distinctions between defense response mechanisms mediated by different resistance genes are still unclear. Here we performed transcriptome profiling in three transgenic lines, *R1*, *R3a*, and *R3b,* and wild-type Desiree under inoculation with two *P. infestans* isolates, 89148 (race 0) and CN152 (super race), using RNA-seq. Compared with wild type, specific differentially expressed genes (DEGs) were identified in the three transgenic lines. The highest number of DEGs occurred in transgenic *R3b*, with 779 DEGs in response to isolate 89148 and 864 DEGs in response to infection by CN152, followed by transgenic* R1* lines with 408 DEGs for isolate 89148 and 267 DEGs for CN152. Based on gene ontology, the most common GO terms (15 for 89148 and 20 for CN152) were enriched in transgenic *R3a* and *R3b* lines. This indicates that the defense pathways mediated by *R3a* and *R3b* are more similar than those mediated by *R1*. Further separate GO analysis of up- or down-regulated DEGs showed that the down-regulated DEGs mainly functioned in mediating the resistance of potato to *P. infestans* 89148 by response to stress biological process and to CN152 by oxidation reduction biological process. KEGG pathways of DNA replication, plant-pathogen interaction and pentose and glucuronate interconversions are unique for transgenic *R1*, *R3a*, and *R3b* lines in incompatible interactions. Quantitative real-time PCR experimental validation confirmed the induced expression of DEGs in the late blight resistance signaling pathway. Our results will lay a solid foundation for further understanding the mechanisms of plant-pathogen interactions, and provide a theoretical reference for durable resistance in potato.

## Introduction

Potato is the third most common food crop in the world after wheat and rice (Birch et al., 2012). Growth and yield of potato are seriously affected by potato late blight, the most devastating plant disease caused by the oomycete *Phytophthora infestans* (Haverkort et al., 2009; Lenman et al., 2016). This pathogen caused annual losses in potato worth billions of US dollars (Haverkort et al., 2008). Researchers have searched for genetic resistance resources to control late blight, and located and cloned late blight resistance genes. So far, more than 20 resistance (*R*) genes have been cloned from different potato species including the race-specific resistance genes *RB*, *R1*, *R3a* and *R3b* (Song et al., 2003; Śliwka et al., 2013; Orbegozo et al., 2016; Vossen et al., 2016; Jiang et al., 2018). Apart from *R*-gene conferred resistance, studying potato-pathogen interactions can provide valuable insight into underlying molecular events of the defense process in potato.

The path from pathogen invasion to host defense is a complex physiological and biochemical process, involving the joint action of many genes (Dodds & Rathjen, 2010). With the rapid development of transcriptome sequencing technology (RNA-seq), comparative gene expression analysis and gene identification can be studied at the genomic level. Comparative transcriptomics of potato in response to *P. infestans* have been reported following the release of the potato DM genome sequence (PGSC, 2011). Increased expression of defense-related genes, which encode the hypersensitive induced reaction (HIR) protein and respiratory burst oxidase homolog protein B (RBOHB), were identified in transgenic *RB* (*Rpi-blb1*) strains compared to wild-type Russet Burbank. The transcription of defense-related components such as ethylene response factors and signaling receptor kinases were elevated as well (Gao et al., 2013; Gao & Bradeen, 2016).

Tetraploid potato clones Sarpo Mira and SW93-1015 exhibit strong *P. infestans* resistance (Ali et al., 2012), while Desiree (cv.) is susceptible. An early exploration of response mechanisms to *P. infestans* infection in these three potato clones was performed using gene expression microarrays and proteomics and identified several proteins specifically induced during incompatible interactions. These included a Kunitz-like protease inhibitor, several transcription factors (TFs) and an RCR3-like protein, which is involved in hypersensitive response (HR) initiation. Secreted proteins had lower abundance in the compatible interactions compared to the incompatible interactions (Ali et al., 2014). Further research was conducted using comparative transcriptomics of these three potato clones and three wild *Solanum* species in response to *P. infestans*. Gene families with enhanced expression were expanded in the resistant varieties (Sarpo Mira and SW93-1015) and species (*S. dulcamara*, *S. nigrum*) and the functions of these gene families were associated with resistance mechanisms such as HR, host programmed cell death and endopeptidase activity. Transmembrane transport and protein acylation were enriched in the susceptible group (Desiree and *S. physalifolium*) (Frades et al., 2015). Overexpression of the putative *R* genes were identified in resistant clones Sarpo Mira and SW93-1015 compared to the susceptible clone Desiree.

Desiree is a tetraploid potato cultivar with a high degree of susceptibility to *P. infestans* (Yang et al., 2018) and is a good material for genetic transformation of late blight resistance genes due to its high transformation efficiency (Armstrong et al., 2019; Ghislain et al., 2019). The transformation efficiency of Desiree with a single *R* gene construct was 3.7% for the *RB* gene, 6% for the *Rpi-blb2* gene, 7.5% for the *Rpi-vnt1.1* gene (Orbegozo et al., 2016; Roman et al., 2015; Roman et al., 2017), and up to 75% for gene stacking of these three *R* genes (Ghislain et al., 2019). Single *R* gene overexpression transgenic lines with the shared genetic background of variety Desiree (*RPi*-gene free cultivar) are ideal material for exploring the resistance and defense mechanisms mediated by *R* genes in potato. In our previous study, three different transgenic Desiree lines of *R1*, *R3a* and *R3b* were obtained via *Agrobacterium*-mediated transformation. All three transgenic lines are resistant to *P. infestans* race 0 isolate 89148 and susceptible to super-race CN152 (race 1, 3b, 4, 5, 6, 7, 8, 9, 10, 11).

So far, comparison of disease response mechanisms mediated by different vertical resistance genes has not been reported in the potato-*P. infestans* system. *R1*, *R3a* and *R3b,* the race-specific resistance genes, were derived from the wild species *S. demissum* (Ballvora et al., 2002; Huang et al., 2005; Li et al., 2011) and specifically recognize the avirulence genes *Avr1*, *Avr3a* and *Avr3b*, respectively. *R1* is located on chromosome 5 and belongs to the leucine zipper-nucleotide binding site-leucine rich repeat (LZ-NBS-LRR) type. *R3a* and *R3b* are both located on chromosome 11 and are closely linked in a major late blight resistance complex (MLB) of potato with a genetic distance of 0.4 cM. Both genes belong to the coiled coil (CC)-NBS-LRR type, with distinct resistance specificities to *P. infestans* (Huang et al., 2004; Huang et al., 2005). *R1* was significantly different from *R3a* and *R3b*, with low nucleotide consistency (39.1% and 39.2%, respectively), while the nucleotide identity of *R3a* and *R3b* is 82% and the identity of the amino acid sequence is 73%. Further analysis of the LRR domain of *R3a* and *R3b* showed that the *R3a* gene encoded 29 LRRs, while the *R3b* gene encoded 28 LRRs, and each encoded two unique LRRs (Li et al., 2011), which may define recognition specificity. The differences and similarities of defense responses in potato-*P. infestans* interactions mediated by *R1*, *R3a*, and *R3b* remain unknown.

In this study, transcriptome profiling by RNA-seq was performed using the wild-type Desiree and three transgenic lines to explore the role of these *R* genes in response to infection with *P. infestans*. This study compared the differences of genetic responses and gene expression in response to *P. infestans* infection, analyzed the key genes in the metabolic pathway of disease resistance, explored the mechanisms of disease resistance, and contrasted the disease resistance metabolic networks across the *R1*, *R3a,* and *R3b* lines. These results will lay a solid foundation for further understanding the mechanisms of plant-pathogen interactions, and provide a theoretical reference for durable resistance in potato.

## Materials and Methods

### Plant materials

Tetraploid potato cultivar Desiree and its three transgenic lines with the genes *R1*, *R3a* and *R3b* were used. Sterile culture of *in vitro* plants was carried out in culture bottles (10 cm height × 6.5 cm diameter) containing MS medium by single segmental regeneration and asexual propagation. All samples were cultured for 4 weeks under 24 °C 16 h light/8 h dark conditions with 10 seedlings per bottle.

### Inoculation with *P. infestans* isolates

*P. infestans* isolates 89148-9 (race 0) and CN152 (race 1, 3b, 4, 5, 6, 7, 8, 9, 10, 11) (Li et al., 2017) were used in this study. Sporangium formation was induced by activation of *P. infestans* on oat agar medium, and cultured in the dark at 18 °C for 7–10 days. Pre-chilled sterile water was added to the culture dish and placed at 4 °C for 3 h to release zoospores. The spore concentration was counted using a Fuchs-Rosenthal counting chamber. The final spore concentration was adjusted to 5 × 10^4^ spores/mL. At least one week before infection, four-week-old plants were transferred to an infection chamber with 100% humidity and a 10:14 light:dark cycle. Plants were sprayed with an encysted zoospore suspension from *P. infestans* isolates 89148 and CN152 until the leaf surfaces were fully saturated with the zoospore suspension.

### RNA extraction and sequencing

Sampling was carried out at 24-hours after inoculation of *P. infestans*, and 10 seedlings of each treatment were collected at the same time. The roots were removed and all aboveground tissues of 10 seedlings were mixed as one sample. A total of 8 samples were collected. The samples were frozen with liquid nitrogen and stored at −80 °C for subsequent RNA extraction and sequencing. Total RNA of samples was extracted using TRIzol reagent (Invitrogen, Carlsbad, CA, USA), digested by TURBO DNase I (Ambion, Austin, TX, USA), precipitated and dissolved by ethanol, and detected using *β*-actin as a reference gene. RNA concentration and OD260/OD280 were observed using an ultraviolet spectrophotometer and 1% agarose gel electrophoresis. Non-degraded RNAs with OD260/OD280 ∼2.0 were used to generate the cDNA sequencing library with the NEBNext Ultra™ RNA Library Preparation Kit (NEB, USA) according to the manufacturer’s recommendations. The RNA quality was checked using an Agilent BioAnalyzer 2100 system and cDNA libraries were sequenced on the Illumina HiSeq 2500 platform.

### Raw data quality assessment

Raw data were processed by removing reads containing adapter, reads containing poly-N and low-quality reads, and then the clean data (clean reads) were retained. At the same time, Q20, Q30, GC-content and sequence duplication level of the clean data were calculated. All the downstream analyses were based on high-quality clean data.

### Comparison of reference genomes and functional annotations

Clean reads were then mapped to the potato reference genome sequence. The reference accession, the doubled haploid *S. tuberosum* Group *Phureja* clone DM1-3 516R44 (hereafter referred to as DM) genome sequence (SolTub 3.0) and annotation files were downloaded from the ENSEMBL plants database (http://plants.ensembl.org/Solanum_tuberosum/Info/Index) (Bolser et al., 2017). Only reads with a perfect match or one mismatch were further analyzed and annotated based on the reference genome. TopHat2 tools soft were used to align RNA-seq reads against the reference genome (Kim et al., 2013).

### Identification of differentially expressed genes

Gene expression levels were estimated by fragments per kilobase of transcript per million fragments mapped (FPKM) and normalized using HTseq-count and EBSeq (Florea, Song & Salzberg, 2013; Leng et al., 2013; Anders, Pyl & Huber, 2015). Differential expression analysis was performed using EBSeq based on the negative binomial distribution (Anders & Huber, 2010). Normalized FPKM of genes expressed in the transgenic *R1*, *R3a,* and *R3b* plants after inoculation was compared with FPKM in the wild-type Desiree, and the DEGs were identified. Also, the DEGs involved in incompatible/compatible reactions were analyzed by comparing transcriptomic data in each of the transgenic lines infected by the two tested *P. infestans* strains with different virulences. The resulting *p*-values were adjusted using the Benjamini–Hochberg approach for controlling the false discovery rate (*FDR*). Genes with normalized expression fold-change greater than 2, and *FDR* < 0.05 were assigned as differentially expressed. The DEGs were annotated based on the functional annotation information of ENSEMBL plants release *S. tuberosum* SolTub_3.0 genes.

DEGs identified in 89148 treated samples were represented by 8_TR1, 8_TR3a and 8_TR3b, and DEGs identified in CN152 treatments samples were represented by CN_TR1, CN_TR3a and CN_TR3b.

### GO and KEGG enrichment

Using the potato SolTub_3.0 database as a reference, the gene ontology GO (Gene Ontology) enrichment analysis of DEGs was carried out by agriGO (http://bioinfo.cau.edu.cn/agriGO/analysis.php). KOBAS software (http://kobas.cbi.pku.edu.cn/index.php) was used to analyze the significant enrichment of DEGs in KEGG pathways (Mao et al., 2005). The significance of enrichment of each GO and KEGG term was assessed by *p*-value <0.05 or *FDR* < 0.05.

### Validation of DEGs by qRT-PCR

Sixteen DEGs were randomly selected for quantitative real-time PCR (qRT-PCR) to verify the RNA-seq results. Specific primers were designed for qRT-PCR analysis using Primer 5 and listed in Table S1. The RNA samples for qRT-PCR were prepared by the same methods with RNA-seq and reverse-transcribed to cDNAs using PrimeScript™ RT reagent kit with gDNA Eraser (TaKaRa). qRT-PCR was performed using SYBR® Green PCR master mix reagents with 10 µL SYBR Premix *Ex Taq* (TaKaRa, Japan), 0.5 µL of both forward and reverse primers, 7 µL of double-distilled water and 2 µL (40 ng/µL) cDNA in one reaction system. Then qRT-RCR reactions were performed on the Bio-Rad CFX96 C1000™ instrument with the cycle steps of pre-denaturation at 95 °C, 30 s, 1 cycle; 95 °C, 5 s, 60 °C, 30 s, 40 cycles. Gene expression was analyzed by CFX-manager software (CFX96 Real-Time System; Bio-Rad). The relative expression level of each gene was calculated by the 2^−ΔΔ^^Ct^ method (Livak & Schmittgen, 2001) using the *GAPDH* gene as an internal reference. All assays were performed in triplicate under identical conditions and correlations between qRT-PCR and RNA-seq data were evaluated using Pearson correlation coefficients.

### Clustering of late blight resistance genes

A total of 20 cloned resistance (*R*) genes to potato late blight, including *R1*, *R3a* and *R3b* were used in this study. The CDS of these genes were downloaded from the website of the National Center for Biotechnology Information (https://www.ncbi.nlm.nih.gov/). The phylogenetic tree of these 20 *R* genes was constructed using the neighbor-joining (NJ) method in MEGA 5 (Tamura et al., 2011).

## Results

### Transcriptome sequencing and mapping

**Figure 1:**
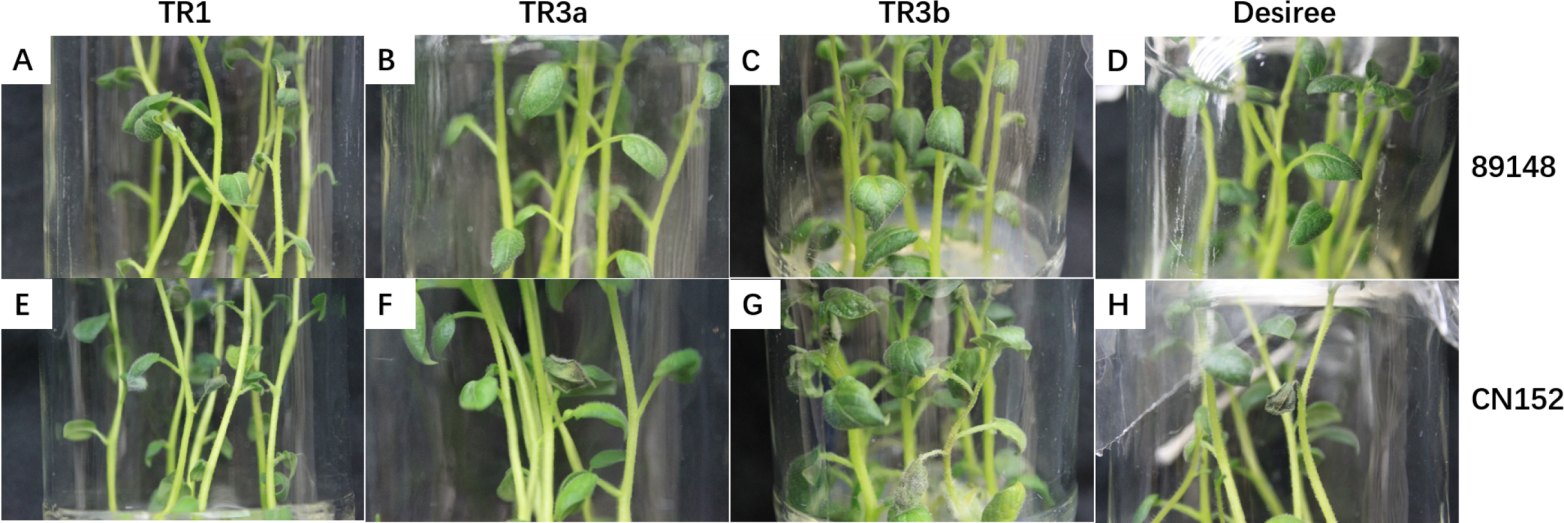
Phenotypes of transgenic *R1*, *R3a*, and *R3b* lines and wild-type Desiree under infection with *P. infestans* isolates 89148 (race 0) and super race CN152 (race 1, 3b, 4, 5, 6, 7, 8, 9, 10, 11). (A–C) Transgenic *R1*, *R3a*, and *R3b* lines under 89148. (D) Desiree under 89148. (E–G) Transgenic *R1*, *R3a*, and *R3b* lines under CN152. (H) Desiree under CN152.

The transgenic *R1*, *R3a*, *R3b* lines and wild-type Desiree were infected with two *P. infestans* isolates, 89148 (universal race 0) and CN152 (super-race 1, 3b, 4, 5, 6, 7, 8, 9, 10, 11). At 24 h post inoculation (hpi), all three transgenic lines showed resistance to 89148 (Figs. 1A, 1B, and 1C), however, all three transgenic lines expressed susceptibility to CN152 (Figs. 1E, 1F, and 1G). Wild-type Desiree was susceptible to both *P. infestans* isolates while did not show obvious susceptible symptom until 72 hpi under 89148 infection (Figs. 1D and 1H, Yang et al., 2018).

To unravel the role of different *R* genes in the early molecular response to *P. infestans* both in incompatible and compatible interactions, the present study included transcriptional profiling of wild-type (WT) and the transgenic plants *R1*, *R3a,* and *R3b* (hereafter referred as TR1, TR3a, and TR3b) 24 h after infection of 89148 and CN152 by RNA-seq. In total, eight RNA libraries derived from seedlings sampled at 24 hpi were sequenced using the Illumina HiSeq 2500 system with the 125-cycle paired-end sequencing protocol.

After data quality assessment, a total of 184,632,455 clean paired-end reads (46.52 Gb) were generated with an average of 23,079,057 paired end reads (5.81 Gb) for each sample. Over 92.99% of these reads were ≥Q30 and the average GC content was 43.85% (Table 1). The clean data were then aligned to the potato DM reference genome, and 74.43%–76.52% of the reads per sample mapped to the reference genome, while 72.28%–74.51% of the reads per sample uniquely aligned to the reference genome (Table 1).

### Differentially expressed gene analysis assessment

DEGs were identified by comparing the transgenic materials to wild-type Desiree (Table 2). Under infection by the isolate 89148, the number of DEGs in transgenic *R3b* line was the highest (1763), followed by *R1* (1318), and the number of DEGs in transgenic *R3a* was the lowest (1171). More down-regulated DEGs (728, 627) were detected than up-regulated DEGs (590, 544) in TR1 and TR3a. Almost the same numbers of up- and down-regulated DEGs were present in TR3b. The same trend was observed under CN152 inoculation with the highest number of DEGs in TR3b (1857) and the lowest number of DEGs in TR3a (1096), including 408, 456, and 929 up-regulated DEGs and 718, 640, and 928 down-regulated DEGs in the TR1, TR3a, and TR3b lines, respectively.

**Table 1:** Basic summary of RNA-sequencing results.

**Sample**	**Clean reads**	**Clean base (Gb)**	**GC (%)**	**≥Q30%**	**Mapped reads**	**Unique mapped reads**
8-TR1	20,966,078	5.28	43.57	93.25	31,593,465 (75.34%)	30,524,047 (72.79%)
8-TR3a	22,768,486	5.74	43.99	93.39	34,767,380 (76.35%)	33,643,575 (73.88%)
8-TR3b	22,374,538	5.64	44.39	93.01	33,820,658 (75.58%)	32,494,685 (72.62%)
8-WT	21,464,990	5.41	43.97	93.32	32,091,522 (74.75%)	31,083,726 (72.41%)
CN-TR1	23,841,015	6.01	44.26	93.34	36,212,550(75.95%)	34,651,869 (72.67%)
CN-TR3a	27,169,034	6.85	43.51	93.46	41,579,472 (76.52%)	40,488,609 (74.51%)
CN-TR3b	25,171,405	6.34	43.62	92.99	37,875,481(75.24%)	36,390,197 (72.28%)
CN-WT	20,876,909	5.26	43.51	93.38	31,077,130 (74.43%)	30,251,599 (72.45%)

**Notes.**

WT potato cultivar Desiree 8 *Phytophthora infestans* isolate 89148 (race 0) CN *P. infestans* isolate CN152 (race 1, 3b, 4, 5, 6, 7, 8, 9, 10, 11)

**Table 2:** Differentially expressed genes that were up-regulated or down-regulated under inoculation by *P. infestans* isolates.

**DEG Sets**	**All DEGs**	**Up-regulated DEGs**	**Down-regulated DEGs**
8_TR1	1318	590	728
8_TR3a	1171	544	627
8_TR3b	1763	880	883
CN_TR1	1126	408	718
CN_TR3a	1096	456	640
CN_TR3b	1857	929	928

**Notes.**

8 Phytophthora infestans isolate 89148 (race 0) CN *P. infestans* isolate CN152 (race 1, 3b, 4, 5, 6, 7, 8, 9, 10, 11)

**Figure 2:**
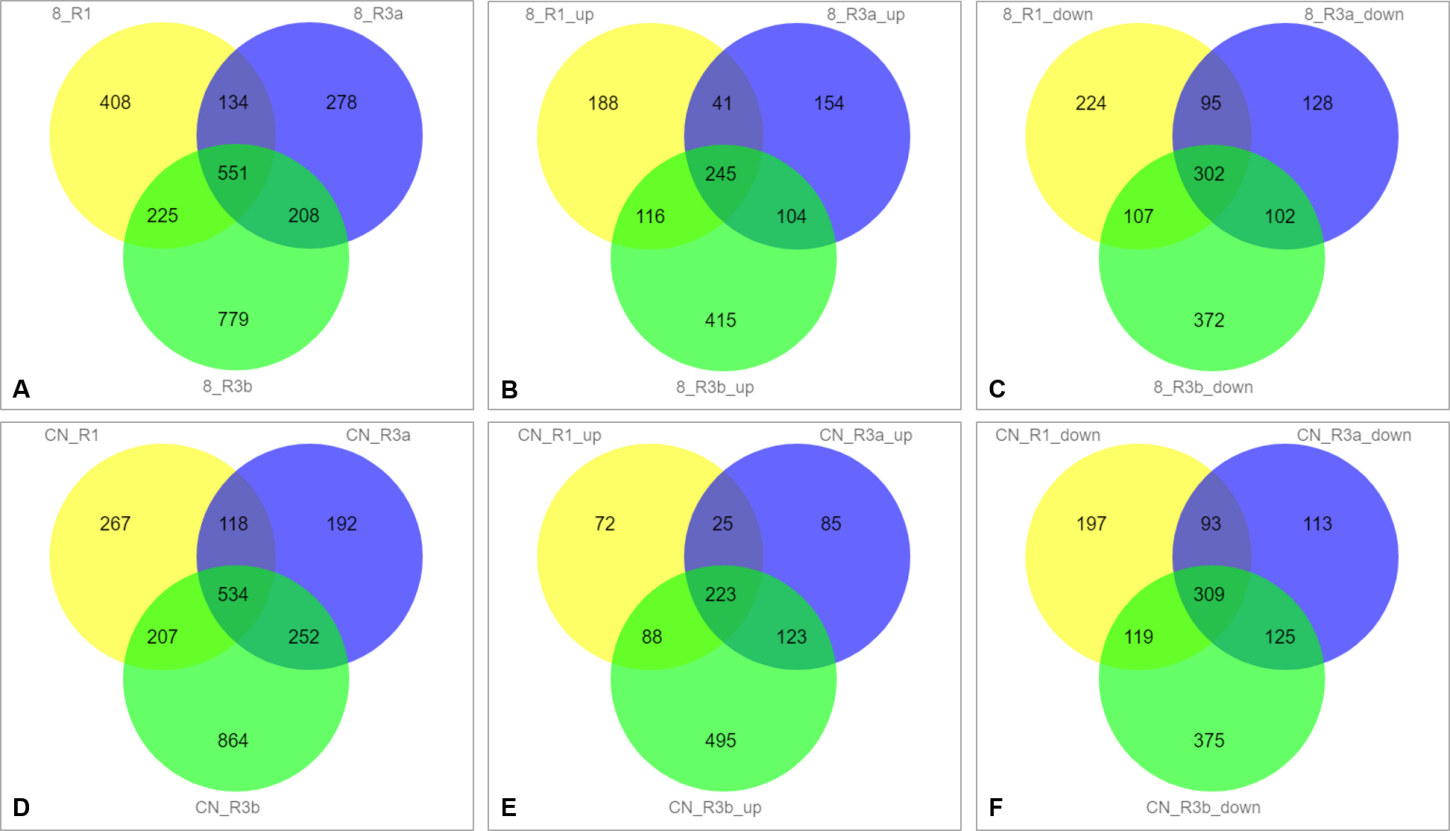
Venn diagram analysis of differently expressed genes (DEGs) in different transgenic *R1*, *R3a* and *R3b* lines under infection with *P.infestans* isolates 89148 and CN152. (A) Total DEGs of transgenic *R1*, *R3a* and *R3b* lines under 89148. (B) The up-regulated DEGs in transgenic *R1*, *R3a* and *R3b* lines under 89148. (C) The down-regulated DEGs in transgenic *R1*, *R3a* and *R3b* lines under 89148. (D) Total DEGs of transgenic *R1*, *R3a* and *R3b* lines under CN152. (E) The up-regulated DEGs in transgenic *R1*, *R3a* and *R3b* lines under CN152. (F) The down-regulated DEGs in transgenic *R1*, *R3a* and *R3b* lines under CN152. 8 = 89148, CN = CN152.

The Venn diagram showed 408, 278, and 779 unique DEGs in TR1, TR3a, and TR3b, respectively, when inoculated with 89148 (Fig. 2A), and 267, 192, and 864 DEGs when inoculated with CN152, respectively (Fig. 2D). The most unique DEGs were identified in transgenic *R3b* lines, followed by transgenic *R1* and *R3a* lines. Under infection with the same pathogen, more DEGs were involved in the resistance to pathogen infection in transgenic *R3b* lines, while transgenic *R3a* lines can defend against infection by mobilizing fewer genes. The tendency of up- and down-regulated DEGs is the same as that of total DEGs in transgenic lines (Figs. 2B, 2C, 2E and 2F). This indicates the weakest resistance in *R3b* and the strongest resistance in *R3a*, though both genes exist within the same *R* gene cluster.

When inoculated with 89148, TR1 and TR3a shared the fewest DEGs, with 134 genes overlapping. Approximately the same number of DEGs were shared by TR1 and TR3b, TR3a and TR3b with 225 and 208, respectively (Fig. 2A). The same trend was evident under CN152 inoculation. The common DEGs shared between TR1 and TR3a, TR1 and TR3b, TR3a and TR3b, were 118, 207, and 252, respectively (Fig. 2D). These results suggest that *R1* and *R3a* have more similar resistance levels than *R1* vs. *R3b*, and *R3a* vs. *R3b*.

### Enriched KEGG pathways in DEGs

**Figure 3:**
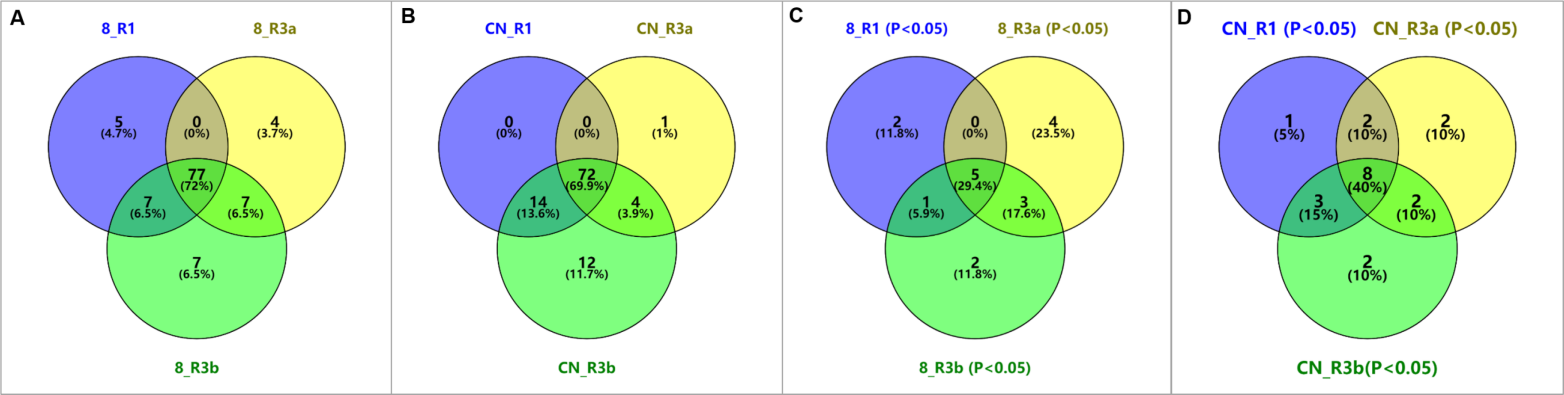
Venn diagram of KEGG pathways of differentially expressed genes (DEGs) under *P. infestans* isolates 89148 and CN152 inoculation in different transgenic *R1*, *R3a*, and *R3b* lines, respectively. (A) Total pathways of three transgenic lines under 89148. (B) Total pathways of three transgenic lines under CN152. (C) Significant pathways of three transgenic lines with *p*-value <0.05 under 89148. (D) Significant pathways of three transgenic lines with *p*-value <0.05 under CN152. 8 = 89148, CN = CN152.

KEGG metabolic pathways were analyzed across all DEGs. Under the *P. infestans* 89148 treatments, the DEGs derived from *R1*, *R3a*, and *R3b* lines were mapped to 89, 88, and 98 canonical reference pathways, respectively (Fig. 3A). Under CN152 treatments, DEGs were enriched in 86, 77 and 102 canonical reference pathways, respectively (Fig. 3B). More differentially expressed pathways were identified in TR3b than in TR3a and TR3b. As TR3b is the most susceptible genotype, more pathways may be involved to achieve the purpose of disease defense. Eight, 12 and 11 reference canonical pathways were significant (*p*<0.05) for 89148 isolate (Fig. 3C). For infection by CN152, 14, 14 and 15 reference canonical pathways were significant with *p*-value <0.05 (Fig. 3D).

**Figure 4:**
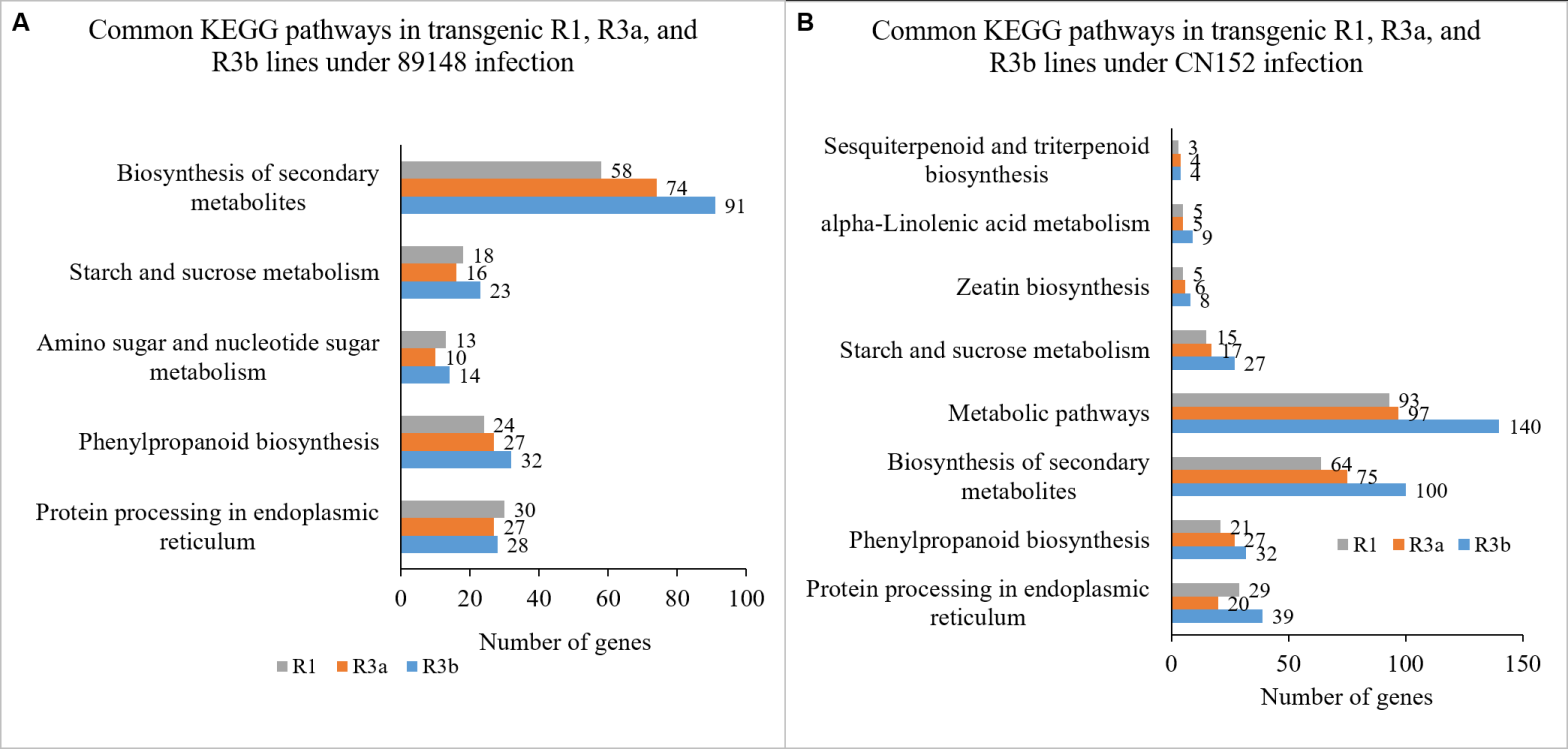
KEGG pathways of differentially expressed genes (DEGs) annotated in the Kyoto Encyclopedia of Genes and Genomes (KEGG) data library under *P. infestans* isolates 89148 and CN152 infection. (A) Common pathways in transgenic *R1*, *R3a*, and *R3b* lines under 89148. (B) Common pathways in transgenic *R1*, *R3a*, and *R3b* lines under CN152. The *x*-axis indicates the number of DEGs annotated in the pathway; the *y*-axis indicates the name of significant pathways in KEGG.

When inoculated with 89148, five common pathways in KEGG were significantly enriched (*p* <0.05) in the three transgenic lines (Fig. 3C). These include protein processing in the endoplasmic reticulum (sot04141), phenylpropanoid biosynthesis (sot00940), amino sugar and nucleotide sugar metabolism (sot00520), starch and sucrose metabolism (sot00500), as well as biosynthesis of secondary metabolites (sot01110). The biosynthesis of secondary metabolites has the most DEGs with 58, 74, and 91 genes enriched for TR1, TR3a, and TR3b (Fig. 4A). Under inoculation with CN152, eight common pathways in KEGG were significantly enriched (*p* < 0.05) in the three transgenic lines (Fig. 3D), including protein processing in the endoplasmic reticulum (sot04141), phenylpropanoid biosynthesis (sot00940), biosynthesis of secondary metabolites (sot01110), metabolic pathways (sot01100), starch and sucrose metabolism (sot00500), zeatin biosynthesis (sot00908), alpha-linolenic acid metabolism (sot00592), and sesquiterpenoid and triterpenoid biosynthesis (sot00909) (Fig. 4B). Metabolic pathways (sot01100) had the most abundant DEGs, followed by biosynthesis of secondary metabolites (64, 75, 100). Amino sugar and nucleotide sugar metabolism (sot00520) is the specific pathway involved in the incompatible action by 89148 with 13, 10, and 14 DEGs for TR1, TR3a, and TR3b (Table S2); while metabolic pathways (sot01100), zeatin biosynthesis (sot00908), alpha-linolenic acid metabolism (sot00592) and sesquiterpenoid and triterpenoid biosynthesis (sot00909) are the specific pathways involved in the compatible action by CN152.

### Specific enriched pathways in different transgenic *R1*, *R3a*, and *R3b* lines

Venn diagram results showed there were two, four, and two significant (*p* < 0.05) KEGG pathways specific to TR1, TR3a and TR3b, respectively, under inoculation with 89148 (Fig. 3C), and one, two, and two pathways, respectively, specific under CN152 infection (Fig. 3D). The top enriched pathway is unique to each transgenic line.

Under 89148 infection, DNA replication (sot03030), plant-pathogen interaction (sot04626), and pentose and glucuronate interconversions (sot00040) are the top significantly enriched pathways in TR1, TR3a, and TR3b, respectively (Table 3). In DNA replication pathway, nine genes were enriched in TR1 lines, which contained four minichromosome maintenance (MCM) protein genes *MCM4*, *MCM5*, *MCM6*, and *MCM7*. These MCM coding genes were all down-regulated. The other two genes of unknown function (PGSC0003DMG400011569 and PGSC0003DMG402011204) had highly increased expression of up to 25-fold.

**Table 3:** The KEGG pathways specific for transgenic *R1*, *R3a*, and *R3b* lines.

Isolate	KEGG Term	KEGG ID	DEGs number	*P*-value	*R* genes
89148	DNA replication	sot03030	9	0.0009	*R1*
	Butanoate metabolism	sot00650	4	0.0217	*R1*
	Plant-pathogen interaction	sot04626	16	0.00244	*R3a*
	Photosynthesis	sot00195	7	0.0259	*R3a*
	alpha-Linolenic acid metabolism	sot00592	5	0.0348	*R3a*
	Nitrogen metabolism	sot00910	4	0.0395	*R3a*
	Pentose and glucuronate interconversions	sot00040	14	0.0041	*R3b*
	Sesquiterpenoid and triterpenoid biosynthesis	sot00909	4	0.0264	*R3b*
CN152	Cutin, suberine and wax biosynthesis	sot00073	5	0.0152	*R1*
	Diterpenoid biosynthesis	sot00904	5	0.0129	*R3a*
	Amino sugar and nucleotide sugar metabolism	sot00520	9	0.0418	*R3a*
	Pentose and glucuronate interconversions	sot00040	14	0.0051	*R3b*
	DNA replication	sot03030	8	0.0191	*R3b*

In the pathway of plant-pathogen interaction, a total of 16 genes were differentially expressed in TR3a lines, which encoded pathogenesis-related protein 1 (PR-1, two members), heat shock protein (HSP, three members), respiratory burst oxidase homolog protein (RBOH, two members), receptor protein kinase (two members), calcium-dependent protein kinase (CDPK, two members), and CC-NBS-LRR resistance protein (one member). One PR-1 gene (PGSC0003DMG400005109) had the highest fold change of 27.6. In the pathway of pentose and glucuronate interconversions for TR3b lines, a total of 14 DEGs were enriched and encoded pectinesterase (five members) and pectase lyase (nine members). Except for one pectinesterase coding gene (PGSC0003DMG400000293) that was up-regulated, the other genes were down-regulated in TR3b lines (Table S3). This indicated that most down-regulated DEGs played were essential in the response to 89148 infection.

Under CN152 infection, only the pathway of cutin, suberine, and wax biosynthesis (sot000735) was significant for TR1 lines including five DEGs that encoded feruloyl transferase (PGSC0003DMG400031731), Acyl CoA reductase (PGSC0003DMG400007405), CYP86A33 fatty acid omega-hydroxylase (PGSC0003DMG400002111), Fatty acyl-CoA reductase 3 (PGSC0003DMG400007113) and Fatty acyl-CoA reductase 2 (PGSC0003DMG400008885). For diterpenoid biosynthesis (sot00904) in TR3a lines, one copalyl diphosphate synthase gene (PGSC0003DMG400001948), one GA 3-oxidase gene (PGSC0003DMG400005698), and three gibberellin (GA) 2-oxidase genes (PGSC0003DMG400027632, PGSC0003DMG400021095 and PGSC0003DMG400027631) were enriched. For TR3b lines, the top pathway under CN152 was pentose and glucuronate interconversions (sot00040), which was the same as that under 89148. All 14 DEGs were down-regulated with five genes encoding pectinesterase, eight encoding pectase lyase and one encoding polygalacturonase 7 (PGSC0003DMG400020372) (Table S4). This indicated most down-regulated DEGs functioned directly in response to CN152 infection.

**Figure 5:**
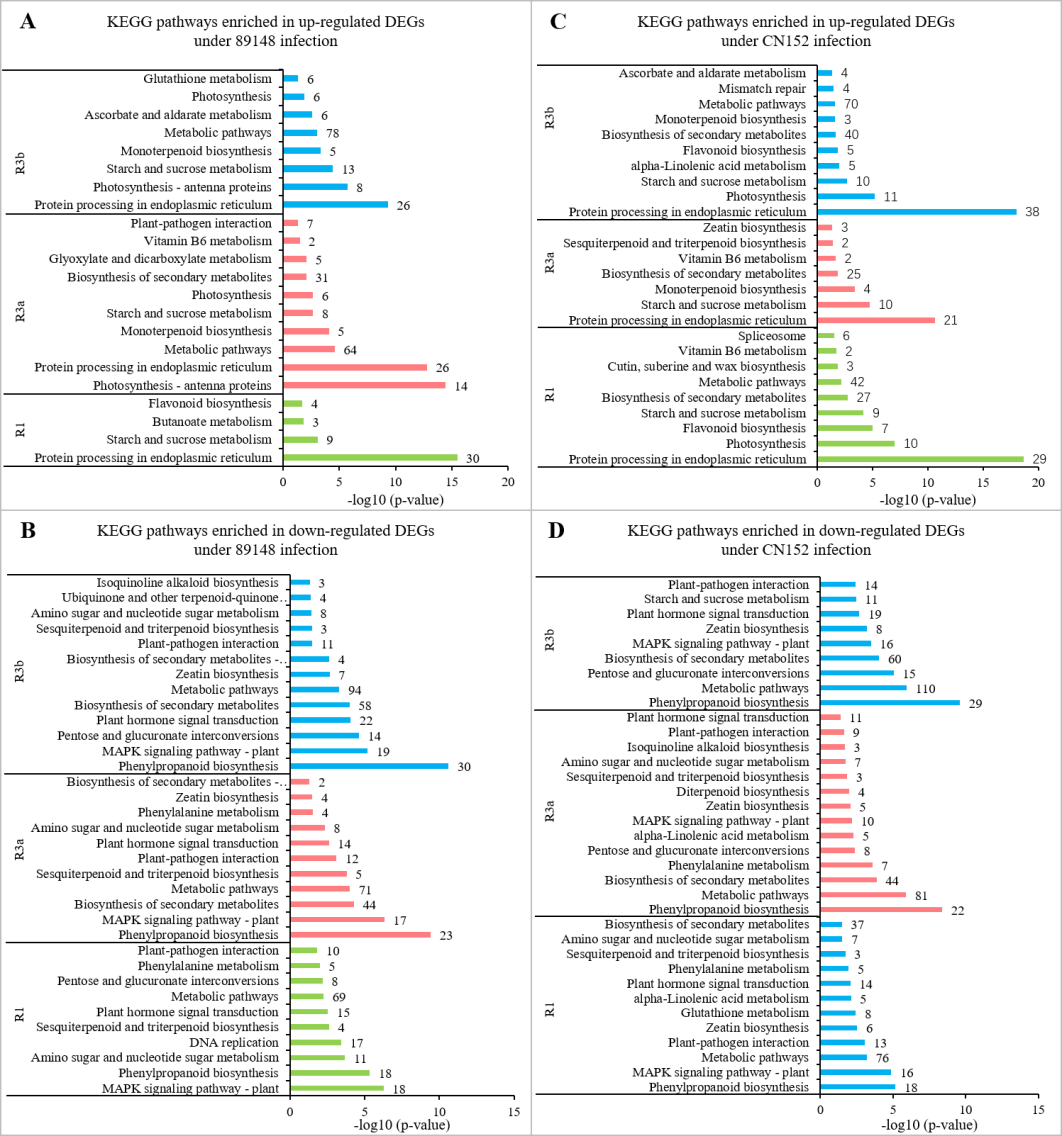
KEGG metabolic pathways enriched in up- and down-regulated genes in transgenic *R1*, *R3a*, and *R3b* lines with inoculation by *P. infestans* isolates 89148 and CN152. (A) Pathways enriched in up-regulated genes under 89148. (B) Pathways enriched in down-regulated genes under 89148. (C) Pathways enriched in up-regulated genes under CN152. (D) Pathways enriched in down-regulated genes under CN152.

To further investigate the regulation mechanism of resistance-related genes, the up- or down-regulated DEGs separately in the KEGG enrichment were separately analyzed. For TR1, TR3a, and TR3b by 89148 infection, the up-regulated DEGs were significantly enriched in four, ten and eight KEGG metabolic pathways, respectively (Fig. 5A). The top pathways in each were protein processing in the endoplasmic reticulum (sot04141), photosynthesis-antenna proteins (sot00196) and protein processing in the endoplasmic reticulum (sot04141), respectively. The down-regulated DEGs of TR1, TR3a, and TR3b were significantly enriched in ten, eleven, and 13 KEGG metabolic pathways, respectively (Fig. 5B). The top two pathways for all three were phenylpropanoid biosynthesis (sot00940) and MAPK signaling pathway-plant (sot04016). The pathway of plant-pathogen interaction (sot04626) was enriched in the down-regulated DEGs of TR1 (10 members), TR3a (12 members), and TR3b (11 members) (Table S5). This indicated that the down-regulation of DEGs was essential in the incompatible interaction between the transgenic plants and *P. infestans* isolate 89148.

For TR1, TR3a, and TR3b with CN152 infection, the up-regulated DEGs were significantly enriched in nine, seven, and ten KEGG metabolic pathways, respectively (Fig. 5C). The top pathway was protein processing in the endoplasmic reticulum (sot04141) with 29, 21, and 38 DEGs, respectively. The down-regulated DEGs of TR1, TR3a, and TR3b were significantly enriched in 12, 14, and 17 KEGG metabolic pathways, respectively (Fig. 5D). The top pathways were phenylpropanoid biosynthesis (sot00940), metabolic pathways (sot01100), and MAPK signaling pathway-plant (sot04016). The pathway of plant-pathogen interaction (sot04626) was enriched in the down-regulated DEGs of TR1 (13), TR3a (9), and TR3b (14) (Table S5). This indicated that the down-regulation of DEGs are required in the compatible interaction between the transgenic plants and *P. infestans* isolate CN152.

### Gene ontology classification of DEGs

To get an overview of the functional category of the genes that participated in the *P. infestans* infection response, the DEGs were subjected to Gene Ontology (GO) enrichment analysis. Under infection by 89148, 57.4% of the total DEGs in transgenic *R1* lines (TR1) were enriched for different GO terms, including 53 significantly enriched terms (*FDR* < 0.05). From DEGs in TR3a and TR3b, 60.6% and 57.1% of the total genes were enriched for different GO terms, and 69 and 68 terms were significantly enriched (*FDR* < 0.05) (Table 4). After inoculation with CN152, among the DEGs generated from transgenic *R1*, *R3a*, and *R3b* lines, 59.8%, 60.0% and 57.0% of the genes were annotated with GO terms. Of these GO terms, 55, 62, and 74 were significant at *FDR* < 0.05 (Table 4).

**Table 4:** GO terms enriched in all differentially expressed genes.

**DEG Sets**	**DEGs number**	**Enriched DEGsin GO**	**Significant GO terms (*FDR* < 0.05)**
8_TR1	1318	757	53
8_TR3a	1171	710	69
8_TR3b	1763	1007	68
CN_TR1	1126	673	55
CN_TR3a	1096	658	62
CN_TR3b	1857	1058	74

**Figure 6:**
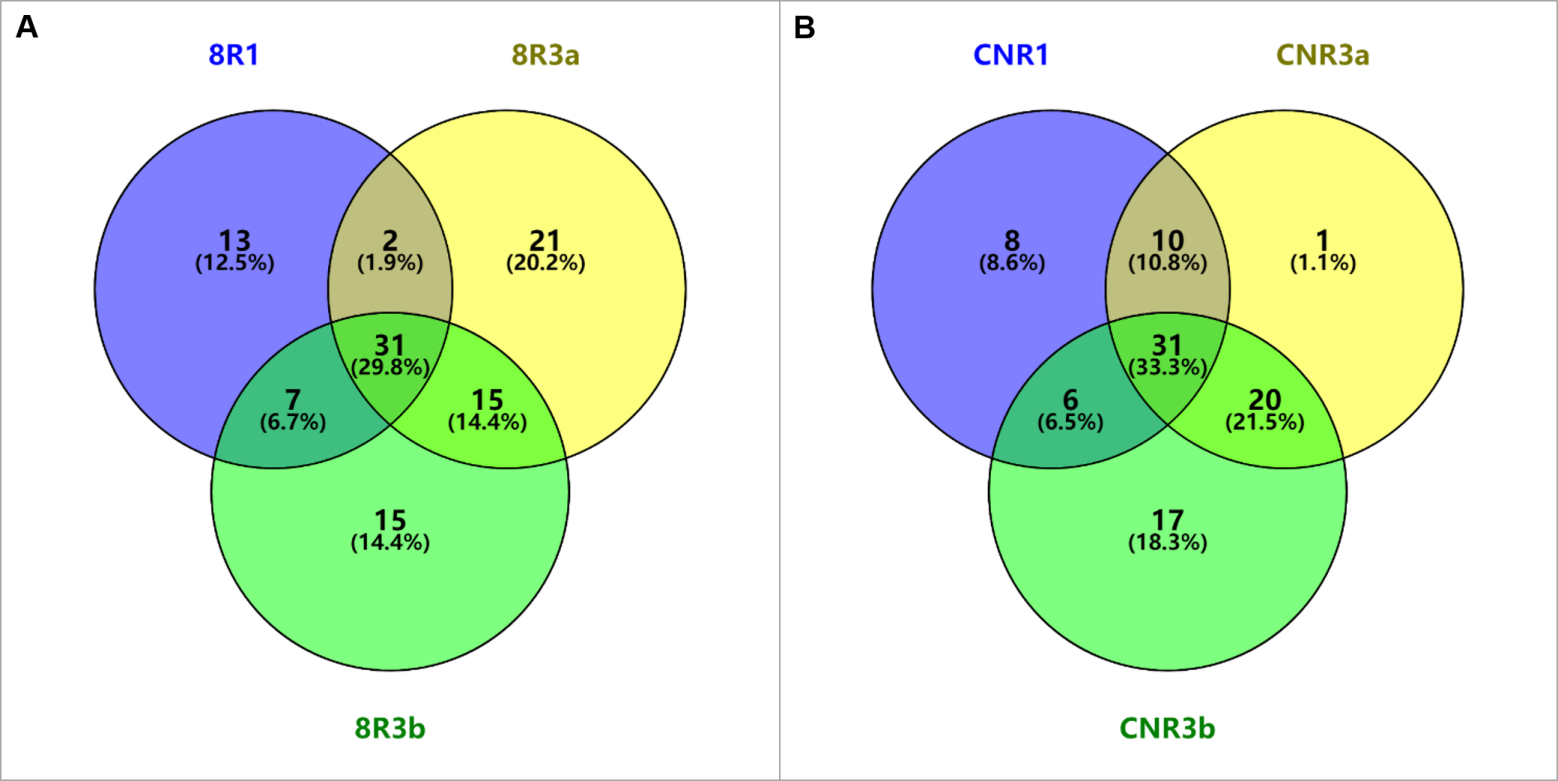
The comparison of GO terms enriched in different transgenic lines of *R1*, *R3a*, and *R3b* inoculated with *P. infestans* isolates 89148 and CN152. (A) Enriched GO terms under 89148. (B) Enriched GO terms under CN152. 8 = 89148, CN = CN152.

A Venn diagram analysis showed that there were 13, 21, and 15 GO terms specific to TR1, TR3a, and TR3b, respectively, under inoculation with 89148, and eight, one and 17 terms were specific under CN152 infection. In both inoculation conditions, 31 common GO terms were shared across TR1, TR3a, and TR3b (Figs. 6A and 6B). TR3a and TR3b shared the largest number of enriched GO terms (15 for 89148 and 20 for CN152) (Figs. 6A and 6B). TR1 and TR3a shared the smallest number of GO terms under 89148 infection (two common terms), and under CN152 infection the smallest number of GO terms were shared across TR1 and TR3b (six common terms) (Figs. 6A and 6B). This indicated that the defense pathways mediated by *R3a* and *R3b* are more similar than those shared between *R1* and *R3a,* or *R1* and *R3b* in both incompatible and compatible interactions of potato and *P. infestans*.

**Figure 7:**
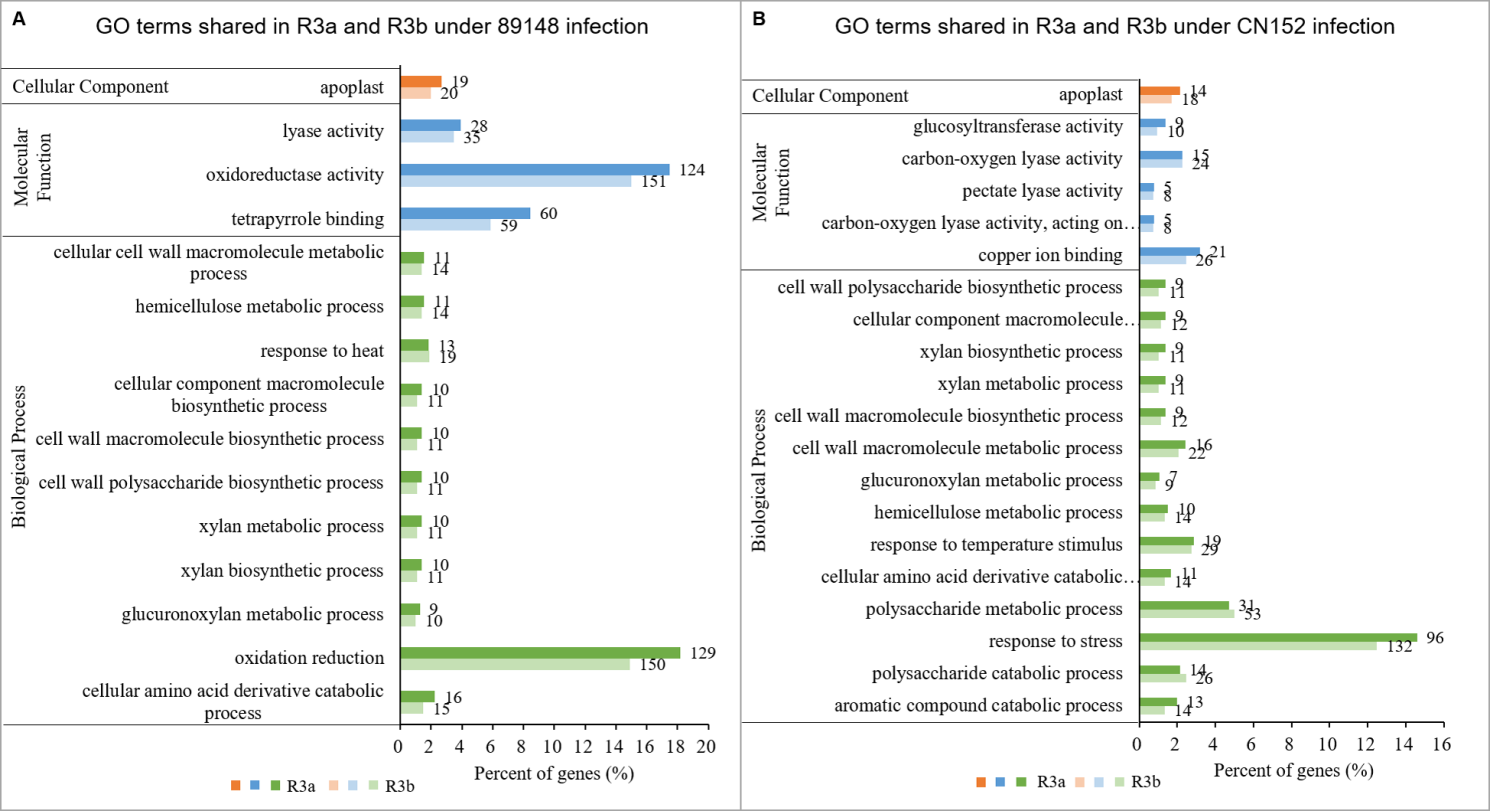
The shared GO terms between transgenic lines of *R3a* and *R3b* inoculated with *P. infestans* isolates 89148 and CN152. (A) GO terms shared in transgenic lines of *R3a* and *R3b* under 89148. (B) GO terms shared in transgenic lines of *R3a* and *R3b* under CN152. *Y*-axis, the three functional categories of GO terms: biological processes, molecular functions, and cellular components. *X*-axis, the percentages of DEGs for each GO term. Numbers to the right of each bar represent the number of DEGs for each GO term.

GO enrichment analysis is broken down into three categories: biological process (BP), molecular function (MF), and cellular component (CC). Among the 15 common GO terms shared between TR3a and TR3b under 89148 infection (Fig. 7A), a total of 11 terms were enriched in the BP category, including cellular amino acid derivative catabolic process (GO:0042219), oxidation reduction (GO:0055114), and glucuronoxylan metabolic process (GO:0010413). The biological process of oxidation reduction (GO:0055114) had the most DEGs, at 129 in TR3a and 150 in TR3b (Table S6). The terms of tetrapyrrole binding (GO:0046906), oxidoreductase activity (GO:0016491), and lyase activity (GO:0016829) were under the MF category. Oxidoreductase activity (GO:0016491) had the most DEGs, at 124 in TR3a and 151 in TR3b.

Among the 20 GO terms shared by transgenic *R3a* and *R3b* lines inoculated with CN152, 16 terms belonged to the BP category, three were under MF, and one was enriched in the CC category (Fig. 7B). For the BP category, aromatic compound catabolic process (GO:0019439), polysaccharide catabolic process (GO:0000272), and response to stress (GO:0006950) were among the enriched terms. Response to stress (GO:0006950) had the most DEGs at 96 in TR3a and 132 in TR3b (Table S7). The shared terms enriched in MF are copper ion binding (GO:0005507), carbon-oxygen lyase activity, acting on polysaccharides (GO:0016837), and pectate lyase activity (GO:0030570).

### Specific enriched terms in different transgenic *R1*, *R3a*, and *R3b* lines

**Figure 8:**
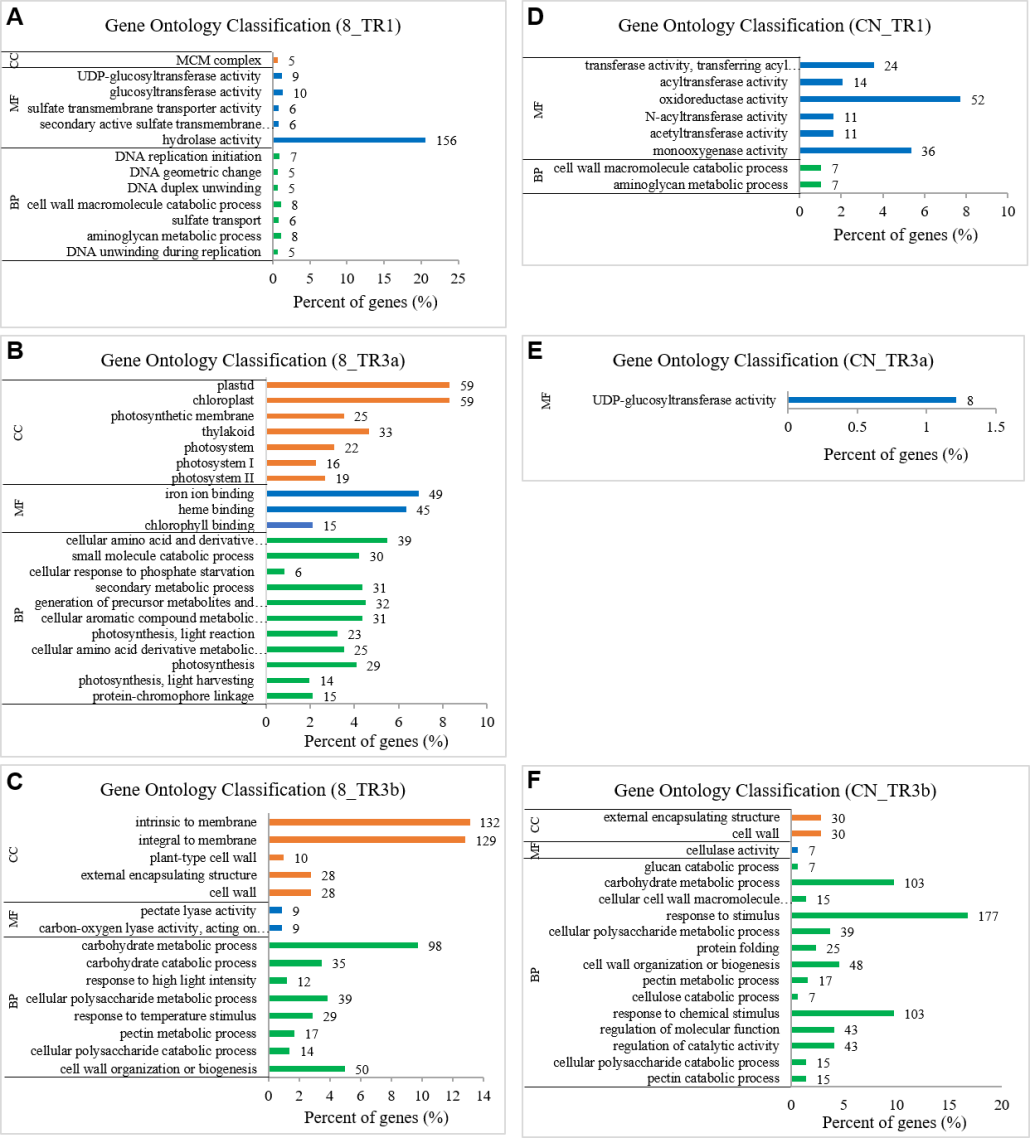
Specially enriched GO terms in transgenic *R1*, *R3a*, and *R3b* lines inoculated with *P. infestans* isolates 89148 and CN152. (A–C) GO terms in three transgenic lines under 89148. (D–F) GO terms in three transgenic lines under CN152. *Y*-axis, the three functional categories of GO terms: biological processes (BP), molecular functions (MF), and cellular components (CC). *X*-axis, the percentages of DEGs for each GO term. 8 = 89148, CN = CN152.

Among the specific GO terms in different transgenic lines, the top enriched GO term was unique to each transgenic line. Under 89148 infection, DNA unwinding during replication (GO:0006268, 5 DEGs) (Fig. 8A), protein-chromophore linkage (GO:0018298, 15 DEGs) (Fig. 8B), and cell wall organization or biogenesis (GO:0071554, 50 DEGs) (Fig. 8C) were the top significantly enriched terms in BP category in TR1, TR3a, and TR3b, respectively. In MF, hydrolase activity (GO:0016787) was the top term for TR1 with 156 DEGs (Fig. 8A). Further, the top terms in TR3a and TR3b were chlorophyll binding (GO:0016168, 15 DEGs) (Fig. 8B) and carbon-oxygen lyase activity, acting on polysaccharides (GO:0016837, 9 DEGs) (Fig. 8C), respectively.

When inoculated with CN152, the top significantly enriched biological process and molecular function in TR1 were aminoglycan metabolic process (GO: 0006022) and monooxygenase activity (GO: 0004497) with seven and 36 DEGs involved, respectively (Fig. 8D). In TR3a, only one molecular function GO term, UDP-glucosyltransferase activity (GO:0035251), was significantly enriched with specificity to TR3a (Fig. 8E). This term included eight DEGs encoding sucrose synthase, cellulose synthase and amylase synthase. Among these genes, *Arabidopsis* UDP-glycosyltransferase superfamily protein homologous gene PGSC0003DMG400012111 had the highest expression level across all genotypes, which was down-regulated in the transgenic lines compared to wild-type Desiree. Overexpression of the UDP-glucosyltransferase gene (*Ta-UGT 3*) can enhance resistance to *Fusarium* Head Blight in wheat (Xing et al., 2018). In TR3b, more GO terms were significantly enriched compared to TR1 and TR3a. The top three biological process terms for TR3b were pectin catabolism (GO:0045490, 15 DEGs), cellular polysaccharide catabolism (GO:0044247, 15 DEGs), and regulation of catalytic activity (GO:0050790, 43 DEGs). Among the molecular function GO terms, only cellulose activity (GO:0008810) was enriched with 7 DEGs (Fig. 8F).

**Figure 9:**
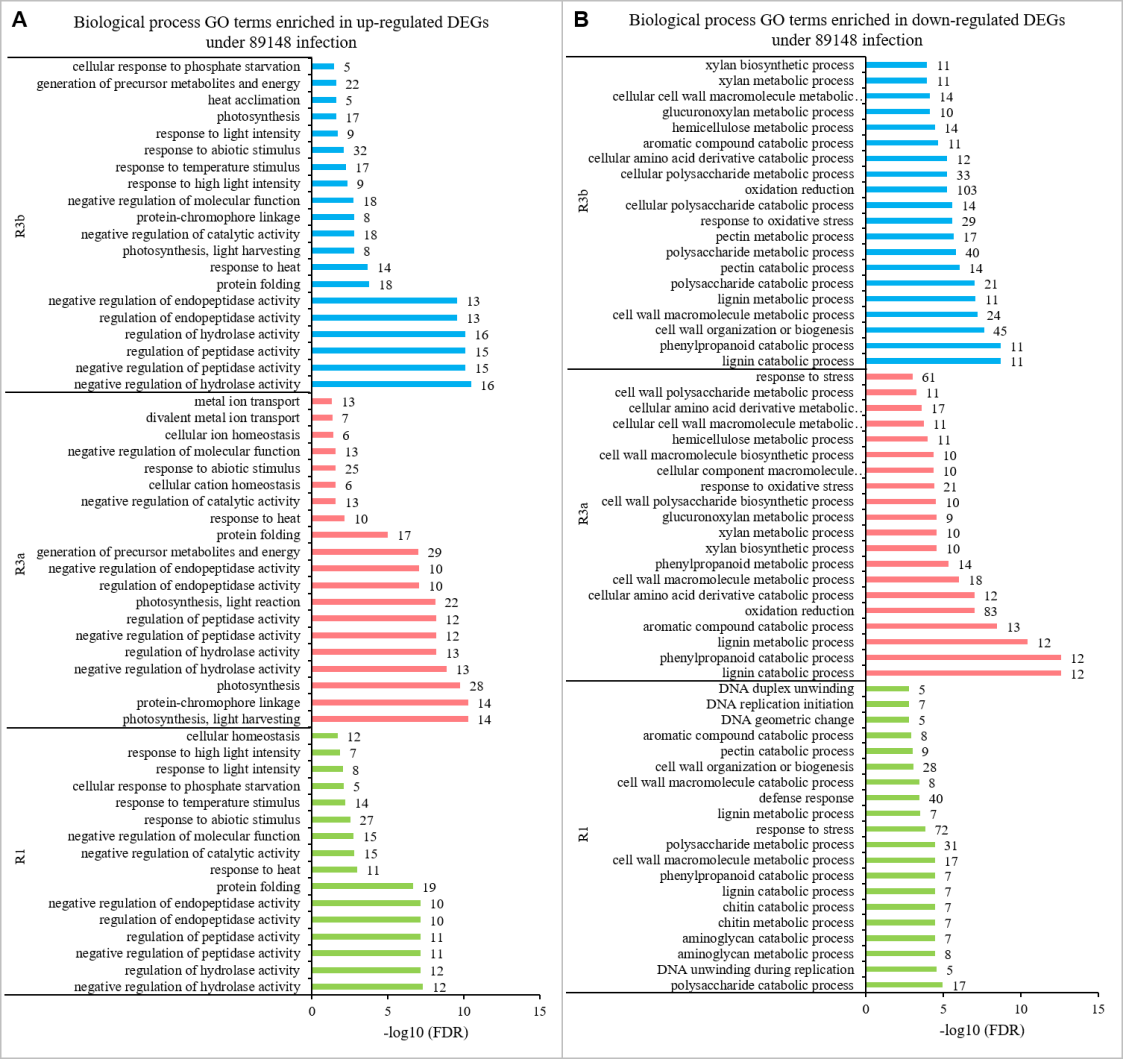
Biological processes enriched in up- and down-regulated genes in transgenic *R1*, *R3a*, and *R3b* lines by *P. infestans* isolate 89148 infection. (A) Biological processes enriched in up-regulated genes. (B) Biological processes enriched in down-regulated genes.

To further investigate the regulation mechanism of resistance-related genes, the up- or down-regulated DEGs in the GO enrichment were separately analyzed. Under 89148 inoculation, the 590, 544, and 880 up-regulated DEGs of the three transgenic lines were enriched in 20, 42, and 28 GO terms (Table 5), including 16, 20, and 20 biological process (BP) terms, respectively. The top 20 enriched BP terms are in Fig. 9. As with the enriched BP terms in up-regulated genes under 89148 infection, the main enriched BPs were positive or negative regulation of hydrolase activity, peptidase activity, and endopeptidase activity. The response to abiotic stimulus (GO: 0009628) had the most DEGs at 27, 25, and 32 for TR1, TR3a, and TR3b, respectively (Fig. 9A). More GO terms were involved in down-regulated genes than in up-regulated genes. The 728, 627, and 883 down-regulated DEGs of the three transgenic lines were enriched in 92, 51, and 73 GO terms (Table 5) which included 43, 36, and 48 BP terms, respectively. The BP terms of the three transgenic lines all contained response to stress (GO:000695) with 72, 61, and 74 DEGs, respectively (Fig. 9B, Table S8). In addition, 40 DEGs were enriched in defense response (GO: 0006952) in TR1. The highest numbers of DEGs enriched in TR3a and TR3a were oxidation reduction (GO: 0055114) at 83 and 103 DEGs. As above, after 89148 inoculation, the DEGs were mainly down-regulated and mediated the resistance of plants to *P. infestans* 89148 through response to stress (GO:000695) biological process.

**Table 5:** GO terms enriched in up- and down-regulated differentially expressed genes.

**DEG Sets**	**Up-regulated DEGs**			**Down-regulated DEGs**		
	**DEGs number**	**Enriched DEGs in GO**	**Significant GO terms (*FDR* < 0.05)**	**DEGs number**	**Enriched DEGs in GO**	**Significant GO terms (*FDR* < 0.05)**
8_TR1	590	324	20	728	433	92
8_TR3a	544	332	42	627	378	51
8_TR3b	880	438	28	883	569	73
CN_TR1	408	236	30	718	437	35
CN_TR3a	456	247	15	640	411	69
CN_TR3b	929	448	23	928	610	78

**Figure 10:**
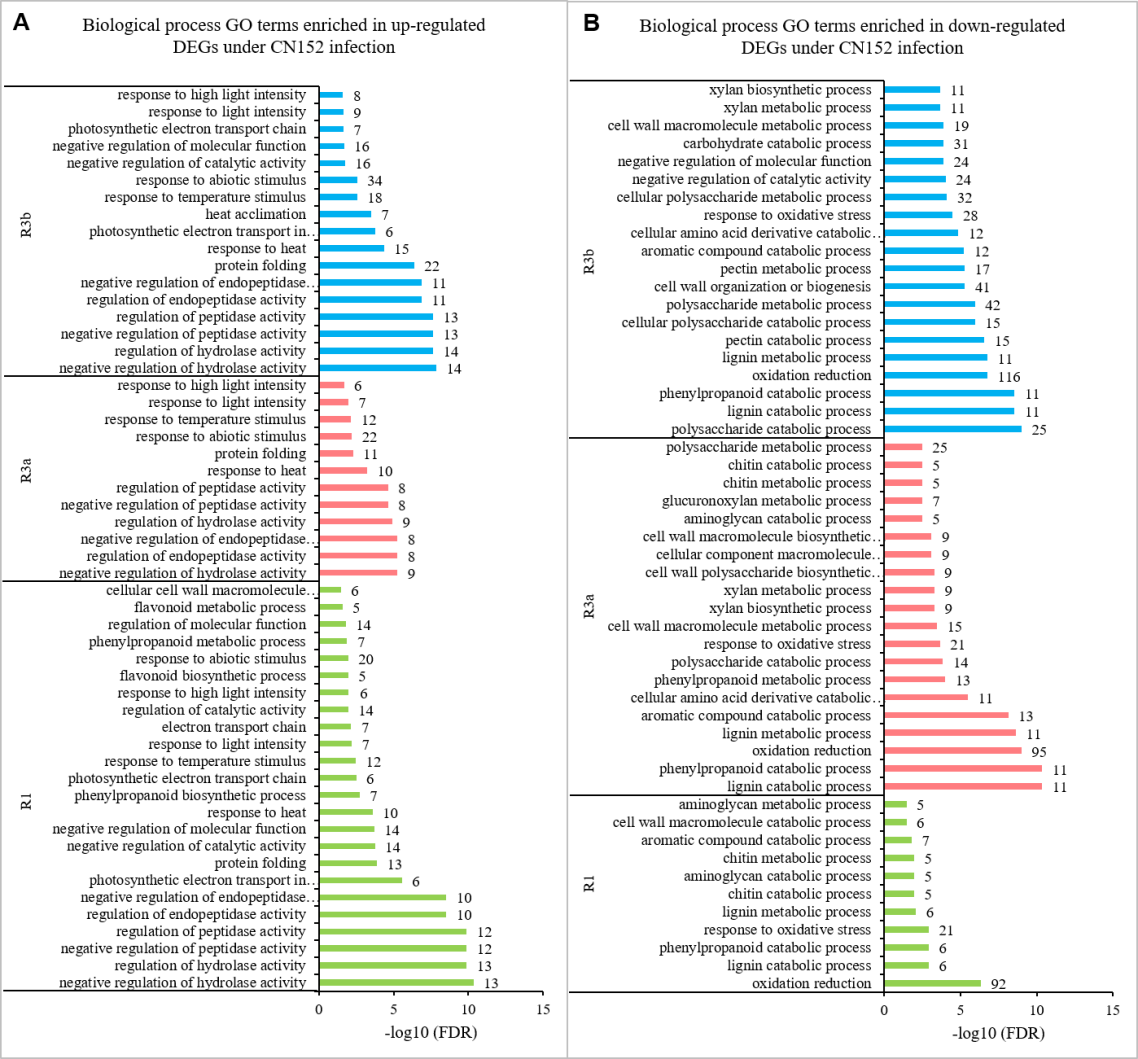
Biological processes enriched in up- and down-regulated genes in transgenic *R1*, *R3a*, and *R3b* lines by *P. infestans* isolate CN152 infection. (A) Biological processes enriched in up-regulated genes. (B) Biological processes enriched in down-regulated genes.

Under CN152 infection, the 408, 456, and 929 up-regulated DEGs of the three transgenic lines were enriched in 30, 15, and 23 GO terms (Table 5), including 24, 12, and 17 BP terms, respectively. The top 10–20 enriched BP terms are in Fig. 10. As with the enriched BP terms in up-regulated genes under CN152 infection, the main enriched BPs were also the positive or negative regulation of hydrolase activity, peptidase activity, and endopeptidase activity. The highest number of DEGs were also enriched in response to abiotic stimulus (GO: 0009628) with 20, 22, and 34 DEGs for TR1, TR3a, and TR3b, respectively (Fig. 10A). More GO terms were involved in down-regulated genes than in up-regulated genes. The 718, 640, and 928 down-regulated DEGs of the three transgenic lines were enriched in 35, 69, and 78 GO terms (Table 5), which included 11, 31, and 40 BP terms, respectively. The highest number of DEGs enriched in all three transgenic lines were oxidation reduction (GO: 0055114) at 92, 95, and 116 DEGs, respectively (Fig. 10B, Table S9). As above, the DEGs were mainly down-regulated and mediated the resistance of the plants to *P. infestans* strain CN152 through oxidation reduction biological process.

### Analysis of DEGs involved in incompatible/compatible reactions in transgenic lines in response to different *P. infestans* strains

**Figure 11:**
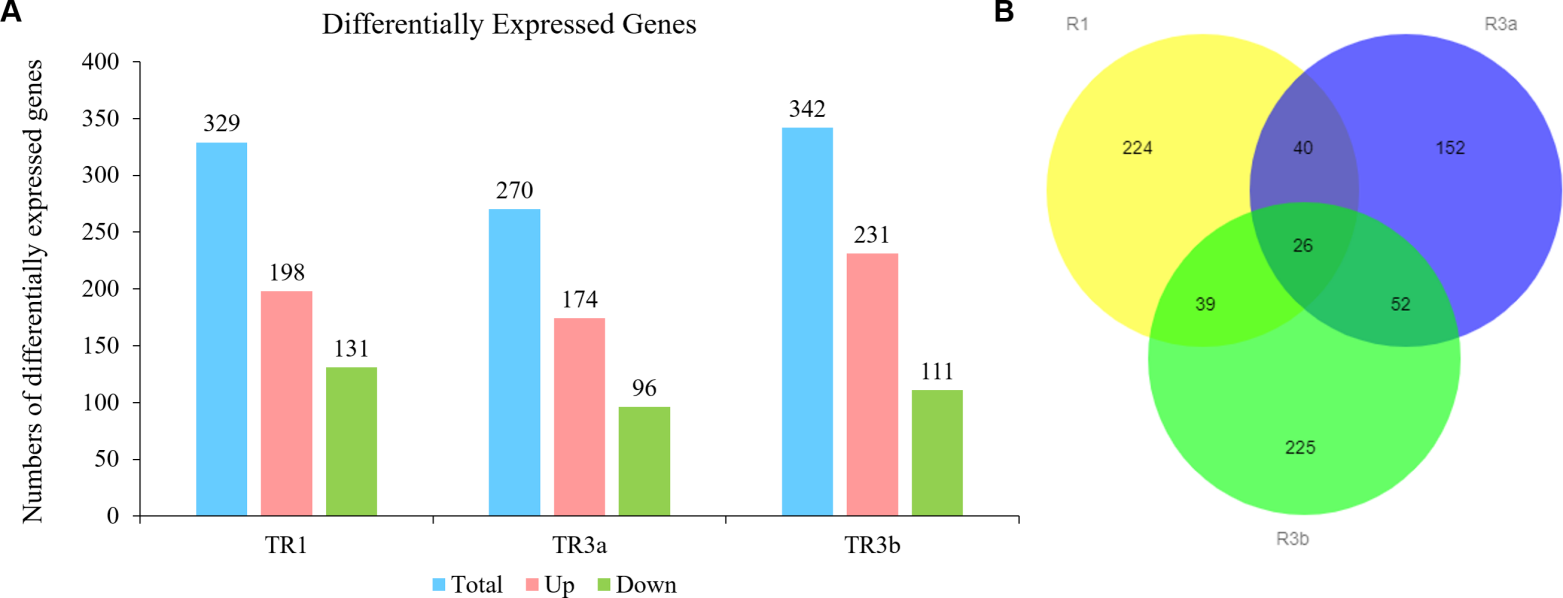
Differentially expressed genes (DEGs) involved in incompatible/compatible reactions in transgenic lines in response to different *P. infestans* isolates 89148 and CN152. (A) Histogram of DEGs numbers. (B) Venn diagrams of DEGs.

The genes expressed under 89148 infection were compared with those expressed under CN152 infection to identify the specific DEGs involved in incompatible/compatible reactions in the same transgenic lines. TR3b had the most DEGs with 342, followed by TR1 (329 DEGs). TR3a had the least DEGs at 270 (Fig. 11A). This indicated that *R3a* had the strongest resistance and *R3b* had the weakest resistance; the trend was the same as that generated by gene comparison between transgenic lines and wild-type Desiree under the same *P. infestans* strain infection. The number of up-regulated genes (198,174, 231 members, respectively) was higher than that of down-regulated genes (131, 96, 111 members, respectively) in all transgenic lines.

**Figure 12:**
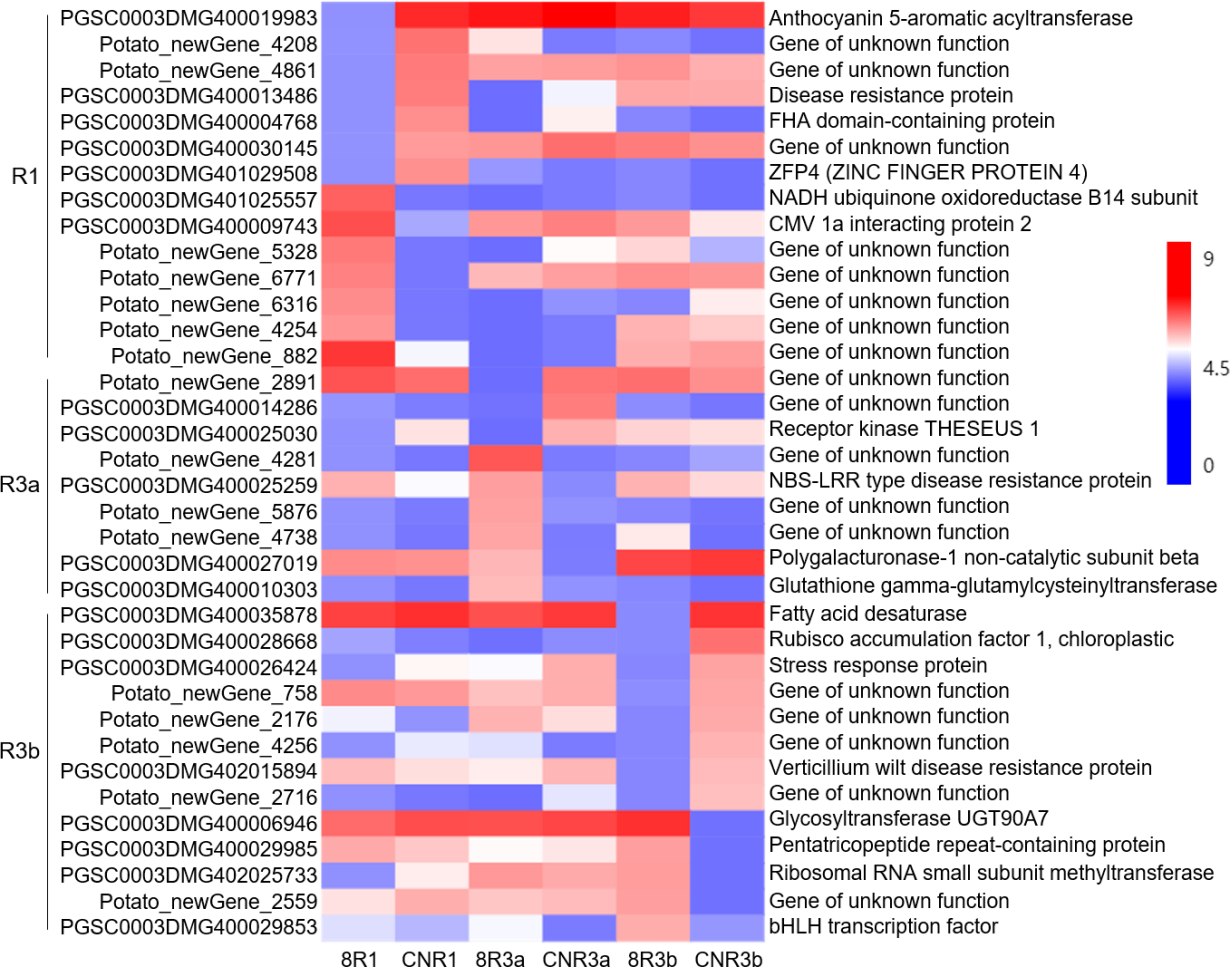
Highly differentially expressed genes (log2FC >5 or < − 5) in the same transgenic line with different inoculation of *P. infestans* isolates 89148 and CN152.

Venn diagrams showed that R1R3a, R1R3b, and R3aR3b shared 39, 40, and 52 genes, respectively. The unique DEGs were 224, 152, and 225 for TR1, TR3a, and TR3b (Fig. 11B). According to a value of log2FC >5 or < −5, a total of 14, nine, and 13 genes were highly differentially expressed in TR1, TR3a, and TR3b, respectively (Fig. 12). The 14 highly expressed DEGs for TR1 encoded anthocyanin 5-aromatic acyltransferase, disease resistance protein, and zinc finger protein 4. The nine highly expressed DEGs for TR3a encoded receptor kinase THESEUS 1, NBS-LRR type disease resistance protein, and polygalacturonase-1 non-catalytic subunit beta. The 13 highly expressed DEGs for TR3b encoded fatty acid desaturase, verticillium wilt disease resistance protein, and bHLH transcription factor. These highly differentially expressed genes may be essential in the incompatible/compatible reactions in transgenic lines response to different *P. infestans* strains 89148 and CN152.

**Figure 13:**
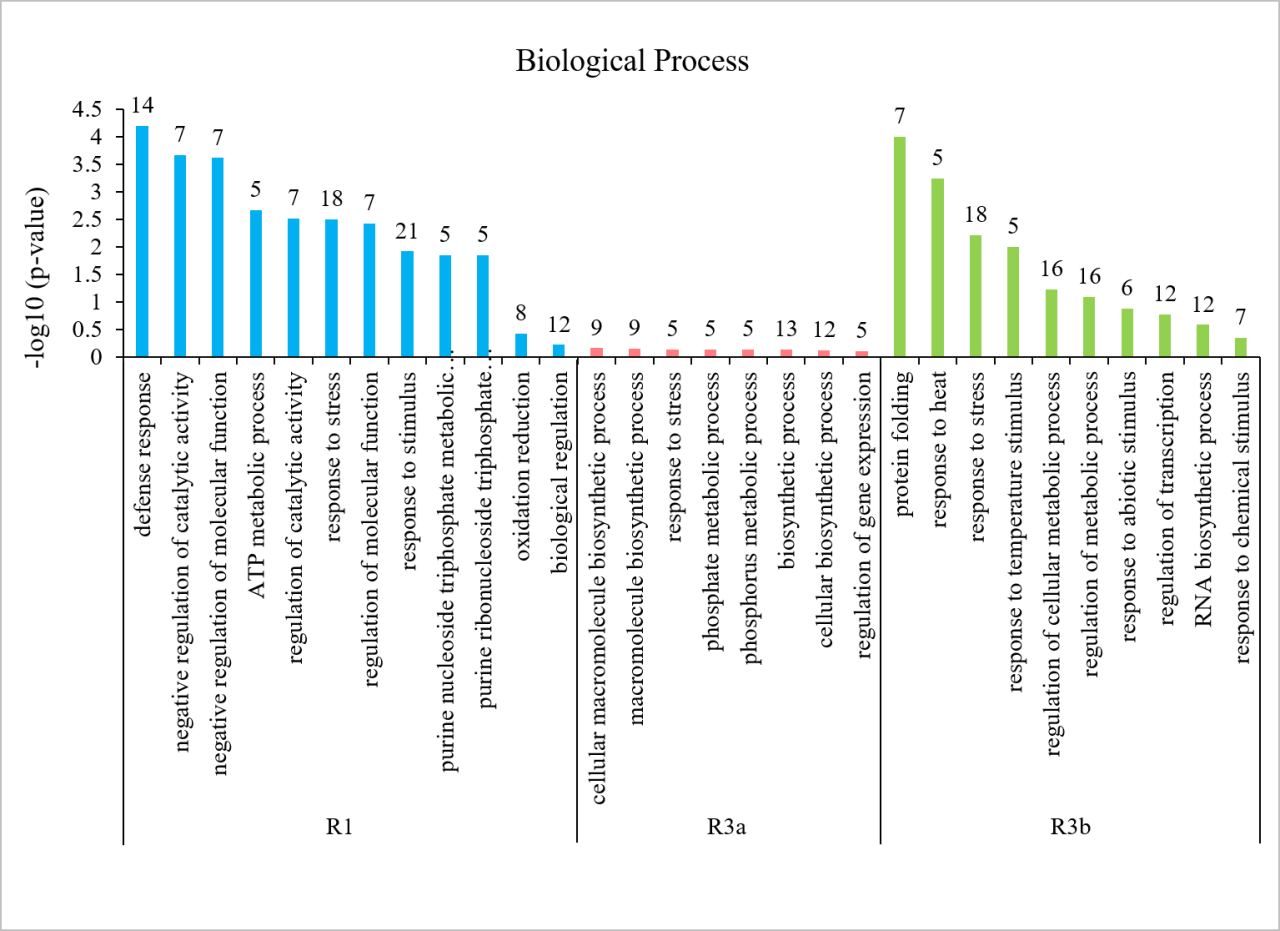
The top ten biological processes involved in incompatible/compatible reactions in transgenic *R1*, *R3a*, and *R3b* lines response to different *P. infestans* isolates 89148 and CN152.

The 224, 152, and 225 unique DEGs were respectively enriched in 125, 61, and 113 GO terms for TR1, TR3a, and TR3b. A total of 31 GO items were significantly (*p* <0.05) enriched in TR1 including the biological processes of defense response (GO:0006952, 14 DEGs), response to stress (GO:0006950, 18 DEGs), and response to stimulus (GO:0050896, 21 DEGs). No GO terms were significantly enriched in TR3a, which may be related to TR3a having the fewest DEGs. The top biological process in TR3a was oxidation reduction (GO:0055114). A total of 12 GO terms were significantly enriched in TR3b including response to heat (GO:0009408, 5 DEGs) and response to stress (GO:0006950, 18 DEGs) (Fig. 13). The top 10 biological process GO terms were listed in Fig. 13. Among the genes involved in response to stress biological process, 18 DEGs for TR1 mainly encoded NBS-LRR resistance proteins and disease resistance proteins; five DEGs for TR3a encoded NBS-LRR type disease resistance protein and ethylene-responsive late embryogenesis; 18 DEGs for TR3b mainly encoded pentatricopeptide repeat protein, p-coumarate 3-hydroxylase, and ATP binding protein (Table 6). These specific DEGs may function directly in the response to biotic stress by different *P. infestans* strains.

**Table 6:** The differentially expressed genes enriched in response to stress in three transgenic lines.

**Lines**	**Gene ID**	**log2FC**	**Regulated**	**Description**
TR1	PGSC0003DMG400001981	2.04	up	NBS-LRR protein
	PGSC0003DMG400002275	1.43	up	Cc-nbs-lrr resistance protein
	PGSC0003DMG400003056	−1.40	down	Ethylene-responsive proteinase inhibitor 1
	PGSC0003DMG400004561	1.62	up	Late blight resistance protein Rpi-blb2
	PGSC0003DMG400004873	−1.41	down	Resistance protein PSH-RGH6
	PGSC0003DMG400007514	−3.28	down	Glycolate oxidase
	PGSC0003DMG400009817	−1.20	down	Major latex
	PGSC0003DMG400011332	1.38	up	Conserved gene of unknown function
	PGSC0003DMG400013486	6.11	up	Disease resistance protein
	PGSC0003DMG400017557	1.32	up	AT hook motif family protein
	PGSC0003DMG400018461	1.21	up	Resistance gene
	PGSC0003DMG400018462	1.04	up	Disease resistance protein
	PGSC0003DMG400018903	3.06	up	Cc-nbs-lrr resistance protein
	PGSC0003DMG400031318	1.59	up	Tir-nbs-lrr resistance protein
	PGSC0003DMG400033356	−1.11	down	Sn-1 protein
	PGSC0003DMG401008349	1.81	up	Cc-nbs-lrr resistance protein
	PGSC0003DMG401030236	−2.30	down	CC-NBS-LRR protein
	PGSC0003DMG402019343	2.90	up	DNA binding protein
TR3a	PGSC0003DMG400000066	1.61	up	Ethylene-responsive late embryogenesis
	PGSC0003DMG400004211	−2.17	down	Photosystem Q(B) protein
	PGSC0003DMG400016270	1.35	up	Heat stress transcription factor A-6b
	PGSC0003DMG400025259	−5.88	down	NBS-LRR type disease resistance protein
	PGSC0003DMG400026433	1.00	up	ATP binding protein
TR3b	PGSC0003DMG400003289	−4.20	down	P-coumarate 3-hydroxylase
	PGSC0003DMG400003530	1.14	up	Abscisic acid and environmental stress-inducible protein TAS14
	PGSC0003DMG400005062	1.90	up	Peroxidase
	PGSC0003DMG400005573	1.11	up	Heat shock protein 83
	PGSC0003DMG400009255	1.55	up	Small heat-shock protein homolog protein
	PGSC0003DMG400014589	1.12	up	Calmodulin binding protein
	PGSC0003DMG400015035	−1.07	down	Peroxidase
	PGSC0003DMG400024539	1.16	up	ATEXO70E2
	PGSC0003DMG400024644	1.07	up	101 kDa heat shock protein
	PGSC0003DMG400024707	1.10	up	Luminal binding protein
	PGSC0003DMG400026724	−3.19	down	ATP binding protein
	PGSC0003DMG400028624	1.64	up	Small heat-shock protein
	PGSC0003DMG400029718	1.98	up	DNA binding protein
	PGSC0003DMG400029985	−5.44	down	EMB2744
	PGSC0003DMG400030339	1.90	up	17.6 kD class I small heat shock protein
	PGSC0003DMG401007628	1.10	up	Polyribonucleotide nucleotidyltransferase
	PGSC0003DMG401028907	1.01	up	Heat shock protein 83
	PGSC0003DMG402000506	2.04	up	Alpha-DOX2

### Clustering of cloned late blight resistance genes

**Figure 14:**
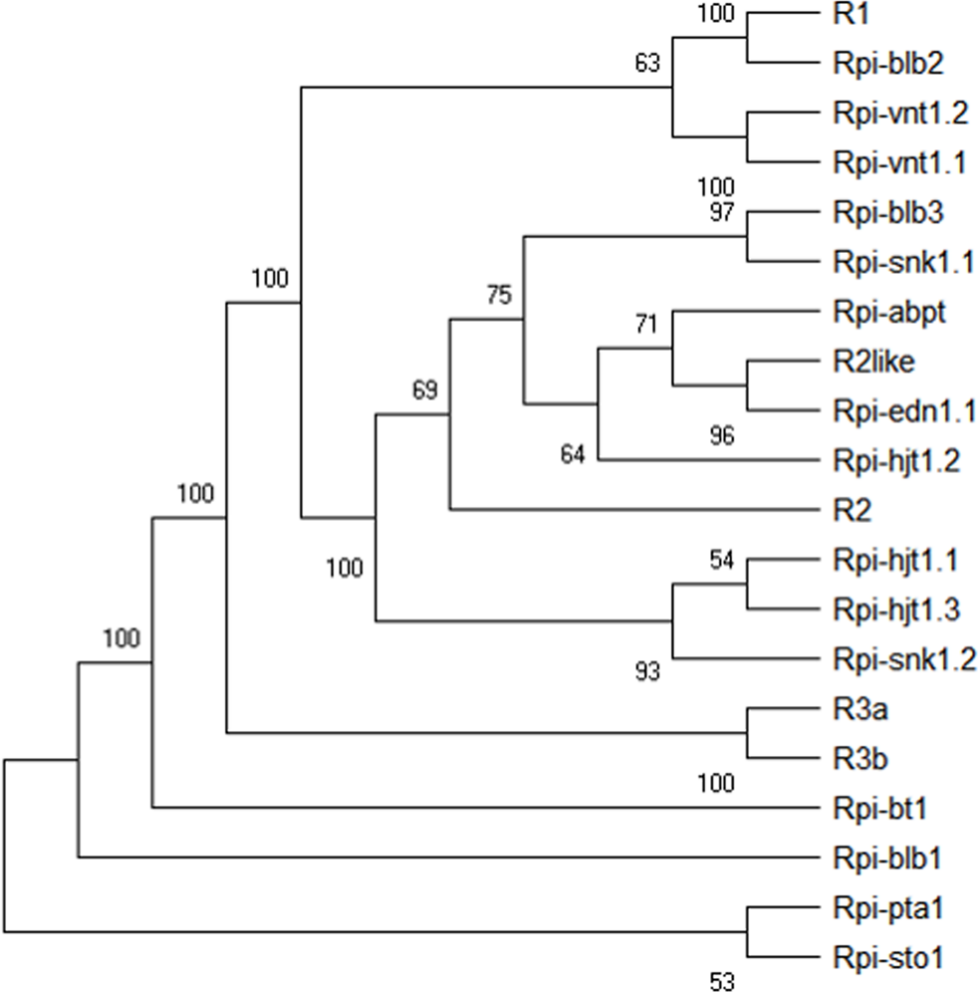
Cluster diagram of the cloned late blight resistance genes based on the CDS. Numbers on each node indicate bootstrap values of 1,000 replicates.

Cluster analysis of 20 cloned late blight resistance genes including *R1*, *R3a* and *R3b* was performed based on the complete CDS sequences using MEGA5 software. The cluster diagram showed that *R3a* and *R3b* were clustered together and supported with a 100% bootstrap value, while *R1* was distant from *R3a* and *R3b*, and clustered with *Rpi-blb2* gene in a separate branch with a 100% bootstrap value (Fig. 14). The clustering relationships of cloned late blight resistance genes were consistent with the results of gene expression profiling.

### Validation of RNA-seq data by real-time quantitative PCR (qRT-PCR)

**Figure 15:**
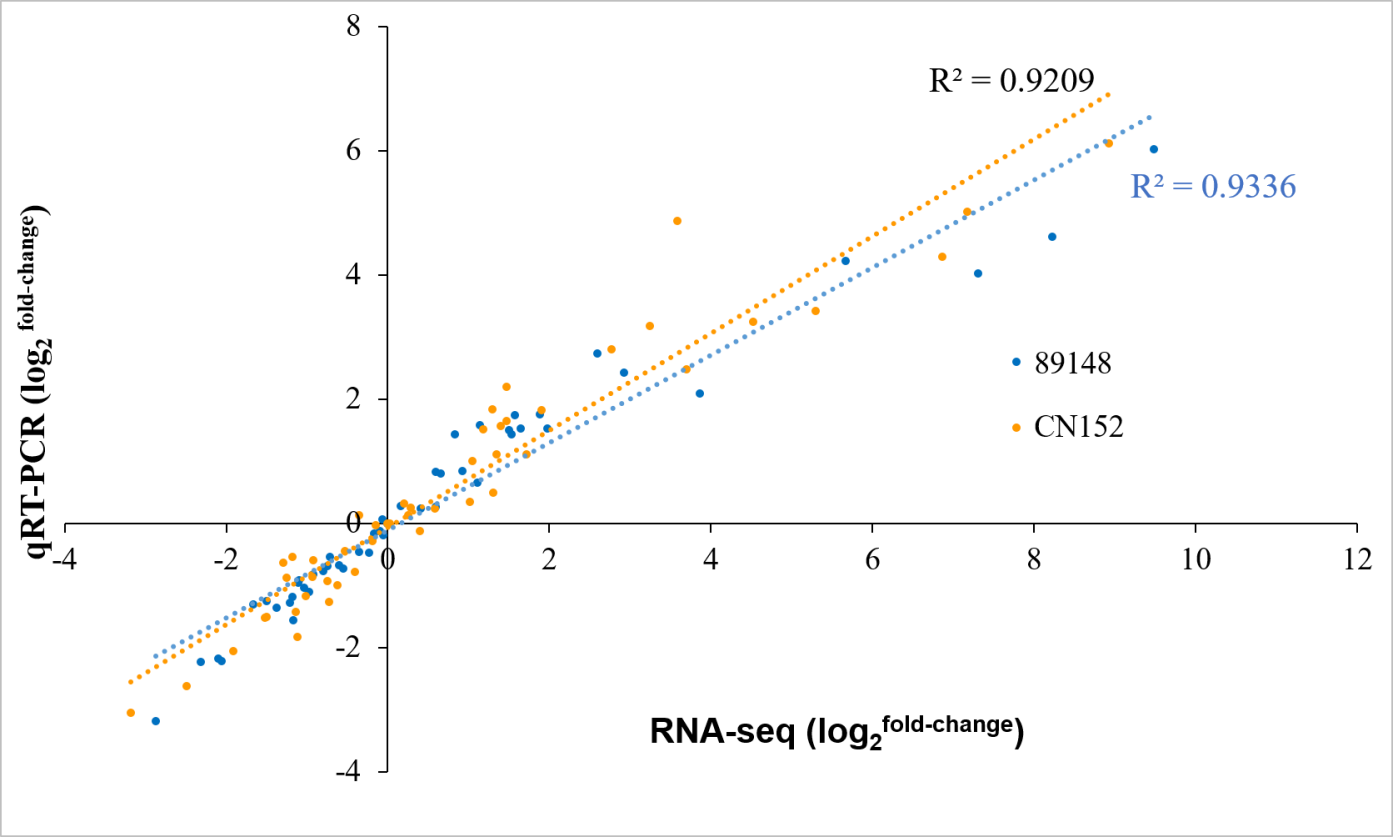
Correlation in fold-change of gene expression determined from RNA-seq data and data obtained using qRT-PCR. *X*-axis: Log2(fold-change) for gene expression data obtained with RNA-seq. *Y*-axis: Log2(fold-change) for gene expression data obtained using qRT-PCR. Each dot represents the expression analysis of a single gene. Blue dots represent gene profiles in response to *P. infestans* isolate 89148. Yellow dots represent gene profiles in response to *P. infestans* isolate CN152.

To validate the results of RNA-seq data, we randomly selected 16 DEGs in potato *R1*-, *R3a*-, and *R3b*-expressing transgenic plants 24 h after infection by *P. infestans* isolates 89148 and CN152, including ERF transcription factor 5, zinc finger protein and leucine-rich repeat family protein coding genes (Table S3). Quantitative RT-PCR was performed on these 16 DEGs. Gene expression was altered in each of the transgenic lines relative to WT Desiree plants. The fold changes of PGSC0003DMG400008364, PGSC0003DMG400000417, PGSC0003DMG400010128, and PGSC0003DMG400010139 genes were over 10 with log2 >3.4 (Table S4). The measured relative expression levels of these 16 DEGs were similar and a high correlation (*R*^2^>0.92) was observed between qRT-PCR and RNA-seq data (Fig. 15), confirming the reliability of the transcriptome data. The comparison of RNA-seq to log2 qRT-PCR levels is shown in Table S10.

## Discussion

*R1*, *R3a,* and *R3b* are race-specific resistance genes derived from the same wild potato species, *S. demissum*, but whether similar defense pathways are involved is not clear. In this study, the potato cultivar Desiree with high susceptibility to late blight, and its transgenic *R1*, *R3a,* and *R3b* lines, were inoculated with *P. infestans* race 89148 and super race CN152. Then the comparative transcriptome profiling and gene expression analysis were conducted 24 h post infection. The shared and unique aspects of resistance metabolic pathways mediated by *R1*, *R3a* and *R3b* genes were compared and analyzed. These results provide a theoretical basis for discovering the compatible and incompatible interaction mechanisms of potato and *P. infestans*, and for using molecular breeding to confer resistance to late blight in potato. Moreover, each plant differs only by the presence of a single *R* gene. Single *R* gene overexpression transgenic lines with the shared genetic background of variety Desiree (*RPi*-gene free cultivar) are ideal material for exploring the resistance and defense mechanisms mediated by *R* genes in potato.

This study would have benefited from a time series study and is now limited to 24 h infection; 24 hpi is a critical point for pathogen invasion. Within 24 hpi, spores germinate and form infection-specific appressoria to penetrate leaf cuticles and invade epidermal cells (Wei et al., 2013).

After infection by *P. infestans*, different numbers of DEGs were identified in the different transgenic lines relative to WT Desiree plants, indicating that *R1*, *R3a*, and *R3b* mediated resistance to *P. infestans* by regulating the expression of up- and down-stream genes. Under both inoculated conditions, the most DEGs were generated in TR3b, followed by TR1, and the fewest DEGs in TR3a. This may be related to the resistance levels of *R3a*>*R1*>*R3b*. Due to the low resistance level of *R3b*, expression of more genes may be necessary to regulate resistance. *R3a* has the strongest resistance; it may require expression of fewer defense genes to achieve a sufficient defense response.

In plant-pathogen incompatible interactions, disease resistance response in plants begins from the specific recognition between plant resistance (*R*) genes and the corresponding avirulence (*avr*) gene from the pathogen. In compatible interactions between host and pathogen, infection is successful and leads to symptom development. The isolate 89148 contained *Avr3a*, *Avr3b,* and *Avr10* and can clearly induce strong resistance responses and caused local cell death (HR) on potato differentials carrying *R3a*, *R3b,* and *R10* (Rietman, 2011). In the present study, we also found that the transgenic *R1*, *R3a*, and *R3b* lines showed resistance to isolate 89148, while all showed susceptibility to super race CN152. These results suggested that race 89148 mediated plant-pathogen incompatible interactions while CN152 mediated compatible interactions. The effector of 89148 initiates a single defense pathway in all of the tested transgenic *R* gene lines. *R1*, *R3a,* and *R3b* genes specifically recognized the corresponding *avr* genes in 89148 and produced HR.

Previous research showed more differential expression of mRNA in incompatible interactions of *Arabidopsis thaliana* with *Pseudomonas spp.* (Tao et al., 2003). More expressed putative *R*-genes were identified in resistant clones than in the susceptible clone (Frades et al., 2015). Our study also found that more DEGs were identified in 89148-mediated incompatible interactions than in CN152-mediated compatible interactions in transgenic *R1* and *R3a* lines. In the transgenic *R3b* potato line, more genes were differentially expressed under CN152 infection than under infection by 89148. This is probably because *R3b* has the lowest resistance level compared to *R1* and *R3a*, and this line requires expression changes at additional genes to respond to CN152 infection. On the other hand, this may indicate that timing of the defense response varied considerably between individual plants, with some responding faster than others.

According to the KEGG pathway analysis, more than 70% of the identified pathways were shared by *R1*, *R3a*, and *R3b*, regardless of pathogen genotype. This indicated that most defense pathways of the three *R* genes were similar, but still had minor differences. The top enriched pathway was unique to each transgenic line. Under 89148 infection, specific pathways of DNA replication (sot03030), plant-pathogen interaction (sot04626), and pentose and glucuronate interconversions (sot00040) were enriched in TR1, TR3a, and TR3b, respectively. The differences in the three transgenic lines may also be due to the speed of activation of different pathways rather than true differences between lines. MCM proteins are a family (MCM2-7) of six highly conserved and highly homologous proteins, which form a part of the pre-replication complex that licenses DNA replication. Mcm2-7 have been reported as useful proliferation markers in dysplasia and cancer in various tissues (Padmanabhan et al., 2004). In our study, *MCM4*, *MCM5*, *MCM6* and *MCM7* genes were all down-regulated, which may have negative regulatory in DNA replication in TR1 lines.

In the plant-pathogen interaction pathway for TR3a lines, the enriched DEGs encoding PR-1, HSP, CDPK, and CC-NBS-LRR proteins indicated *R3a* initiated the plant-pathogen interaction process in the transgenic *R3a* lines. Pectin is a main component of the primary cell wall, and is required to maintain the physical structure stability and mechanical strength of the cell wall. Pectate lyases are employed by pathogenic bacteria to degrade host tissue and to provide nutrients for bacterial growth. Silencing of *SlPL*, which encodes a pectate lyase in tomato, confers enhanced fruit firmness, prolonged shelf-life and reduced susceptibility to grey mold (Yang et al., 2017). All nine pectate lyase genes were down-regulated in our study, indicating that these genes have important negative regulatory roles in TR3b lines.

Regardless of whether potatoes were inoculated with isolate 89148 or CN152, the most common GO terms were enriched in transgenic *R3a* and *R3b* lines. This indicates that the defense pathways mediated by *R3a* and *R3b* are more similar than those mediated by *R1* and *R3a*, or *R1* and *R3b* in both incompatible and compatible interactions of potato and *P. infestans*.

The study may be limited since it is only based on RNA-seq of eight samples, but each sample was a mixture of ten different plants, and the pooled samples are better to eliminate the differences between backgrounds. Moreover, the quantitative real-time PCR experiment confirmed the induced expression of DEGs in the late blight resistance signaling pathway. Despite the limitation of non-replication of the eight samples the data does show that defense genes are activated.

Our results lay a solid foundation for further understanding the mechanisms of plant-pathogen interactions, and provide a theoretical reference for durable resistance in potato. The clustering analysis of 20 cloned *R* gene sequences showed *R3a* and *R3b* were most similar, while *R1* was most distant, which also supports the result of similar resistance pathways used in *R3a* and *R3b*, with a unique pathway in *R1*.

Early sequence-based expression measured transcript abundance by counting short segments, known as tags, generated from the 3′end of a transcript. Tag-based methods include the serial analysis of gene expression (SAGE), LongSAGE, and massively parallel signature sequencing (MPSS) (Velculescu et al., 1995). A DeepSAGE analysis was conducted to compare the compatible and incompatible interaction by using the transgenic *R1* lines and wild type cultivar Desiree with *P. infestans* strain R208m^2^ race 4 (*Avr1*) infection (Gyetvai et al., 2012). The results generated two thirds of the expressed tags with low frequency, while one third did not match any known potato transcript sequence. The development of deep sequencing technology including RNA-seq, enables simultaneous sequencing of millions of molecules and hassled to advanced approaches for expression measurement. RNA-seq is preferable to microarrays and tag-based approaches since it provides more information such as alternative splicing and isoform-specific gene expression with very low background signal and a wider dynamic range of quantification (Wang, Gerstein & Snyder, 2009). Moreover, RNA-seq measures expression level with high accuracy and reproducibility (Marioni et al., 2008; Fu et al., 2009). Compared to directly detecting gene expression abundance by DeepSAGE, RNA-seq is preferred for detection of differential expression of genes.

## Conclusion

Transcriptome profiling was performed in three transgenic lines, TR1, TR3a, and TR3b, and wild-type Desiree under inoculation with two *P. infestans* isolates, 89148 (race 0) and CN152 (super race). The RNA-seq analysis results indicate that the defense pathways mediated by *R3a* and *R3b* are more similar than those mediated by *R1* and *R3a*. The down-regulated DEGs mainly functioned in mediating the resistance of potato to *P. infestans* 89148 by response to stress biological process and to CN152 by oxidation reduction biological process. DNA replication, plant-pathogen interaction, and pentose and glucuronate interconversion KEGG pathways are unique for transgenic *R1*, *R3a*, and *R3b* lines in incompatible interactions. Our results lay a solid foundation for further understanding of the mechanisms of plant-pathogen interactions, and provide a theoretical reference for durable resistance in potato.

##  Supplemental Information

10.7717/peerj.9096/supp-1 Table S1 Primer sequences used for quantitative real-time PCR

10.7717/peerj.9096/supp-2 Table S2 Differential expressed genes enriched in amino sugar and nucleotide sugar metabolism (sot00520) pathway specific for transgenic *R1*, *R3a*, and *R3b* lines under 89148 infection

10.7717/peerj.9096/supp-3 Table S3 Differential expressed genes enriched in the top KEGG pathways specific for transgenic *R1*, *R3a*, and *R3b* lines under 89148 infection

10.7717/peerj.9096/supp-4 Table S4 Differential expressed genes enriched in the top KEGG pathways specific for transgenic *R1*, *R3a*, and *R3b* lines under CN152 infection

10.7717/peerj.9096/supp-5 Table S5 The down-regulated differential expressed genes enriched in the KEGG pathway of plant-pathogen interaction (sot04626) specific for transgenic *R1*, *R3a*, and *R3b* lines under 89148 and CN152 infection

10.7717/peerj.9096/supp-6 Table S6 Differential expressed genes enriched in the common biological process of oxidation reduction (GO:0055114) for transgenic *R3a* and *R3b* lines under 89148 infection

10.7717/peerj.9096/supp-7 Table S7 Differential expressed genes enriched in the common biological process of response to stress (GO:0006950) for transgenic *R3a* and *R3b* lines under CN152 infection

10.7717/peerj.9096/supp-8 Table S8 The down-regulated differential expressed genes enriched in the biological process of response to stress (GO:0006950) for transgenic* R1*, *R3a*, and *R3b* lines under 89148 infection

10.7717/peerj.9096/supp-9 Table S9 The down-regulated differential expressed genes enriched in the biological process of oxidation reduction (GO:0055114) for transgenic* R1*, *R3a*, and *R3b* lines under CN152 infection

10.7717/peerj.9096/supp-10 Table S10 The comparison of RNA-Seq to log2 qRT-PCR levels

## Additional Information and Declarations

### Data Availability

The following information was supplied regarding data availability: The raw data are available in the Genome Sequence Archive (GSA) in the BIG DATA Center, Beijing Institute of Genomics (BIG), Chinese Academy of Sciences: CRA001611. .
