# Supplementary material for: Comparison of defense responses of transgenic potato lines expressing three different Rpi genes to specific Phytophthora infestans races based on transcriptome profiling
Source: PeerJ. 2020 May 5;8:e9096. doi: 10.7717/peerj.9096 (PMC7207217; doi:10.7717/peerj.9096)
Supplement: Table S1 [file peerj-08-9096-s001.docx]

**Table S1. Primer sequences used for quantitative real-time PCR.**

| **Gene** | **Forward primer sequence (5’-3’)** | **Reverse primer sequence (5’-3’)** | **Gene description** |
| --- | --- | --- | --- |
| PGSC0003DMG400008364 | AGCCAAGGACTCAAGAAA | ACCCGTTACAAACACCAC | 2,4-dienoyl-CoA reductase |
| PGSC0003DMG400000417 | TGGTGGTCCAACTACGGT | ATTGAAATGGGCTCCTGT | Superoxide dismutase [Cu-Zn] |
| PGSC0003DMG400010128 | TTTGGAAAGTGGGAGATT | GTAACAGGGCAATACAATAA | Serine protease inhibitor 7 |
| PGSC0003DMG400010139 | CCGATACGTCCACCACCT | CTCCGTCTCATATTTCTCAATT | Cysteine protease inhibitor 1 |
| PGSC0003DMG400015289 | GGTGGAGGAGGACAGACA | AGCCTTCGCATCAACATG | Proteinase inhibitor type-2 CEVI57 |
| PGSC0003DMG400011502 | TGCGGAGTGGTTTGCTGG | AGTCTCGGTCGGCGTTTC | PEP carboxylase kinase |
| PGSC0003DMG400018407 | CCGTTCATCTACTACTTTA | ACAAGGCATTTCTTTCAC | Zinc finger protein |
| PGSC0003DMG400004062 | TGAAGTGTCCTCGTTGCG | GGCTTTGAACGCTTGCTT | DOF domain class transcription factor |
| PGSC0003DMG400002042 | AACTCCCACCTACAACGC | TCAGACAACCTTCCAGCA | Phosphoenolpyruvate carboxykinase |
| PGSC0003DMG400004064 | CAAAGAAGCTCCCTGATG | CGAACCCGGTCTAAACG | Subtilisin inhibitor 1 |
| PGSC0003DMG400006226 | ACGCAAGTGAGACCGC | TCCTGCTGCCATAGTTC | Leucine-rich repeat family protein |
| PGSC0003DMG400010815 | GGTCCAGCAGAAGCACAT | CAAAGCAGCACTTACCCAT | PME inhibitor |
| PGSC0003DMG400020174 | CCAGGCTGTAACAAGGTG | CAGAATTAGGCACAGGGA | Transcription factor |
| PGSC0003DMG400023458 | TTTGCCTAAGGAGATTG | CGAAGTGGAAGAGGAGC | Phenylalanine ammonia-lyase |
| PGSC0003DMG400023619 | TTAATACTGGCGAAGAAGC | TCTAACAATTCAGCACCC | ERF transcription factor 5 |
| PGSC0003DMG400025263 | CACCGCCTTATTGAACAG | TGATGAGATTAGAGCCACC | Basic helix-loop-helix family protein |
| GAPDH | CTGGTGCTGACTTCGTCG | CTGGCTTGTATTCATTCTCG | Internal reference gene |
