# Supplementary material for: Comparison of defense responses of transgenic potato lines expressing three different Rpi genes to specific Phytophthora infestans races based on transcriptome profiling
Source: PeerJ. 2020 May 5;8:e9096. doi: 10.7717/peerj.9096 (PMC7207217; doi:10.7717/peerj.9096)
Supplement: Table S2 [file peerj-08-9096-s002.docx]

**Table S2. Differential expressed genes enriched in amino sugar and nucleotide sugar metabolism (sot00520) pathway specific for transgenic *R1*, *R3a*, and *R3b* lines under 89148 infection.**

| **Gene ID** | **Log2FC** | **Regulated** | **Gene annotation** | **Transgenic lines** |
| --- | --- | --- | --- | --- |
| PGSC0003DMG400000735 | 2.07 | up | Glucose-1-phosphate adenylyltransferase | TR1 |
| PGSC0003DMG400004659 | -1.49 | down | UDP-D-glucuronic acid 4-epimerase | TR1 |
| PGSC0003DMG400008673 | -2.21 | down | Endochitinase (Chitinase) | TR1 |
| PGSC0003DMG400008797 | -4.16 | down | Endochitinase | TR1 |
| PGSC0003DMG400014401 | -1.73 | down | Glycosyltransferase, CAZy family GT8 | TR1 |
| PGSC0003DMG400015269 | -1.85 | down | Reversibly glycosylated polypeptide GRP 2 | TR1 |
| PGSC0003DMG400025063 | -2.17 | down | Class IV chitinase | TR1 |
| PGSC0003DMG400026853 | -1.96 | down | Endochitinase 3 | TR1 |
| PGSC0003DMG400026854 | -1.54 | down | Endochitinase 2 | TR1 |
| PGSC0003DMG400029738 | -1.31 | down | LEXYL2 protein | TR1 |
| PGSC0003DMG400033882 | -1.04 | down | Acidic endochitinase | TR1 |
| PGSC0003DMG400040317 | -2.81 | down | Endochitinase | TR1 |
| PGSC0003DMG402001531 | -2.27 | down | Chitinase 134 | TR1 |
| PGSC0003DMG400000735 | 1.26 | up | Glucose-1-phosphate adenylyltransferase | TR3a |
| PGSC0003DMG400004659 | -1.34 | down | UDP-D-glucuronic acid 4-epimerase | TR3a |
| PGSC0003DMG400008673 | -1.79 | down | Endochitinase (Chitinase) | TR3a |
| PGSC0003DMG400008797 | -3.55 | down | Endochitinase | TR3a |
| PGSC0003DMG400014401 | -1.63 | down | Glycosyltransferase, CAZy family GT8 | TR3a |
| PGSC0003DMG400015269 | -1.33 | down | Reversibly glycosylated polypeptide GRP 2 | TR3a |
| PGSC0003DMG400026853 | -1.68 | down | Endochitinase 3 | TR3a |
| PGSC0003DMG400033882 | -1.24 | down | Acidic endochitinase | TR3a |
| PGSC0003DMG400040317 | -2.95 | down | Endochitinase | TR3a |
| PGSC0003DMG402001531 | -1.72 | down | Chitinase 134 | TR3a |
| PGSC0003DMG400000735 | 2.39 | up | Glucose-1-phosphate adenylyltransferase | TR3b |
| PGSC0003DMG400001529 | 1.37 | up | Acidic 27 kDa endochitinase | TR3b |
| PGSC0003DMG400004659 | -1.47 | down | UDP-D-glucuronic acid 4-epimerase | TR3b |
| PGSC0003DMG400008673 | -1.80 | down | Endochitinase (Chitinase) | TR3b |
| PGSC0003DMG400008797 | -4.15 | down | Endochitinase | TR3b |
| PGSC0003DMG400011772 | -1.25 | down | Mannose-6-phosphate isomerase | TR3b |
| PGSC0003DMG400014401 | -1.42 | down | Glycosyltransferase, CAZy family GT8 | TR3b |
| PGSC0003DMG400019063 | 1.08 | up | UDP-sulfoquinovose synthase | TR3b |
| PGSC0003DMG400019228 | -2.24 | down | Glycosyltransferase, CAZy family GT8 | TR3b |
| PGSC0003DMG400025063 | -1.71 | down | Class IV chitinase | TR3b |
| PGSC0003DMG400029738 | -1.22 | down | LEXYL2 protein | TR3b |
| PGSC0003DMG400031084 | 1.11 | up | Glucose-1-phosphate adenylyltransferase small subunit, chloroplastic/amyloplastic | TR3b |
| PGSC0003DMG400040317 | -2.87 | down | Endochitinase | TR3b |
| PGSC0003DMG402001531 | -2.67 | down | Chitinase 134 | TR3b |
