# Supplementary material for: Comparison of defense responses of transgenic potato lines expressing three different Rpi genes to specific Phytophthora infestans races based on transcriptome profiling
Source: PeerJ. 2020 May 5;8:e9096. doi: 10.7717/peerj.9096 (PMC7207217; doi:10.7717/peerj.9096)
Supplement: Table S3 [file peerj-08-9096-s003.docx]

**Table S3. Differential expressed genes enriched in the top KEGG pathways specific for transgenic *R1*, *R3a*, and *R3b* lines under 89148 infection.**

| **Gene ID** | **Log2FC** | **Regulated** | **Gene annotation** | **Transgenic lines and the top KEGG pathway** |
| --- | --- | --- | --- | --- |
| PGSC0003DMG400002190 | -1.28 | down | Minichromosome maintenance factor | TR1, DNA replication |
| PGSC0003DMG400009040 | -1.05 | down | DNA replication licensing factor MCM7 |  |
| PGSC0003DMG400011569 | 4.68 | up | Gene of unknown function |  |
| PGSC0003DMG400011837 | -1.38 | down | Minichromosome maintenance 5 protein |  |
| PGSC0003DMG400012290 | -1.16 | down | DNA primase |  |
| PGSC0003DMG400013633 | -1.11 | down | Mini-chromosome maintenance protein MCM6 |  |
| PGSC0003DMG400017439 | -1.01 | down | Alpha DNA polymerase |  |
| PGSC0003DMG400030968 | -1.04 | down | Minichromosome maintenance 4 protein |  |
| PGSC0003DMG402011204 | 4.63 | up | Gene of unknown function |  |
| PGSC0003DMG400002275 | -1.04 | down | Cc-nbs-lrr resistance protein | TR3a, Plant-pathogen interaction |
| PGSC0003DMG400004646 | 2.33 | up | Calcium-dependent protein kinase |  |
| PGSC0003DMG400005109 | -4.79 | down | PR-1 |  |
| PGSC0003DMG400005111 | -2.35 | down | PR1 protein |  |
| PGSC0003DMG400005573 | 1.02 | up | Heat shock protein 83 |  |
| PGSC0003DMG400006155 | 1.32 | up | Thermal hysteresis protein STHP-64 |  |
| PGSC0003DMG400006610 | 1.03 | up | Receptor serine-threonine protein kinase |  |
| PGSC0003DMG400009883 | -1.02 | down | Calcium-dependent protein kinase |  |
| PGSC0003DMG400012471 | -1.78 | down | Conserved gene of unknown function |  |
| PGSC0003DMG400014168 | -1.16 | down | Respiratory burst oxidase homolog protein C |  |
| PGSC0003DMG400016433 | -2.04 | down | Receptor protein kinase |  |
| PGSC0003DMG400023125 | 1.16 | up | Conserved gene of unknown function |  |
| PGSC0003DMG400023921 | -2 | down | Cytoplasmic small heat shock protein class I |  |
| PGSC0003DMG400024754 | -1.58 | down | Respiratory burst oxidase homolog protein B |  |
| PGSC0003DMG400028474 | -2.58 | down | Conserved gene of unknown function |  |
| PGSC0003DMG401028907 | 1.06 | up | Heat shock protein 83 |  |
| PGSC0003DMG400000293 | 2.14 | up | Pectinesterase | TR3b, Pentose and glucuronate interconversions |
| PGSC0003DMG400003871 | -1.39 | down | Pectate lyase |  |
| PGSC0003DMG400010771 | -1.81 | down | Pectate lyase |  |
| PGSC0003DMG400012640 | -2.76 | down | Pectate lyase P18 |  |
| PGSC0003DMG400015815 | -1.07 | down | Pectase lyase |  |
| PGSC0003DMG400015933 | -1.05 | down | Pectinesterase PPE8B |  |
| PGSC0003DMG400020156 | -1.1 | down | Pectase lyase |  |
| PGSC0003DMG400025967 | -1.78 | down | Pectinesterase |  |
| PGSC0003DMG400027070 | -2.21 | down | Pectate lyase |  |
| PGSC0003DMG400029645 | -1.35 | down | Pectase lyase |  |
| PGSC0003DMG400031065 | -2.88 | down | Pectate lyase |  |
| PGSC0003DMG402017934 | -1.2 | down | Pectinesterase |  |
| PGSC0003DMG402023481 | -2.24 | down | Pectate lyase |  |
| PGSC0003DMG403024767 | -1.31 | down | Pectinesterase |  |
