# Supplementary material for: Comparison of defense responses of transgenic potato lines expressing three different Rpi genes to specific Phytophthora infestans races based on transcriptome profiling
Source: PeerJ. 2020 May 5;8:e9096. doi: 10.7717/peerj.9096 (PMC7207217; doi:10.7717/peerj.9096)
Supplement: Table S4 [file peerj-08-9096-s004.docx]

**Table S4. Differential expressed genes enriched in the top KEGG pathways specific for transgenic *R1*, *R3a*, and *R3b* lines under CN152 infection.**

| **Gene ID** | **Log2FC** | **Regulated** | **Gene annotation** | **Transgenic lines and the top KEGG pathway** |
| --- | --- | --- | --- | --- |
| PGSC0003DMG400002111 | 3.4 | up | CYP86A33 fatty acid omega-hydroxylase | TR1, Cutin, suberine and wax biosynthesis |
| PGSC0003DMG400007113 | -1.12 | down | Fatty acyl-CoA reductase 3 |  |
| PGSC0003DMG400007405 | 1.69 | up | Acyl CoA reductase |  |
| PGSC0003DMG400008885 | -2.1 | down | Fatty acyl-CoA reductase 2 |  |
| PGSC0003DMG400031731 | 2.81 | up | Feruloyl transferase |  |
| PGSC0003DMG400001948 | -1.92 | down | Copalyl diphosphate synthase | TR3a, Diterpenoid biosynthesis |
| PGSC0003DMG400005698 | -1.13 | down | Gibberellin 3-oxidase |  |
| PGSC0003DMG400021095 | 1.5 | up | Gibberellin 2-oxidase 1 |  |
| PGSC0003DMG400027631 | -1.68 | down | Gibberellin 2-oxidase |  |
| PGSC0003DMG400027632 | -1.59 | down | Gibberellin 2-oxidase 2 |  |
| PGSC0003DMG400003871 | -1.54 | down | Pectate lyase | TR3b, Pentose and glucuronate interconversions |
| PGSC0003DMG400009178 | -1.05 | down | Pectinesterase |  |
| PGSC0003DMG400010771 | -1.67 | down | Pectate lyase |  |
| PGSC0003DMG400012640 | -2.64 | down | Pectate lyase P18 |  |
| PGSC0003DMG400015230 | -1.24 | down | Pectate lyase |  |
| PGSC0003DMG400015815 | -1.11 | down | Pectase lyase |  |
| PGSC0003DMG400020372 | -4.35 | down | Polygalacturonase 7 |  |
| PGSC0003DMG400025967 | -2.01 | down | Pectinesterase |  |
| PGSC0003DMG400029645 | -1.86 | down | Pectase lyase |  |
| PGSC0003DMG400031065 | -3.2 | down | Pectate lyase |  |
| PGSC0003DMG400031816 | -2.19 | down | Pectinesterase |  |
| PGSC0003DMG402017934 | -1.1 | down | Pectinesterase |  |
| PGSC0003DMG402023481 | -1.7 | down | Pectate lyase |  |
| PGSC0003DMG400029645 | 1.86 | down | Pectase lyase |  |
