# Supplementary material for: Comparison of defense responses of transgenic potato lines expressing three different Rpi genes to specific Phytophthora infestans races based on transcriptome profiling
Source: PeerJ. 2020 May 5;8:e9096. doi: 10.7717/peerj.9096 (PMC7207217; doi:10.7717/peerj.9096)
Supplement: Table S5 [file peerj-08-9096-s005.docx]

**Table S5. The down-regulated differential expressed genes enriched in the KEGG pathway of plant-pathogen interaction (sot04626) specific for transgenic *R1*, *R3a*, and *R3b* lines under 89148 and CN152 infection.**

| **Gene ID** | **Log2FC** | **Regulated** | **Gene annotation** | **Transgenic lines and *P. infestans* isolate** |
| --- | --- | --- | --- | --- |
| GSC0003DMG400002275 | -1.55 | down | Cc-nbs-lrr resistance protein | TR1, 89148 |
| PGSC0003DMG400005109 | -5.10 | down | PR-1 |  |
| PGSC0003DMG400005111 | -2.94 | down | PR1 protein |  |
| PGSC0003DMG400008089 | -1.08 | down | Cyclic nucleotide-gated ion channel 15 |  |
| PGSC0003DMG400010215 | -2.38 | down | Cysteine protease |  |
| PGSC0003DMG400012471 | -1.61 | down | Conserved gene of unknown function |  |
| PGSC0003DMG400014168 | -1.31 | down | Respiratory burst oxidase homolog protein C |  |
| PGSC0003DMG400023360 | -1.40 | down | WRKY transcription factor |  |
| PGSC0003DMG400023921 | -2.00 | down | Cytoplasmic small heat shock protein class I |  |
| PGSC0003DMG400028474 | -2.77 | down | Conserved gene of unknown function |  |
| PGSC0003DMG400002275 | -1.04 | down | Cc-nbs-lrr resistance protein | TR3a, 89148 |
| PGSC0003DMG400005109 | -4.79 | down | PR-1 |  |
| PGSC0003DMG400005111 | -2.35 | down | PR1 protein |  |
| PGSC0003DMG400009883 | -1.02 | down | Calcium-dependent protein kinase |  |
| PGSC0003DMG400010215 | -2.03 | down | Cysteine protease |  |
| PGSC0003DMG400012471 | -1.78 | down | Conserved gene of unknown function |  |
| PGSC0003DMG400014168 | -1.16 | down | Respiratory burst oxidase homolog protein C |  |
| PGSC0003DMG400016433 | -2.04 | down | Receptor protein kinase |  |
| PGSC0003DMG400023360 | -2.20 | down | WRKY transcription factor |  |
| PGSC0003DMG400023921 | -2.00 | down | Cytoplasmic small heat shock protein class I |  |
| PGSC0003DMG400024754 | -1.58 | down | Respiratory burst oxidase homolog protein B |  |
| PGSC0003DMG400028474 | -2.58 | down | Conserved gene of unknown function |  |
| PGSC0003DMG400005111 | -4.11 | down | PR1 protein | TR3b, 89148 |
| PGSC0003DMG400005109 | -5.10 | down | PR-1 |  |
| PGSC0003DMG400005745 | -1.13 | down | Calcium-binding allergen Ole e 8 |  |
| PGSC0003DMG400008089 | -1.16 | down | Cyclic nucleotide-gated ion channel 15 |  |
| PGSC0003DMG400010215 | -4.49 | down | Cysteine protease |  |
| PGSC0003DMG400012316 | -1.32 | down | Respiratory burst oxidase homolog protein A |  |
| PGSC0003DMG400014168 | -1.31 | down | Respiratory burst oxidase homolog protein C |  |
| PGSC0003DMG400016769 | -1.40 | down | Double WRKY type transfactor |  |
| PGSC0003DMG400026077 | -1.89 | down | Calcium-dependent protein kinase |  |
| PGSC0003DMG400028474 | -3.62 | down | Conserved gene of unknown function |  |
| PGSC0003DMG400030472 | -1.28 | down | TSI1 |  |
| PGSC0003DMG400001333 | -1.24 | down | Calcium ion binding protein | TR1, CN152 |
| PGSC0003DMG400004460 | -1.13 | down | Conserved gene of unknown function |  |
| PGSC0003DMG400005109 | -3.44 | down | PR-1 |  |
| PGSC0003DMG400005111 | -3.63 | down | PR1 protein |  |
| PGSC0003DMG400005909 | -2.52 | down | Regulator of gene silencing |  |
| PGSC0003DMG400006727 | -1.27 | down | Cyclic nucleotide-gated ion channel 15 |  |
| PGSC0003DMG400010724 | -1.15 | down | ERF transcription factor 4 |  |
| PGSC0003DMG400011633 | -1.35 | down | WRKY-type transcription factor |  |
| PGSC0003DMG400014168 | -1.12 | down | Respiratory burst oxidase homolog protein C |  |
| PGSC0003DMG400016433 | -1.69 | down | Receptor protein kinase |  |
| PGSC0003DMG400025259 | -3.69 | down | NBS-LRR type disease resistance protein |  |
| PGSC0003DMG400026077 | -1.24 | down | Calcium-dependent protein kinase |  |
| PGSC0003DMG400028474 | -2.05 | down | Conserved gene of unknown function |  |
| PGSC0003DMG400005111 | -1.76 | down | PR1 protein | TR3a, CN152 |
| PGSC0003DMG400005909 | -1.93 | down | Regulator of gene silencing |  |
| PGSC0003DMG400016433 | -1.98 | down | Receptor protein kinase |  |
| PGSC0003DMG400023921 | -1.98 | down | Cytoplasmic small heat shock protein class I |  |
| PGSC0003DMG400025259 | -6.57 | down | NBS-LRR type disease resistance protein |  |
| PGSC0003DMG400026077 | -1.17 | down | Calcium-dependent protein kinase |  |
| PGSC0003DMG400028474 | -1.82 | down | Conserved gene of unknown function |  |
| PGSC0003DMG400028904 | -1.02 | down | SGT1 |  |
| PGSC0003DMG402017989 | -1.08 | down | Cyclic nucleotide-gated calmodulin-binding ion channel |  |
| PGSC0003DMG400005109 | -3.38 | down | PR-1 | TR3b, CN152 |
| PGSC0003DMG400005111 | -2.76 | down | PR1 protein |  |
| PGSC0003DMG400005745 | -1.06 | down | Calcium-binding allergen Ole e 8 |  |
| PGSC0003DMG400005909 | -3.44 | down | Regulator of gene silencing |  |
| PGSC0003DMG400008089 | -1.10 | down | Cyclic nucleotide-gated ion channel 15 |  |
| PGSC0003DMG400009883 | -1.15 | down | Calcium-dependent protein kinase |  |
| PGSC0003DMG400011633 | -1.05 | down | WRKY-type transcription factor |  |
| PGSC0003DMG400014168 | -1.01 | down | Respiratory burst oxidase homolog protein C |  |
| PGSC0003DMG400016433 | -1.84 | down | Receptor protein kinase |  |
| PGSC0003DMG400016769 | -1.29 | down | Double WRKY type transfactor |  |
| PGSC0003DMG400025259 | -2.14 | down | NBS-LRR type disease resistance protein |  |
| PGSC0003DMG400026077 | -2.51 | down | Calcium-dependent protein kinase |  |
| PGSC0003DMG400028474 | -2.71 | down | Conserved gene of unknown function |  |
| PGSC0003DMG400030472 | -1.23 | down | TSI1 |  |
