# Supplementary material for: Comparison of defense responses of transgenic potato lines expressing three different Rpi genes to specific Phytophthora infestans races based on transcriptome profiling
Source: PeerJ. 2020 May 5;8:e9096. doi: 10.7717/peerj.9096 (PMC7207217; doi:10.7717/peerj.9096)
Supplement: Table S6 [file peerj-08-9096-s006.docx]

**Table S6. Differential expressed genes enriched in the common biological process of oxidation reduction (GO:0055114) for transgenic *R3a* and *R3b* lines under 89148 infection.**

| **Gene ID** | **Log2FC** | **Regulated** | **Gene annotation** | **Transgenic lines** |
| --- | --- | --- | --- | --- |
| PGSC0003DMG400000148 | -2.95 | down | Cytochrome P450 | TR3a |
| PGSC0003DMG400000417 | -2.05 | down | Superoxide dismutase |  |
| PGSC0003DMG400000505 | -2.32 | down | Alpha-DOX1 |  |
| PGSC0003DMG400000798 | -2.34 | down | Cytochrome P450 |  |
| PGSC0003DMG400001078 | 1.57 | up | Fatty acid hydroperoxide lyase |  |
| PGSC0003DMG400001774 | -1.69 | down | Peroxidase |  |
| PGSC0003DMG400002581 | 2.98 | up | Succinic semialdehyde reductase isofom2 |  |
| PGSC0003DMG400003032 | 1.23 | up | Cytochrome P450 |  |
| PGSC0003DMG400003091 | 1.08 | up | Leucoanthocyanidin dioxygenase |  |
| PGSC0003DMG400003305 | 1.17 | up | Cytochrome P450 |  |
| PGSC0003DMG400003512 | -2.72 | down | Laccase |  |
| PGSC0003DMG400003654 | -1.06 | down | Peroxidase |  |
| PGSC0003DMG400003748 | -3.26 | down | Peroxidase |  |
| PGSC0003DMG400003865 | 1.81 | up | Conserved gene of unknown function |  |
| PGSC0003DMG400003914 | 2.15 | up | Copper chaperone |  |
| PGSC0003DMG400004532 | 3.35 | up | Chloroplast ferredoxin I |  |
| PGSC0003DMG400004800 | -1.21 | down | Gene of unknown function |  |
| PGSC0003DMG400004822 | -2.07 | down | Oxidoreductase |  |
| PGSC0003DMG400004844 | -1.02 | down | Glucose-methanol-choline (Gmc) oxidoreductase |  |
| PGSC0003DMG400005273 | -1.02 | down | Peroxidase |  |
| PGSC0003DMG400005279 | -2.06 | down | Peroxidase |  |
| PGSC0003DMG400005359 | 2.11 | up | Sinapyl alcohol dehydrogenase 2 |  |
| PGSC0003DMG400005515 | -1.01 | down | Ascorbate oxidase |  |
| PGSC0003DMG400005698 | -1.62 | down | Gibberellin 3-oxidase |  |
| PGSC0003DMG400005805 | 1.03 | up | Photosystem I reaction center subunit |  |
| PGSC0003DMG400005817 | 3.31 | up | Cytochrome P450 |  |
| PGSC0003DMG400006367 | 2.83 | up | Cytochrome P450 92B1 |  |
| PGSC0003DMG400006386 | -3.07 | down | Peroxidase |  |
| PGSC0003DMG400006764 | -1.40 | down | Cytokinin oxidase/dehydrogenase |  |
| PGSC0003DMG400007180 | -1.21 | down | P-coumaroyl quinate/shikimate 3'-hydroxylase |  |
| PGSC0003DMG400007565 | -1.50 | down | Cytochrome P450 |  |
| PGSC0003DMG400007639 | -1.52 | down | Cytochrome P450 |  |
| PGSC0003DMG400008344 | -1.20 | down | Glutamate dehydrogenase A |  |
| PGSC0003DMG400008389 | -1.78 | down | Short chain alcohol dehydrogenase |  |
| PGSC0003DMG400008826 | -3.06 | down | Laccase |  |
| PGSC0003DMG400008898 | -1.64 | down | Laccase |  |
| PGSC0003DMG400008947 | -1.08 | down | Desacetoxyvindoline 4-hydroxylase |  |
| PGSC0003DMG400009621 | -1.07 | down | Cytochrome P450 |  |
| PGSC0003DMG400009637 | 1.04 | up | Sinapyl alcohol dehydrogenase 2 |  |
| PGSC0003DMG400009759 | -1.53 | down | Cytochrome P450 71D7 |  |
| PGSC0003DMG400010125 | 2.83 | up | Ferric-chelate reductase |  |
| PGSC0003DMG400010660 | -1.61 | down | Superoxide dismutase |  |
| PGSC0003DMG400010859 | -2.22 | down | Lipoxygenase |  |
| PGSC0003DMG400011019 | -2.98 | down | Diphenol oxidase |  |
| PGSC0003DMG400011254 | -1.79 | down | Gibberellin 2-oxidase 1 |  |
| PGSC0003DMG400011295 | 1.01 | up | Thylakoid membrane phosphoprotein 14 kDa, chloroplastic |  |
| PGSC0003DMG400011601 | 1.39 | up | 2,4-dienoyl-CoA reductase |  |
| PGSC0003DMG400011640 | -1.93 | down | Peroxidase |  |
| PGSC0003DMG400011950 | 1.06 | up | Chloroplast ferredoxin I |  |
| PGSC0003DMG400012224 | 1.02 | up | Electron carrier |  |
| PGSC0003DMG400012589 | -2.82 | down | Cationic peroxidase |  |
| PGSC0003DMG400012666 | 1.21 | up | Ribulose bisphosphate carboxylase small chain 2C, chloroplastic |  |
| PGSC0003DMG400013352 | -2.08 | down | Dopamine beta-monooxygenase |  |
| PGSC0003DMG400013696 | -1.66 | down | Cytochrome P450 |  |
| PGSC0003DMG400013879 | -1.00 | down | Quinone reductase family protein |  |
| PGSC0003DMG400014093 | 1.39 | up | Flavonol synthase |  |
| PGSC0003DMG400014095 | -1.11 | down | 2,4-dienoyl-CoA reductase |  |
| PGSC0003DMG400014168 | -1.16 | down | Respiratory burst oxidase homolog protein C |  |
| PGSC0003DMG400014867 | -2.28 | down | Peroxidase |  |
| PGSC0003DMG400015220 | 2.18 | up | Cytochrome P450 71A4 |  |
| PGSC0003DMG400015356 | 1.08 | up | NADPH:protochlorophyllide oxidoreductase |  |
| PGSC0003DMG400015484 | -2.93 | down | Laccase |  |
| PGSC0003DMG400015677 | 1.34 | up | Cinnamyl alcohol dehydrogenase |  |
| PGSC0003DMG400015678 | 1.26 | up | 10-hydroxygeraniol oxidoreductase |  |
| PGSC0003DMG400016043 | -3.89 | down | Cytochrome P450 |  |
| PGSC0003DMG400016287 | 1.76 | up | Desacetoxyvindoline 4-hydroxylase |  |
| PGSC0003DMG400017184 | 1.85 | up | E8 protein homolog |  |
| PGSC0003DMG400017237 | 1.57 | up | Desacetoxyvindoline 4-hydroxylase |  |
| PGSC0003DMG400017280 | 2.15 | up | CYP72A56 |  |
| PGSC0003DMG400018114 | -1.05 | down | ATFRO8/FRO8 |  |
| PGSC0003DMG400018565 | 1.36 | up | Alcohol dehydrogenase |  |
| PGSC0003DMG400018778 | -1.34 | down | 12-oxophytodienoate reductase 1 |  |
| PGSC0003DMG400018914 | -4.08 | down | Polyphenol oxidase |  |
| PGSC0003DMG400019185 | -2.50 | down | Diphenol oxidase |  |
| PGSC0003DMG400019718 | -1.66 | down | Amine oxidase |  |
| PGSC0003DMG400020252 | -2.01 | down | Peroxidase 55 |  |
| PGSC0003DMG400020345 | -3.63 | down | Diphenol oxidase |  |
| PGSC0003DMG400020355 | -2.26 | down | Conserved gene of unknown function |  |
| PGSC0003DMG400020467 | 4.35 | up | Conserved gene of unknown function |  |
| PGSC0003DMG400020618 | -1.44 | down | Tropinone reductase homolog |  |
| PGSC0003DMG400020799 | -2.40 | down | Cationic peroxidase 1 |  |
| PGSC0003DMG400021107 | 2.33 | up | Conserved gene of unknown function |  |
| PGSC0003DMG400021966 | 3.36 | up | Laccase |  |
| PGSC0003DMG400022025 | -1.81 | down | Flavin monooxygenase |  |
| PGSC0003DMG400022392 | -4.22 | down | Reticuline oxidase |  |
| PGSC0003DMG400022541 | -2.53 | down | Peroxidase 72 |  |
| PGSC0003DMG400022792 | 1.65 | up | Cytochrome B561 |  |
| PGSC0003DMG400023193 | -1.10 | down | Short chain alcohol dehydrogenase |  |
| PGSC0003DMG400024289 | -2.03 | down | Reticuline oxidase |  |
| PGSC0003DMG400024643 | 1.01 | up | Flavonoid 3'-monooxygenase |  |
| PGSC0003DMG400024754 | -1.58 | down | Respiratory burst oxidase homolog protein B |  |
| PGSC0003DMG400024967 | -1.35 | down | Peroxidase |  |
| PGSC0003DMG400026080 | -1.47 | down | Cytochrome P450 |  |
| PGSC0003DMG400026276 | -1.18 | down | Leucoanthocyanidin dioxygenase |  |
| PGSC0003DMG400026281 | 1.01 | up | Glutathione peroxidase |  |
| PGSC0003DMG400026409 | 1.32 | up | Ribulose bisphosphate carboxylase small chain 2B, chloroplastic |  |
| PGSC0003DMG400026575 | -3.02 | down | Class III peroxidase |  |
| PGSC0003DMG400027168 | -3.80 | down | Laccase 90a |  |
| PGSC0003DMG400027577 | 2.87 | up | Superoxide dismutase |  |
| PGSC0003DMG400027831 | -4.60 | down | IBR3 (IBA-RESPONSE 3) |  |
| PGSC0003DMG400028221 | 1.75 | up | Tropinone reductase I |  |
| PGSC0003DMG400029575 | -1.46 | down | Catechol oxidase B, chloroplastic |  |
| PGSC0003DMG400030376 | -3.17 | down | Laccase |  |
| PGSC0003DMG400030382 | -1.77 | down | Class III peroxidase |  |
| PGSC0003DMG400030413 | -2.50 | down | Cytochrome P450 |  |
| PGSC0003DMG400030419 | -5.25 | down | Conserved gene of unknown function |  |
| PGSC0003DMG400030551 | -1.01 | down | Spore coat protein |  |
| PGSC0003DMG400030998 | 1.05 | up | Alcohol dehydrogenase |  |
| PGSC0003DMG400031420 | -1.01 | down | Cytokinin oxidase/dehydrogenase |  |
| PGSC0003DMG400031479 | 1.55 | up | Cinnamyl alcohol dehydrogenase |  |
| PGSC0003DMG400031519 | -1.16 | down | Conserved gene of unknown function |  |
| PGSC0003DMG400032121 | -1.68 | down | Short chain alcohol dehydrogenase |  |
| PGSC0003DMG400032162 | -1.57 | down | Short chain alcohol dehydrogenase |  |
| PGSC0003DMG400032510 | -1.49 | down | L-ascorbate oxidase |  |
| PGSC0003DMG400033636 | -1.68 | down | Cytochrome P450 |  |
| PGSC0003DMG400033874 | -1.13 | down | Malic enzyme |  |
| PGSC0003DMG400033932 | -1.29 | down | Cytochrome P450 hydroxylase |  |
| PGSC0003DMG400044958 | 1.37 | up | Conserved gene of unknown function |  |
| PGSC0003DMG400046303 | -1.56 | down | Photosystem II CP47 chlorophyll apoprotein |  |
| PGSC0003DMG401000287 | -1.86 | down | Myo-inositol oxygenase |  |
| PGSC0003DMG401001731 | 1.62 | up | Ascorbate peroxidase |  |
| PGSC0003DMG401008895 | 1.05 | up | Short chain alcohol dehydrogenase |  |
| PGSC0003DMG401009494 | 4.13 | up | Ferric-chelate reductase |  |
| PGSC0003DMG401018777 | -1.23 | down | 12-oxophytodienoate reductase 1 |  |
| PGSC0003DMG401026767 | 1.25 | up | Delta 1-pyrroline-5-carboxylate synthetase |  |
| PGSC0003DMG401029332 | -3.40 | down | Peroxidase |  |
| PGSC0003DMG402000506 | 1.92 | up | Alpha-DOX2 |  |
| PGSC0003DMG402015497 | -3.64 | down | Pericarp peroxidase 3 |  |
| PGSC0003DMG402027116 | -3.55 | down | Laccase 90d |  |
| PGSC0003DMG400000277 | -2.61 | down | Cytokinin oxidase/dehydrogenase 2 | TR3b |
| PGSC0003DMG400000360 | 3.71 | up | Malic enzyme |  |
| PGSC0003DMG400000417 | -2.86 | down | Superoxide dismutase |  |
| PGSC0003DMG400000505 | -5.07 | down | Alpha-DOX1 |  |
| PGSC0003DMG400000511 | -1.10 | down | Class III peroxidase |  |
| PGSC0003DMG400000565 | -1.38 | down | Cytochrome P450 |  |
| PGSC0003DMG400000761 | -1.07 | down | Multicopper oxidase |  |
| PGSC0003DMG400000977 | 1.67 | up | Multicopper oxidase |  |
| PGSC0003DMG400001078 | 1.29 | up | Fatty acid hydroperoxide lyase |  |
| PGSC0003DMG400001249 | -1.49 | down | Gibberellin 20 oxidase |  |
| PGSC0003DMG400001774 | -2.79 | down | Peroxidase |  |
| PGSC0003DMG400001932 | -1.32 | down | 6-phosphogluconate dehydrogenase, decarboxylating |  |
| PGSC0003DMG400002351 | -1.19 | down | CYP72A58 |  |
| PGSC0003DMG400002528 | -1.20 | down | Downstream target of agl15-4 |  |
| PGSC0003DMG400002581 | 1.55 | up | Succinic semialdehyde reductase isofom2 |  |
| PGSC0003DMG400002612 | 1.21 | up | Malic enzyme |  |
| PGSC0003DMG400002967 | 1.26 | up | Pheophorbide A oxygenase |  |
| PGSC0003DMG400003091 | 1.72 | up | Leucoanthocyanidin dioxygenase |  |
| PGSC0003DMG400003305 | 1.62 | up | Cytochrome P450 |  |
| PGSC0003DMG400003512 | -2.84 | down | Laccase |  |
| PGSC0003DMG400003654 | -1.03 | down | Peroxidase |  |
| PGSC0003DMG400003748 | -2.53 | down | Peroxidase |  |
| PGSC0003DMG400003914 | 2.36 | up | Copper chaperone |  |
| PGSC0003DMG400004532 | 4.18 | up | Chloroplast ferredoxin I |  |
| PGSC0003DMG400004800 | -1.19 | down | Gene of unknown function |  |
| PGSC0003DMG400004822 | -2.47 | down | Oxidoreductase |  |
| PGSC0003DMG400004844 | -2.40 | down | Glucose-methanol-choline (Gmc) oxidoreductase |  |
| PGSC0003DMG400004872 | 2.40 | up | Myo-inositol oxygenase |  |
| PGSC0003DMG400005062 | -1.42 | down | Peroxidase |  |
| PGSC0003DMG400005273 | -1.43 | down | Peroxidase |  |
| PGSC0003DMG400005279 | -1.67 | down | Peroxidase |  |
| PGSC0003DMG400005359 | 2.72 | up | Sinapyl alcohol dehydrogenase 2 |  |
| PGSC0003DMG400005515 | -1.27 | down | Ascorbate oxidase |  |
| PGSC0003DMG400005698 | -1.78 | down | Gibberellin 3-oxidase |  |
| PGSC0003DMG400006159 | -1.42 | down | Dopamine beta-monooxygenase |  |
| PGSC0003DMG400006386 | -2.82 | down | Peroxidase |  |
| PGSC0003DMG400006516 | 3.57 | up | L-ascorbate oxidase homolog |  |
| PGSC0003DMG400006764 | -1.92 | down | Cytokinin oxidase/dehydrogenase |  |
| PGSC0003DMG400007169 | 3.64 | up | 2,4-dienoyl-CoA reductase |  |
| PGSC0003DMG400007180 | -1.22 | down | P-coumaroyl quinate/shikimate 3'-hydroxylase |  |
| PGSC0003DMG400007565 | -3.09 | down | Cytochrome P450 |  |
| PGSC0003DMG400007639 | -1.49 | down | Cytochrome P450 |  |
| PGSC0003DMG400008356 | -1.27 | down | NADH-glutamate dehydrogenase |  |
| PGSC0003DMG400008363 | 2.80 | up | 2,4-dienoyl-CoA reductase |  |
| PGSC0003DMG400008364 | 2.78 | up | 2,4-dienoyl-CoA reductase |  |
| PGSC0003DMG400008389 | -1.53 | down | Short chain alcohol dehydrogenase |  |
| PGSC0003DMG400008826 | -2.47 | down | Laccase |  |
| PGSC0003DMG400008898 | -2.10 | down | Laccase |  |
| PGSC0003DMG400008947 | -1.38 | down | Desacetoxyvindoline 4-hydroxylase |  |
| PGSC0003DMG400009623 | -1.02 | down | Cytochrome P450 71D7 |  |
| PGSC0003DMG400009637 | 1.47 | up | Sinapyl alcohol dehydrogenase 2 |  |
| PGSC0003DMG400009759 | -1.68 | down | Cytochrome P450 71D7 |  |
| PGSC0003DMG400010021 | -1.40 | down | Short-chain dehydrogenase |  |
| PGSC0003DMG400010025 | -1.25 | down | Multicopper oxidase |  |
| PGSC0003DMG400010125 | 3.91 | up | Ferric-chelate reductase |  |
| PGSC0003DMG400010465 | -4.52 | down | Peroxidase |  |
| PGSC0003DMG400010660 | -2.02 | down | Superoxide dismutase |  |
| PGSC0003DMG400010859 | -3.83 | down | Lipoxygenase |  |
| PGSC0003DMG400011019 | -2.11 | down | Diphenol oxidase |  |
| PGSC0003DMG400011098 | -2.95 | down | Oxidoreductase |  |
| PGSC0003DMG400011254 | -1.49 | down | Gibberellin 2-oxidase 1 |  |
| PGSC0003DMG400011601 | 1.47 | up | 2,4-dienoyl-CoA reductase |  |
| PGSC0003DMG400011640 | -2.10 | down | Peroxidase |  |
| PGSC0003DMG400012316 | -1.32 | down | Respiratory burst oxidase homolog protein A |  |
| PGSC0003DMG400012589 | -3.37 | down | Cationic peroxidase |  |
| PGSC0003DMG400013352 | -2.08 | down | Dopamine beta-monooxygenase |  |
| PGSC0003DMG400013629 | 1.52 | up | Cytochrome P450 hydroxylase |  |
| PGSC0003DMG400013696 | -2.69 | down | Cytochrome P450 |  |
| PGSC0003DMG400013879 | -1.38 | down | Quinone reductase family protein |  |
| PGSC0003DMG400014055 | -1.62 | down | Peroxidase |  |
| PGSC0003DMG400014093 | 1.73 | up | Flavonol synthase |  |
| PGSC0003DMG400014168 | -1.31 | down | Respiratory burst oxidase homolog protein C |  |
| PGSC0003DMG400014867 | -1.99 | down | Peroxidase |  |
| PGSC0003DMG400015220 | 2.47 | up | Cytochrome P450 71A4 |  |
| PGSC0003DMG400015484 | -2.97 | down | Laccase |  |
| PGSC0003DMG400015677 | 2.12 | up | Cinnamyl alcohol dehydrogenase |  |
| PGSC0003DMG400015861 | 1.46 | up | Photosystem Q(B) protein |  |
| PGSC0003DMG400016043 | -2.92 | down | Cytochrome P450 |  |
| PGSC0003DMG400016287 | 1.29 | up | Desacetoxyvindoline 4-hydroxylase |  |
| PGSC0003DMG400016623 | -1.12 | down | Cytochrome P450 |  |
| PGSC0003DMG400016785 | -1.04 | down | Dimethylaniline monooxygenase |  |
| PGSC0003DMG400016887 | 1.39 | up | Allene oxide synthase |  |
| PGSC0003DMG400017184 | 2.53 | up | E8 protein homolog |  |
| PGSC0003DMG400017842 | 1.36 | up | Cinnamoyl-CoA reductase |  |
| PGSC0003DMG400018114 | -1.34 | down | ATFRO8/FRO8 |  |
| PGSC0003DMG400018565 | 1.95 | up | Alcohol dehydrogenase |  |
| PGSC0003DMG400018713 | 1.82 | up | Carbonyl reductase |  |
| PGSC0003DMG400018744 | 1.04 | up | Cytochrome P450-dependent fatty acid hydroxylase |  |
| PGSC0003DMG400018778 | -1.25 | down | 12-oxophytodienoate reductase 1 |  |
| PGSC0003DMG400018914 | -3.63 | down | Polyphenol oxidase |  |
| PGSC0003DMG400019185 | -4.44 | down | Diphenol oxidase |  |
| PGSC0003DMG400019186 | -2.63 | down | Ethylene-forming-enzyme-like dioxygenase |  |
| PGSC0003DMG400019718 | -1.52 | down | Amine oxidase |  |
| PGSC0003DMG400020252 | -2.13 | down | Peroxidase 55 |  |
| PGSC0003DMG400020345 | -3.40 | down | Diphenol oxidase |  |
| PGSC0003DMG400020355 | -2.37 | down | Conserved gene of unknown function |  |
| PGSC0003DMG400020618 | -1.48 | down | Tropinone reductase homolog |  |
| PGSC0003DMG400020799 | -2.37 | down | Cationic peroxidase 1 |  |
| PGSC0003DMG400021933 | 1.08 | up | Conserved gene of unknown function |  |
| PGSC0003DMG400022341 | -1.04 | down | Suberization-associated anionic peroxidase 2 |  |
| PGSC0003DMG400022392 | -4.05 | down | Reticuline oxidase |  |
| PGSC0003DMG400022430 | -2.50 | down | Polyphenoloxidase |  |
| PGSC0003DMG400022541 | -2.99 | down | Peroxidase 72 |  |
| PGSC0003DMG400022792 | 1.46 | up | Cytochrome B561 |  |
| PGSC0003DMG400022868 | -1.96 | down | 2-oxoglutarate-dependent dioxygenase |  |
| PGSC0003DMG400023193 | -1.29 | down | Short chain alcohol dehydrogenase |  |
| PGSC0003DMG400024003 | 4.62 | up | FAD binding domain containing protein |  |
| PGSC0003DMG400024643 | 1.41 | up | Flavonoid 3'-monooxygenase |  |
| PGSC0003DMG400024967 | -1.57 | down | Peroxidase |  |
| PGSC0003DMG400025254 | -1.33 | down | S-adenosyl-L-methionine-dependent uroporphyrinogen III methyltransferase |  |
| PGSC0003DMG400026080 | -1.92 | down | Cytochrome P450 |  |
| PGSC0003DMG400026276 | -2.46 | down | Leucoanthocyanidin dioxygenase |  |
| PGSC0003DMG400026575 | -3.60 | down | Class III peroxidase |  |
| PGSC0003DMG400027577 | 2.41 | up | Superoxide dismutase |  |
| PGSC0003DMG400027681 | -2.28 | down | Dopamine beta-monooxygenase |  |
| PGSC0003DMG400028221 | 1.40 | up | Tropinone reductase I |  |
| PGSC0003DMG400028244 | -1.52 | down | Short chain alcohol dehydrogenase |  |
| PGSC0003DMG400028518 | -1.83 | down | 2-oxoglutarate-dependent dioxygenase |  |
| PGSC0003DMG400029195 | 1.21 | up | Ferritin |  |
| PGSC0003DMG400029325 | -2.41 | down | PS60 protein |  |
| PGSC0003DMG400029562 | -1.74 | down | Cytochrome P450 |  |
| PGSC0003DMG400029575 | -1.15 | down | Catechol oxidase B, chloroplastic |  |
| PGSC0003DMG400030376 | -3.00 | down | Laccase |  |
| PGSC0003DMG400030382 | -5.51 | down | Class III peroxidase |  |
| PGSC0003DMG400030413 | -3.47 | down | Cytochrome P450 |  |
| PGSC0003DMG400030419 | -3.13 | down | Conserved gene of unknown function |  |
| PGSC0003DMG400030551 | -1.52 | down | Spore coat protein |  |
| PGSC0003DMG400030998 | 1.04 | up | Alcohol dehydrogenase |  |
| PGSC0003DMG400031420 | -1.15 | down | Cytokinin oxidase/dehydrogenase |  |
| PGSC0003DMG400031479 | 2.54 | up | Cinnamyl alcohol dehydrogenase |  |
| PGSC0003DMG400031519 | -1.89 | down | Conserved gene of unknown function |  |
| PGSC0003DMG400031809 | 1.17 | up | Lipoxygenase |  |
| PGSC0003DMG400032121 | -2.04 | down | Short chain alcohol dehydrogenase |  |
| PGSC0003DMG400032510 | -1.75 | down | L-ascorbate oxidase |  |
| PGSC0003DMG400033099 | -1.17 | down | Short chain dehydrogenase |  |
| PGSC0003DMG400033636 | -2.12 | down | Cytochrome P450 |  |
| PGSC0003DMG400035878 | -7.77 | down | Fatty acid desaturase |  |
| PGSC0003DMG400044958 | 1.32 | up | Conserved gene of unknown function |  |
| PGSC0003DMG401000287 | -1.54 | down | Myo-inositol oxygenase |  |
| PGSC0003DMG401001731 | 1.91 | up | Ascorbate peroxidase |  |
| PGSC0003DMG401002721 | 1.32 | up | Peroxiredoxin |  |
| PGSC0003DMG401009494 | 5.41 | up | Ferric-chelate reductase |  |
| PGSC0003DMG401018777 | -1.47 | down | 12-oxophytodienoate reductase 1 |  |
| PGSC0003DMG401019771 | -1.76 | down | Conserved gene of unknown function |  |
| PGSC0003DMG401026767 | 1.04 | up | Delta 1-pyrroline-5-carboxylate synthetase |  |
| PGSC0003DMG401029332 | -2.60 | down | Peroxidase |  |
| PGSC0003DMG402000594 | 1.31 | up | Flavonol synthase/flavanone 3-hydroxylase |  |
| PGSC0003DMG402005074 | -1.02 | down | Adenylyl-sulfate reductase |  |
| PGSC0003DMG402015497 | -3.05 | down | Pericarp peroxidase 3 |  |
| PGSC0003DMG402027116 | -2.95 | down | Laccase 90d |  |
