# Supplementary material for: Comparison of defense responses of transgenic potato lines expressing three different Rpi genes to specific Phytophthora infestans races based on transcriptome profiling
Source: PeerJ. 2020 May 5;8:e9096. doi: 10.7717/peerj.9096 (PMC7207217; doi:10.7717/peerj.9096)
Supplement: Table S7 [file peerj-08-9096-s007.docx]

**Table S7. Differential expressed genes enriched in the common biological process of response to stress (GO:0006950) for transgenic *R3a* and *R3b* lines under CN152 infection.**

| **Gene ID** | **Log2FC** | **Regulated** | **Gene annotation** | **Transgenic lines** |
| --- | --- | --- | --- | --- |
| PGSC0003DMG400000066 | 1.05 | up | Ethylene-responsive late embryogenesis | TR3a |
| PGSC0003DMG400000417 | -2.49 | down | Superoxide dismutase |  |
| PGSC0003DMG400000505 | -2.10 | down | Alpha-DOX1 |  |
| PGSC0003DMG400000523 | -1.08 | down | Kinesin light chain |  |
| PGSC0003DMG400001774 | -1.88 | down | Peroxidase |  |
| PGSC0003DMG400002161 | 1.87 | up | Conserved gene of unknown function |  |
| PGSC0003DMG400002552 | -1.17 | down | Delta TIP |  |
| PGSC0003DMG400002890 | 1.51 | up | Xenotropic and polytropic murine leukemia virus receptor ids-4 |  |
| PGSC0003DMG400002920 | -2.07 | down | RGC1 |  |
| PGSC0003DMG400002987 | 1.11 | up | DNAJ protein |  |
| PGSC0003DMG400003056 | -1.65 | down | Ethylene-responsive proteinase inhibitor 1 |  |
| PGSC0003DMG400003530 | 3.96 | up | Abscisic acid and environmental stress-inducible protein TAS14 |  |
| PGSC0003DMG400003748 | -1.86 | down | Peroxidase |  |
| PGSC0003DMG400004579 | 1.22 | up | NBS-coding resistance gene analog |  |
| PGSC0003DMG400005216 | 2.07 | up | Galactinol synthase |  |
| PGSC0003DMG400005279 | -1.22 | down | Peroxidase |  |
| PGSC0003DMG400005390 | -3.26 | down | Calmodulin-binding protein |  |
| PGSC0003DMG400005649 | -1.99 | down | Calmodulin-binding protein |  |
| PGSC0003DMG400006386 | -2.09 | down | Peroxidase |  |
| PGSC0003DMG400006533 | -5.69 | down | Rpi protein |  |
| PGSC0003DMG400006663 | -2.17 | down | ASR3 |  |
| PGSC0003DMG400006672 | 1.30 | up | Sucrose synthase |  |
| PGSC0003DMG400007514 | -5.65 | down | Glycolate oxidase |  |
| PGSC0003DMG400007993 | -1.52 | down | Pheromone receptor |  |
| PGSC0003DMG400008794 | 1.69 | up | Major latex |  |
| PGSC0003DMG400009255 | 1.93 | up | Small heat-shock protein homolog protein |  |
| PGSC0003DMG400009672 | 1.08 | up | Pom14 protein |  |
| PGSC0003DMG400010660 | -1.39 | down | Superoxide dismutase |  |
| PGSC0003DMG400010819 | 1.51 | up | 3-ketoacyl-CoA synthase 10 |  |
| PGSC0003DMG400010870 | -1.78 | down | NAC domain protein |  |
| PGSC0003DMG400011640 | -1.32 | down | Peroxidase |  |
| PGSC0003DMG400012111 | 1.21 | up | Granule-bound starch synthase 1, chloroplastic/amyloplastic |  |
| PGSC0003DMG400012503 | 2.31 | up | DNA mismatch repair protein mutS |  |
| PGSC0003DMG400012589 | -2.42 | down | Cationic peroxidase |  |
| PGSC0003DMG400013143 | 1.23 | up | Ripening induced protein |  |
| PGSC0003DMG400013486 | -3.90 | down | Disease resistance protein |  |
| PGSC0003DMG400013736 | -1.80 | down | BED finger-nbs-lrr resistance protein |  |
| PGSC0003DMG400014509 | 1.24 | up | BURP domain-containing protein |  |
| PGSC0003DMG400014558 | -1.72 | down | Conserved gene of unknown function |  |
| PGSC0003DMG400014867 | -1.52 | down | Peroxidase |  |
| PGSC0003DMG400015106 | -2.88 | down | Cell wall peroxidase |  |
| PGSC0003DMG400015228 | -1.19 | down | Peptide methionine sulfoxide reductase |  |
| PGSC0003DMG400015850 | 1.87 | up | IDS4 |  |
| PGSC0003DMG400016270 | 1.45 | up | Heat stress transcription factor A-6b |  |
| PGSC0003DMG400016730 | -1.08 | down | Sucrose synthase |  |
| PGSC0003DMG400017334 | -1.20 | down | Heat shock factor protein |  |
| PGSC0003DMG400018271 | -1.07 | down | Calmodulin binding protein |  |
| PGSC0003DMG400019446 | 1.05 | up | Annexin 11 |  |
| PGSC0003DMG400019824 | -1.15 | down | JA-induced WRKY protein |  |
| PGSC0003DMG400020252 | -2.35 | down | Peroxidase 55 |  |
| PGSC0003DMG400020433 | -1.44 | down | DNAJ heat shock N-terminal domain-containing protein |  |
| PGSC0003DMG400020799 | -2.77 | down | Cationic peroxidase 1 |  |
| PGSC0003DMG400021726 | 1.63 | up | Furin |  |
| PGSC0003DMG400021986 | -4.81 | down | Late blight resistance protein Rpi-blb2 |  |
| PGSC0003DMG400022541 | -1.56 | down | Peroxidase 72 |  |
| PGSC0003DMG400023235 | -1.03 | down | Sn-2 protein |  |
| PGSC0003DMG400023435 | -1.52 | down | Major allergen Pru ar |  |
| PGSC0003DMG400023622 | 1.12 | up | Peroxisomal small heat shock protein |  |
| PGSC0003DMG400023751 | -1.74 | down | Basic blue copper protein |  |
| PGSC0003DMG400024237 | -1.22 | down | Conserved gene of unknown function |  |
| PGSC0003DMG400024285 | -1.58 | down | Peroxidase 44 |  |
| PGSC0003DMG400024477 | -3.67 | down | Calmodulin binding protein |  |
| PGSC0003DMG400024644 | 1.78 | up | 101 kDa heat shock protein |  |
| PGSC0003DMG400024707 | 1.57 | up | Luminal binding protein |  |
| PGSC0003DMG400024785 | -1.35 | down | Calmodulin-binding protein |  |
| PGSC0003DMG400024967 | -1.00 | down | Peroxidase |  |
| PGSC0003DMG400025259 | -6.57 | down | NBS-LRR type disease resistance protein |  |
| PGSC0003DMG400026017 | 4.57 | up | Xenotropic and polytropic murine leukemia virus receptor |  |
| PGSC0003DMG400026346 | -1.40 | down | F-box family protein |  |
| PGSC0003DMG400026575 | -2.43 | down | Class III peroxidase |  |
| PGSC0003DMG400026666 | -1.25 | down | Cc-nbs-lrr resistance protein |  |
| PGSC0003DMG400027459 | -1.09 | down | ATP binding protein |  |
| PGSC0003DMG400027614 | -4.66 | down | Cell wall peroxidase |  |
| PGSC0003DMG400027728 | -1.65 | down | Conserved gene of unknown function |  |
| PGSC0003DMG400028339 | -1.31 | down | Late blight resistance protein |  |
| PGSC0003DMG400028426 | -1.39 | down | Cellulose synthase catalytic subunit |  |
| PGSC0003DMG400028624 | 3.68 | up | Small heat-shock protein |  |
| PGSC0003DMG400029195 | 1.04 | up | Ferritin |  |
| PGSC0003DMG400029313 | -1.65 | down | R2 protein |  |
| PGSC0003DMG400029341 | -1.57 | down | TSI-1 protein |  |
| PGSC0003DMG400029405 | -1.13 | down | Disease resistance protein RPM1 |  |
| PGSC0003DMG400030089 | 1.35 | up | Heat shock protein 70kD |  |
| PGSC0003DMG400030134 | -1.32 | down | MLO1 |  |
| PGSC0003DMG400030255 | 3.65 | up | Sn-1 protein |  |
| PGSC0003DMG400030339 | 2.05 | up | 17.6 kD class I small heat shock protein |  |
| PGSC0003DMG400030419 | -3.81 | down | Conserved gene of unknown function |  |
| PGSC0003DMG400030919 | 2.04 | up | Dicyanin |  |
| PGSC0003DMG400031371 | -1.06 | down | Nucleic acid binding protein |  |
| PGSC0003DMG400032793 | 1.95 | up | Heat stress transcription factor HSFA9 |  |
| PGSC0003DMG400037159 | 4.99 | up | Leucine-rich repeat containing protein |  |
| PGSC0003DMG401001731 | 1.53 | up | Ascorbate peroxidase |  |
| PGSC0003DMG401007628 | -1.48 | down | Polyribonucleotide nucleotidyltransferase |  |
| PGSC0003DMG401029332 | -2.44 | down | Peroxidase |  |
| PGSC0003DMG401030920 | -2.23 | down | Cucumber peeling cupredoxin |  |
| PGSC0003DMG401033888 | 1.56 | up | Universal stress protein family protein |  |
| PGSC0003DMG402015497 | -1.79 | down | Pericarp peroxidase 3 |  |
| PGSC0003DMG400000079 | 1.20 | up | BCL-2-associated athanogene 6 | TR3b |
| PGSC0003DMG400000417 | -3.14 | down | Superoxide dismutase |  |
| PGSC0003DMG400000505 | -4.68 | down | Alpha-DOX1 |  |
| PGSC0003DMG400000523 | -1.26 | down | Kinesin light chain |  |
| PGSC0003DMG400001598 | 2.30 | up | Snakin-2 |  |
| PGSC0003DMG400001774 | -2.32 | down | Peroxidase |  |
| PGSC0003DMG400002161 | 1.73 | up | Conserved gene of unknown function |  |
| PGSC0003DMG400002552 | -1.91 | down | Delta TIP |  |
| PGSC0003DMG400002890 | 2.77 | up | Xenotropic and polytropic murine leukemia virus receptor ids-4 |  |
| PGSC0003DMG400002920 | -1.78 | down | RGC1 |  |
| PGSC0003DMG400002987 | 1.81 | up | DNAJ protein |  |
| PGSC0003DMG400003056 | -3.47 | down | Ethylene-responsive proteinase inhibitor 1 |  |
| PGSC0003DMG400003530 | 5.25 | up | Abscisic acid and environmental stress-inducible protein TAS14 |  |
| PGSC0003DMG400003531 | 1.63 | up | Dhn1 protein |  |
| PGSC0003DMG400003645 | -1.38 | down | Ascorbate peroxidase |  |
| PGSC0003DMG400003654 | -1.37 | down | Peroxidase |  |
| PGSC0003DMG400003748 | -1.44 | down | Peroxidase |  |
| PGSC0003DMG400003822 | -1.36 | down | Cellulose synthase |  |
| PGSC0003DMG400004064 | -1.21 | down | Subtilisin inhibitor 1 |  |
| PGSC0003DMG400004211 | 2.26 | up | Photosystem Q(B) protein |  |
| PGSC0003DMG400005216 | 2.73 | up | Galactinol synthase |  |
| PGSC0003DMG400005279 | -1.27 | down | Peroxidase |  |
| PGSC0003DMG400005390 | -2.43 | down | Calmodulin-binding protein |  |
| PGSC0003DMG400005573 | 1.61 | up | Heat shock protein 83 |  |
| PGSC0003DMG400005649 | -1.28 | down | Calmodulin-binding protein |  |
| PGSC0003DMG400006386 | -1.90 | down | Peroxidase |  |
| PGSC0003DMG400006662 | 1.08 | up | Ci21A protein |  |
| PGSC0003DMG400006663 | -1.84 | down | ASR3 |  |
| PGSC0003DMG400006672 | 1.58 | up | Sucrose synthase |  |
| PGSC0003DMG400007514 | -3.53 | down | Glycolate oxidase |  |
| PGSC0003DMG400007848 | -1.62 | down | Phospholipase D |  |
| PGSC0003DMG400007993 | -1.80 | down | Pheromone receptor |  |
| PGSC0003DMG400008223 | 1.02 | up | Heat shock factor protein HSF30 |  |
| PGSC0003DMG400008794 | 2.37 | up | Major latex |  |
| PGSC0003DMG400009255 | 3.53 | up | Small heat-shock protein homolog protein |  |
| PGSC0003DMG400009509 | 1.24 | up | Heat shock protein 83 |  |
| PGSC0003DMG400009672 | 1.61 | up | Pom14 protein |  |
| PGSC0003DMG400009817 | -1.24 | down | Major latex |  |
| PGSC0003DMG400009883 | -1.15 | down | Calcium-dependent protein kinase |  |
| PGSC0003DMG400010612 | 1.43 | up | HJTR2GH1 protein |  |
| PGSC0003DMG400010660 | -1.91 | down | Superoxide dismutase |  |
| PGSC0003DMG400010819 | 1.32 | up | 3-ketoacyl-CoA synthase 10 |  |
| PGSC0003DMG400011633 | -1.05 | down | WRKY-type transcription factor |  |
| PGSC0003DMG400011640 | -1.89 | down | Peroxidase |  |
| PGSC0003DMG400012089 | 3.37 | up | Type I serine protease inhibitor |  |
| PGSC0003DMG400012111 | 1.74 | up | Granule-bound starch synthase 1, chloroplastic/amyloplastic |  |
| PGSC0003DMG400012306 | 1.04 | up | Ubiquitin carrier protein |  |
| PGSC0003DMG400012589 | -3.31 | down | Cationic peroxidase |  |
| PGSC0003DMG400013010 | 1.27 | up | 24K germin |  |
| PGSC0003DMG400013143 | 1.72 | up | Ripening induced protein |  |
| PGSC0003DMG400013692 | 1.17 | up | Conserved gene of unknown function |  |
| PGSC0003DMG400013736 | -1.18 | down | BED finger-nbs-lrr resistance protein |  |
| PGSC0003DMG400014509 | 1.34 | up | BURP domain-containing protein |  |
| PGSC0003DMG400014867 | -1.68 | down | Peroxidase |  |
| PGSC0003DMG400015106 | -4.70 | down | Cell wall peroxidase |  |
| PGSC0003DMG400015130 | 1.04 | up | Defensin P322 |  |
| PGSC0003DMG400015219 | -1.22 | down | Miraculin |  |
| PGSC0003DMG400015228 | -1.02 | down | Peptide methionine sulfoxide reductase |  |
| PGSC0003DMG400015318 | 1.01 | up | Metallothionein |  |
| PGSC0003DMG400015548 | -2.33 | down | Peroxidase 15 |  |
| PGSC0003DMG400015850 | 2.88 | up | IDS4 |  |
| PGSC0003DMG400016066 | 2.43 | up | Major latex |  |
| PGSC0003DMG400016730 | -1.58 | down | Sucrose synthase |  |
| PGSC0003DMG400017334 | -1.35 | down | Heat shock factor protein |  |
| PGSC0003DMG400018271 | -2.13 | down | Calmodulin binding protein |  |
| PGSC0003DMG400018975 | -1.02 | down | Calmodulin binding protein |  |
| PGSC0003DMG400019068 | 3.49 | up | Sn-1 protein |  |
| PGSC0003DMG400019087 | -4.93 | down | REX1 DNA Repair family protein |  |
| PGSC0003DMG400019435 | -1.06 | down | Wound-induced protein WIN2 |  |
| PGSC0003DMG400019446 | 1.01 | up | Annexin 11 |  |
| PGSC0003DMG400019824 | -1.61 | down | JA-induced WRKY protein |  |
| PGSC0003DMG400020433 | -1.41 | down | DNAJ heat shock N-terminal domain-containing protein |  |
| PGSC0003DMG400020605 | 2.25 | up | MLO1 |  |
| PGSC0003DMG400020799 | -3.08 | down | Cationic peroxidase 1 |  |
| PGSC0003DMG400021382 | -3.66 | down | Conserved gene of unknown function |  |
| PGSC0003DMG400021726 | 2.19 | up | Furin |  |
| PGSC0003DMG400022341 | -1.02 | down | Suberization-associated anionic peroxidase 2 |  |
| PGSC0003DMG400022399 | 1.82 | up | MYB transcription factor |  |
| PGSC0003DMG400022502 | -1.13 | down | Deoxyuridine 5'-triphosphate nucleotidohydrolase |  |
| PGSC0003DMG400022541 | -1.30 | down | Peroxidase 72 |  |
| PGSC0003DMG400023400 | 1.05 | up | DnaJ |  |
| PGSC0003DMG400023435 | -3.26 | down | Major allergen Pru ar |  |
| PGSC0003DMG400023622 | 1.73 | up | Peroxisomal small heat shock protein |  |
| PGSC0003DMG400023751 | -2.58 | down | Basic blue copper protein |  |
| PGSC0003DMG400024237 | -2.14 | down | Conserved gene of unknown function |  |
| PGSC0003DMG400024309 | -1.35 | down | Conserved gene of unknown function |  |
| PGSC0003DMG400024477 | -2.77 | down | Calmodulin binding protein |  |
| PGSC0003DMG400024644 | 2.71 | up | 101 kDa heat shock protein |  |
| PGSC0003DMG400024707 | 2.66 | up | Luminal binding protein |  |
| PGSC0003DMG400024849 | 1.80 | up | Alcohol dehydrogenase 1 |  |
| PGSC0003DMG400024967 | -1.10 | down | Peroxidase |  |
| PGSC0003DMG400025084 | -2.25 | down | Peroxidase 4 |  |
| PGSC0003DMG400025259 | -2.14 | down | NBS-LRR type disease resistance protein |  |
| PGSC0003DMG400025896 | 1.33 | up | Proteinase inhibitor 1 |  |
| PGSC0003DMG400026017 | 6.46 | up | Xenotropic and polytropic murine leukemia virus receptor |  |
| PGSC0003DMG400026346 | -1.64 | down | F-box family protein |  |
| PGSC0003DMG400026575 | -3.29 | down | Class III peroxidase |  |
| PGSC0003DMG400027194 | 1.10 | up | Serine/threonine-protein kinase Nek8 |  |
| PGSC0003DMG400027283 | -2.00 | down | AT-HSFB3 (Arabidopsis thaliana heat shock transcription factor B3) |  |
| PGSC0003DMG400027614 | -4.45 | down | Cell wall peroxidase |  |
| PGSC0003DMG400027728 | -2.73 | down | Conserved gene of unknown function |  |
| PGSC0003DMG400028426 | -2.12 | down | Cellulose synthase catalytic subunit |  |
| PGSC0003DMG400028624 | 4.69 | up | Small heat-shock protein |  |
| PGSC0003DMG400028634 | 1.00 | up | Heat shock protein |  |
| PGSC0003DMG400029195 | 1.16 | up | Ferritin |  |
| PGSC0003DMG400029341 | -1.98 | down | TSI-1 protein |  |
| PGSC0003DMG400029675 | -1.11 | down | Cell cycle switch 52B |  |
| PGSC0003DMG400029985 | -3.98 | down | EMB2744 |  |
| PGSC0003DMG400030058 | -1.06 | down | MAP kinase |  |
| PGSC0003DMG400030089 | 2.23 | up | Heat shock protein 70kD |  |
| PGSC0003DMG400030134 | -1.87 | down | MLO1 |  |
| PGSC0003DMG400030243 | 5.14 | up | Major latex |  |
| PGSC0003DMG400030255 | 3.69 | up | Sn-1 protein |  |
| PGSC0003DMG400030339 | 2.57 | up | 17.6 kD class I small heat shock protein |  |
| PGSC0003DMG400030359 | -1.37 | down | Pyridoxine biosynthesis protein isoform A |  |
| PGSC0003DMG400030419 | -4.05 | down | Conserved gene of unknown function |  |
| PGSC0003DMG400030430 | -1.06 | down | Anionic peroxidase swpa7 |  |
| PGSC0003DMG400030919 | 1.84 | up | Dicyanin |  |
| PGSC0003DMG400031827 | 1.58 | up | Conserved gene of unknown function |  |
| PGSC0003DMG400032549 | -5.13 | down | 8.4 kDa sulfur-rich protein |  |
| PGSC0003DMG400032793 | 2.21 | up | Heat stress transcription factor HSFA9 |  |
| PGSC0003DMG400037159 | 5.00 | up | Leucine-rich repeat containing protein |  |
| PGSC0003DMG400042669 | 3.81 | up | Sn-1 protein |  |
| PGSC0003DMG401001731 | 1.97 | up | Ascorbate peroxidase |  |
| PGSC0003DMG401013782 | -1.24 | down | Transcription factor |  |
| PGSC0003DMG401028907 | 1.44 | up | Heat shock protein 83 |  |
| PGSC0003DMG401029332 | -1.83 | down | Peroxidase |  |
| PGSC0003DMG401030920 | -2.69 | down | Cucumber peeling cupredoxin |  |
| PGSC0003DMG401033888 | 1.64 | up | Universal stress protein family protein |  |
| PGSC0003DMG402015497 | -1.64 | down | Pericarp peroxidase 3 |  |
| PGSC0003DMG403007838 | -1.02 | down | Purple acid phosphatase 3 |  |
| PGSC0003DMG403020240 | 1.88 | up | Glycerophosphodiester phosphodiesterase |  |
