# Supplementary material for: Comparison of defense responses of transgenic potato lines expressing three different Rpi genes to specific Phytophthora infestans races based on transcriptome profiling
Source: PeerJ. 2020 May 5;8:e9096. doi: 10.7717/peerj.9096 (PMC7207217; doi:10.7717/peerj.9096)
Supplement: Table S8 [file peerj-08-9096-s008.docx]

**Table S8. The down-regulated differential expressed genes enriched in the biological process of response to stress (GO:0006950) for transgenic *R1*, *R3a*, and *R3b* lines under 89148 infection**.

| **Gene ID** | **Log2FC** | **Regulated** | **Gene annotation** | **Transgenic lines** |
| --- | --- | --- | --- | --- |
| PGSC0003DMG400000417 | -1.21 | down | Superoxide dismutase | TR1 |
| PGSC0003DMG400000505 | -3.74 | down | Alpha-DOX1 |  |
| PGSC0003DMG400000523 | -1.02 | down | Kinesin light chain |  |
| PGSC0003DMG400001774 | -1.98 | down | Peroxidase |  |
| PGSC0003DMG400002275 | -1.55 | down | Cc-nbs-lrr resistance protein |  |
| PGSC0003DMG400002552 | -1.64 | down | Delta TIP |  |
| PGSC0003DMG400002920 | -1.40 | down | RGC1 |  |
| PGSC0003DMG400003056 | -2.68 | down | Ethylene-responsive proteinase inhibitor 1 |  |
| PGSC0003DMG400003574 | -1.13 | down | Ccaat-binding transcription factor |  |
| PGSC0003DMG400003748 | -2.65 | down | Peroxidase |  |
| PGSC0003DMG400003822 | -1.20 | down | Cellulose synthase |  |
| PGSC0003DMG400004561 | -1.50 | down | Late blight resistance protein Rpi-blb2 |  |
| PGSC0003DMG400005279 | -1.48 | down | Peroxidase |  |
| PGSC0003DMG400005351 | -1.09 | down | MLO1 |  |
| PGSC0003DMG400006145 | -1.15 | down | Histidine kinase 1, 2, 3 plant |  |
| PGSC0003DMG400006386 | -2.79 | down | Peroxidase |  |
| PGSC0003DMG400006663 | -2.33 | down | ASR3 |  |
| PGSC0003DMG400008393 | -4.31 | down | Cc-nbs-lrr resistance protein |  |
| PGSC0003DMG400008595 | -1.15 | down | Cc-nbs-lrr resistance protein |  |
| PGSC0003DMG400010465 | -2.58 | down | Peroxidase |  |
| PGSC0003DMG400010660 | -1.03 | down | Superoxide dismutase |  |
| PGSC0003DMG400010870 | -1.41 | down | NAC domain protein |  |
| PGSC0003DMG400011640 | -1.72 | down | Peroxidase |  |
| PGSC0003DMG400012163 | -1.21 | down | Ubiquitin ligase protein cop1 |  |
| PGSC0003DMG400012443 | -1.06 | down | Phytosulfokine receptor |  |
| PGSC0003DMG400012471 | -1.61 | down | Conserved gene of unknown function |  |
| PGSC0003DMG400012589 | -3.41 | down | Cationic peroxidase |  |
| PGSC0003DMG400012974 | -1.03 | down | Conserved gene of unknown function |  |
| PGSC0003DMG400013486 | -7.12 | down | Disease resistance protein |  |
| PGSC0003DMG400013721 | -4.99 | down | Plant disease resistant protein |  |
| PGSC0003DMG400013736 | -2.48 | down | BED finger-nbs-lrr resistance protein |  |
| PGSC0003DMG400014047 | -1.15 | down | Disease resistance protein R3a |  |
| PGSC0003DMG400014867 | -2.11 | down | Peroxidase |  |
| PGSC0003DMG400015693 | -1.93 | down | Resistance gene |  |
| PGSC0003DMG400016730 | -1.16 | down | Sucrose synthase |  |
| PGSC0003DMG400017439 | -1.01 | down | Alpha DNA polymerase |  |
| PGSC0003DMG400018462 | -1.07 | down | Disease resistance protein |  |
| PGSC0003DMG400018570 | -1.64 | down | Disease resistance protein R3a |  |
| PGSC0003DMG400018903 | -3.50 | down | Cc-nbs-lrr resistance protein |  |
| PGSC0003DMG400019418 | -1.14 | down | Cc-nbs-lrr resistance protein |  |
| PGSC0003DMG400019435 | -1.35 | down | Wound-induced protein WIN2 |  |
| PGSC0003DMG400019627 | -1.09 | down | Disease resistance protein Gpa2 |  |
| PGSC0003DMG400019803 | -1.18 | down | NBS-LRR resistance protein |  |
| PGSC0003DMG400020433 | -2.23 | down | DNAJ heat shock N-terminal domain-containing protein |  |
| PGSC0003DMG400021382 | -3.12 | down | Conserved gene of unknown function |  |
| PGSC0003DMG400021986 | -4.71 | down | Late blight resistance protein Rpi-blb2 |  |
| PGSC0003DMG400022541 | -2.61 | down | Peroxidase 72 |  |
| PGSC0003DMG400023751 | -1.11 | down | Basic blue copper protein |  |
| PGSC0003DMG400024477 | -2.92 | down | Calmodulin binding protein |  |
| PGSC0003DMG400024967 | -1.25 | down | Peroxidase |  |
| PGSC0003DMG400026346 | -1.87 | down | F-box family protein |  |
| PGSC0003DMG400026428 | -1.22 | down | Sucrose-phosphate synthase isoform B |  |
| PGSC0003DMG400026433 | -1.29 | down | ATP binding protein |  |
| PGSC0003DMG400026575 | -2.34 | down | Class III peroxidase |  |
| PGSC0003DMG400028308 | -1.27 | down | LIGULELESS1 protein |  |
| PGSC0003DMG400028426 | -1.47 | down | Cellulose synthase catalytic subunit |  |
| PGSC0003DMG400029457 | -1.16 | down | Hero resistance protein |  |
| PGSC0003DMG400030134 | -1.18 | down | MLO1 |  |
| PGSC0003DMG400030267 | -3.27 | down | Bacterial spot disease resistance protein 4 |  |
| PGSC0003DMG400030382 | -5.51 | down | Class III peroxidase |  |
| PGSC0003DMG400030419 | -2.19 | down | Conserved gene of unknown function |  |
| PGSC0003DMG400030927 | -1.21 | down | Hemoglobin |  |
| PGSC0003DMG400031318 | -1.86 | down | Tir-nbs-lrr resistance protein |  |
| PGSC0003DMG401007575 | -1.11 | down | Cc-nbs-lrr resistance protein |  |
| PGSC0003DMG401008349 | -2.19 | down | Cc-nbs-lrr resistance protein |  |
| PGSC0003DMG401009819 | -1.34 | down | Resistance protein PSH-RGH6 |  |
| PGSC0003DMG401010943 | -1.36 | down | Nbs-lrr resistance protein |  |
| PGSC0003DMG401016933 | -1.40 | down | Disease resistance protein; Calcium-binding EF-hand; AAA ATPase |  |
| PGSC0003DMG401027402 | -1.99 | down | Disease resistance protein R3a |  |
| PGSC0003DMG401029332 | -2.47 | down | Peroxidase |  |
| PGSC0003DMG401030920 | -2.30 | down | Cucumber peeling cupredoxin |  |
| PGSC0003DMG402015497 | -2.16 | down | Pericarp peroxidase 3 |  |
| PGSC0003DMG400000417 | -2.05 | down | Superoxide dismutase | TR3a |
| PGSC0003DMG400000505 | -2.32 | down | Alpha-DOX1 |  |
| PGSC0003DMG400001774 | -1.69 | down | Peroxidase |  |
| PGSC0003DMG400002275 | -1.04 | down | Cc-nbs-lrr resistance protein |  |
| PGSC0003DMG400002552 | -1.54 | down | Delta TIP |  |
| PGSC0003DMG400003056 | -1.39 | down | Ethylene-responsive proteinase inhibitor 1 |  |
| PGSC0003DMG400003574 | -1.40 | down | Ccaat-binding transcription factor |  |
| PGSC0003DMG400003654 | -1.06 | down | Peroxidase |  |
| PGSC0003DMG400003748 | -3.26 | down | Peroxidase |  |
| PGSC0003DMG400003822 | -1.30 | down | Cellulose synthase |  |
| PGSC0003DMG400005273 | -1.02 | down | Peroxidase |  |
| PGSC0003DMG400005279 | -2.06 | down | Peroxidase |  |
| PGSC0003DMG400005390 | -1.13 | down | Calmodulin-binding protein |  |
| PGSC0003DMG400005649 | -1.06 | down | Calmodulin-binding protein |  |
| PGSC0003DMG400006386 | -3.07 | down | Peroxidase |  |
| PGSC0003DMG400006533 | -2.32 | down | Rpi protein |  |
| PGSC0003DMG400006570 | -1.00 | down | Tospovirus resistance protein C |  |
| PGSC0003DMG400006663 | -2.15 | down | ASR3 |  |
| PGSC0003DMG400007848 | -1.48 | down | Phospholipase D |  |
| PGSC0003DMG400007993 | -1.23 | down | Pheromone receptor |  |
| PGSC0003DMG400008393 | -4.50 | down | Cc-nbs-lrr resistance protein |  |
| PGSC0003DMG400009883 | -1.02 | down | Calcium-dependent protein kinase |  |
| PGSC0003DMG400010660 | -1.61 | down | Superoxide dismutase |  |
| PGSC0003DMG400010870 | -1.79 | down | NAC domain protein |  |
| PGSC0003DMG400011640 | -1.93 | down | Peroxidase |  |
| PGSC0003DMG400012471 | -1.78 | down | Conserved gene of unknown function |  |
| PGSC0003DMG400012589 | -2.82 | down | Cationic peroxidase |  |
| PGSC0003DMG400012974 | -1.02 | down | Conserved gene of unknown function |  |
| PGSC0003DMG400013486 | -7.32 | down | Disease resistance protein |  |
| PGSC0003DMG400013736 | -2.13 | down | BED finger-nbs-lrr resistance protein |  |
| PGSC0003DMG400014867 | -2.28 | down | Peroxidase |  |
| PGSC0003DMG400015693 | -1.19 | down | Resistance gene |  |
| PGSC0003DMG400016730 | -1.49 | down | Sucrose synthase |  |
| PGSC0003DMG400017334 | -1.09 | down | Heat shock factor protein |  |
| PGSC0003DMG400018271 | -1.05 | down | Calmodulin binding protein |  |
| PGSC0003DMG400019418 | -1.09 | down | Cc-nbs-lrr resistance protein |  |
| PGSC0003DMG400019435 | -1.36 | down | Wound-induced protein WIN2 |  |
| PGSC0003DMG400020252 | -2.01 | down | Peroxidase 55 |  |
| PGSC0003DMG400020433 | -1.48 | down | DNAJ heat shock N-terminal domain-containing protein |  |
| PGSC0003DMG400020799 | -2.40 | down | Cationic peroxidase 1 |  |
| PGSC0003DMG400022541 | -2.53 | down | Peroxidase 72 |  |
| PGSC0003DMG400023751 | -1.81 | down | Basic blue copper protein |  |
| PGSC0003DMG400024477 | -3.91 | down | Calmodulin binding protein |  |
| PGSC0003DMG400024754 | -1.58 | down | Respiratory burst oxidase homolog protein B |  |
| PGSC0003DMG400024967 | -1.35 | down | Peroxidase |  |
| PGSC0003DMG400026346 | -2.06 | down | F-box family protein |  |
| PGSC0003DMG400026433 | -1.11 | down | ATP binding protein |  |
| PGSC0003DMG400026575 | -3.02 | down | Class III peroxidase |  |
| PGSC0003DMG400028426 | -1.61 | down | Cellulose synthase catalytic subunit |  |
| PGSC0003DMG400029457 | -1.48 | down | Hero resistance protein |  |
| PGSC0003DMG400029724 | -1.03 | down | PHO2 |  |
| PGSC0003DMG400030134 | -1.24 | down | MLO1 |  |
| PGSC0003DMG400030382 | -1.77 | down | Class III peroxidase |  |
| PGSC0003DMG400030419 | -5.25 | down | Conserved gene of unknown function |  |
| PGSC0003DMG400030927 | -1.48 | down | Hemoglobin |  |
| PGSC0003DMG401007628 | -1.22 | down | Polyribonucleotide nucleotidyltransferase |  |
| PGSC0003DMG401015877 | -1.32 | down | NBS-LRR protein |  |
| PGSC0003DMG401027402 | -1.07 | down | Disease resistance protein R3a |  |
| PGSC0003DMG401029332 | -3.40 | down | Peroxidase |  |
| PGSC0003DMG401030920 | -2.81 | down | Cucumber peeling cupredoxin |  |
| PGSC0003DMG402015497 | -3.64 | down | Pericarp peroxidase 3 |  |
| PGSC0003DMG400000417 | -2.86 | down | Superoxide dismutase | TR3b |
| PGSC0003DMG400000505 | -5.07 | down | Alpha-DOX1 |  |
| PGSC0003DMG400000511 | -1.10 | down | Class III peroxidase |  |
| PGSC0003DMG400001066 | -1.05 | down | Potassium channel |  |
| PGSC0003DMG400001223 | -3.39 | down | NAC domain protein |  |
| PGSC0003DMG400001774 | -2.79 | down | Peroxidase |  |
| PGSC0003DMG400002552 | -1.84 | down | Delta TIP |  |
| PGSC0003DMG400002920 | -1.53 | down | RGC1 |  |
| PGSC0003DMG400003056 | -4.46 | down | Ethylene-responsive proteinase inhibitor 1 |  |
| PGSC0003DMG400003574 | -1.19 | down | Ccaat-binding transcription factor |  |
| PGSC0003DMG400003654 | -1.03 | down | Peroxidase |  |
| PGSC0003DMG400003748 | -2.53 | down | Peroxidase |  |
| PGSC0003DMG400003822 | -1.23 | down | Cellulose synthase |  |
| PGSC0003DMG400004064 | -2.34 | down | Subtilisin inhibitor 1 |  |
| PGSC0003DMG400005062 | -1.42 | down | Peroxidase |  |
| PGSC0003DMG400005273 | -1.43 | down | Peroxidase |  |
| PGSC0003DMG400005279 | -1.67 | down | Peroxidase |  |
| PGSC0003DMG400005390 | -1.86 | down | Calmodulin-binding protein |  |
| PGSC0003DMG400005649 | -1.09 | down | Calmodulin-binding protein |  |
| PGSC0003DMG400006386 | -2.82 | down | Peroxidase |  |
| PGSC0003DMG400006663 | -2.53 | down | ASR3 |  |
| PGSC0003DMG400006855 | -1.02 | down | S-adenosyl-methionine-sterol-C-methyltransferase |  |
| PGSC0003DMG400008588 | -1.09 | down | Disease resistance protein RGA4 |  |
| PGSC0003DMG400009817 | -1.97 | down | Major latex |  |
| PGSC0003DMG400010465 | -4.52 | down | Peroxidase |  |
| PGSC0003DMG400010660 | -2.02 | down | Superoxide dismutase |  |
| PGSC0003DMG400010870 | -1.37 | down | NAC domain protein |  |
| PGSC0003DMG400011640 | -2.10 | down | Peroxidase |  |
| PGSC0003DMG400011974 | -1.01 | down | BZIP domain class transcription factor |  |
| PGSC0003DMG400012589 | -3.37 | down | Cationic peroxidase |  |
| PGSC0003DMG400013486 | -2.37 | down | Disease resistance protein |  |
| PGSC0003DMG400013721 | -5.00 | down | Plant disease resistant protein |  |
| PGSC0003DMG400014055 | -1.62 | down | Peroxidase |  |
| PGSC0003DMG400014867 | -1.99 | down | Peroxidase |  |
| PGSC0003DMG400015219 | -1.83 | down | Miraculin |  |
| PGSC0003DMG400016278 | -1.11 | down | Mannosyl-oligosaccharide 1,2-alpha-mannosidase IA |  |
| PGSC0003DMG400016460 | -1.21 | down | 15.4 kDa class V heat shock protein |  |
| PGSC0003DMG400016730 | -1.98 | down | Sucrose synthase |  |
| PGSC0003DMG400017334 | -1.18 | down | Heat shock factor protein |  |
| PGSC0003DMG400017439 | -1.07 | down | Alpha DNA polymerase |  |
| PGSC0003DMG400018271 | -1.45 | down | Calmodulin binding protein |  |
| PGSC0003DMG400018441 | -1.19 | down | Late blight resistance protein |  |
| PGSC0003DMG400018975 | -1.35 | down | Calmodulin binding protein |  |
| PGSC0003DMG400019087 | -3.85 | down | REX1 DNA Repair family protein |  |
| PGSC0003DMG400019435 | -1.11 | down | Wound-induced protein WIN2 |  |
| PGSC0003DMG400020252 | -2.13 | down | Peroxidase 55 |  |
| PGSC0003DMG400020433 | -1.02 | down | DNAJ heat shock N-terminal domain-containing protein |  |
| PGSC0003DMG400020799 | -2.37 | down | Cationic peroxidase 1 |  |
| PGSC0003DMG400021986 | -4.72 | down | Late blight resistance protein Rpi-blb2 |  |
| PGSC0003DMG400022341 | -1.04 | down | Suberization-associated anionic peroxidase 2 |  |
| PGSC0003DMG400022541 | -2.99 | down | Peroxidase 72 |  |
| PGSC0003DMG400023326 | -1.26 | down | ATDGK2 |  |
| PGSC0003DMG400023751 | -2.55 | down | Basic blue copper protein |  |
| PGSC0003DMG400024237 | -1.39 | down | Conserved gene of unknown function |  |
| PGSC0003DMG400024309 | -1.40 | down | Conserved gene of unknown function |  |
| PGSC0003DMG400024477 | -3.13 | down | Calmodulin binding protein |  |
| PGSC0003DMG400024967 | -1.57 | down | Peroxidase |  |
| PGSC0003DMG400026346 | -1.76 | down | F-box family protein |  |
| PGSC0003DMG400026575 | -3.60 | down | Class III peroxidase |  |
| PGSC0003DMG400027283 | -2.82 | down | AT-HSFB3 (Arabidopsis thaliana heat shock transcription factor B3) |  |
| PGSC0003DMG400027728 | -2.01 | down | Conserved gene of unknown function |  |
| PGSC0003DMG400028426 | -1.56 | down | Cellulose synthase catalytic subunit |  |
| PGSC0003DMG400029542 | -1.45 | down | Sensor histidine kinase |  |
| PGSC0003DMG400030134 | -1.66 | down | MLO1 |  |
| PGSC0003DMG400030359 | -1.05 | down | Pyridoxine biosynthesis protein isoform A |  |
| PGSC0003DMG400030382 | -5.51 | down | Class III peroxidase |  |
| PGSC0003DMG400030419 | -3.13 | down | Conserved gene of unknown function |  |
| PGSC0003DMG400030927 | -1.13 | down | Hemoglobin |  |
| PGSC0003DMG401008349 | -1.05 | down | Cc-nbs-lrr resistance protein |  |
| PGSC0003DMG401013782 | -1.04 | down | Transcription factor |  |
| PGSC0003DMG401029332 | -2.60 | down | Peroxidase |  |
| PGSC0003DMG401030920 | -2.77 | down | Cucumber peeling cupredoxin |  |
| PGSC0003DMG402015497 | -3.05 | down | Pericarp peroxidase 3 |  |
| PGSC0003DMG403007838 | -1.41 | down | Purple acid phosphatase 3 |  |
