# Supplementary material for: Comparison of defense responses of transgenic potato lines expressing three different Rpi genes to specific Phytophthora infestans races based on transcriptome profiling
Source: PeerJ. 2020 May 5;8:e9096. doi: 10.7717/peerj.9096 (PMC7207217; doi:10.7717/peerj.9096)
Supplement: Table S9 [file peerj-08-9096-s009.docx]

**Table S9. The down-regulated differential expressed genes enriched in the biological process of oxidation reduction (GO:0055114) for transgenic *R1*, *R3a*, and *R3b* lines under CN152 infection**.

| **Gene ID** | **Log2FC** | **Regulated** | **Gene annotation** | **Transgenic lines** |
| --- | --- | --- | --- | --- |
| PGSC0003DMG400000148 | -2.81 | down | Cytochrome P450 | TR1 |
| PGSC0003DMG400000277 | -1.99 | down | Cytokinin oxidase/dehydrogenase 2 |  |
| PGSC0003DMG400000417 | -1.50 | down | Superoxide dismutase |  |
| PGSC0003DMG400000505 | -3.63 | down | Alpha-DOX1 |  |
| PGSC0003DMG400000582 | -1.07 | down | 1-aminocyclopropane-1-carboxylate oxidase |  |
| PGSC0003DMG400001774 | -2.18 | down | Peroxidase |  |
| PGSC0003DMG400003340 | -1.07 | down | P450 mono-oxygenase |  |
| PGSC0003DMG400003748 | -1.95 | down | Peroxidase |  |
| PGSC0003DMG400003754 | -1.57 | down | Cytokinin oxidase/dehydrogenase |  |
| PGSC0003DMG400004311 | -1.58 | down | 9-cis-epoxycarotenoid dioxygenase |  |
| PGSC0003DMG400004822 | -2.49 | down | Oxidoreductase |  |
| PGSC0003DMG400005284 | -1.18 | down | 3-dehydroquinate dehydratase / shikimate dehydrogenase isoform 2 |  |
| PGSC0003DMG400005498 | -2.49 | down | Multifunctional protein |  |
| PGSC0003DMG400005515 | -1.77 | down | Ascorbate oxidase |  |
| PGSC0003DMG400006386 | -1.97 | down | Peroxidase |  |
| PGSC0003DMG400006764 | -1.79 | down | Cytokinin oxidase/dehydrogenase |  |
| PGSC0003DMG400007179 | -3.37 | down | P-coumaroyl quinate/shikimate 3'-hydroxylase |  |
| PGSC0003DMG400007180 | -1.95 | down | P-coumaroyl quinate/shikimate 3'-hydroxylase |  |
| PGSC0003DMG400007514 | -4.10 | down | Glycolate oxidase |  |
| PGSC0003DMG400007613 | -1.31 | down | Alternative oxidase |  |
| PGSC0003DMG400007639 | -1.95 | down | Cytochrome P450 |  |
| PGSC0003DMG400008267 | -1.74 | down | CYP72A54 |  |
| PGSC0003DMG400008389 | -1.43 | down | Short chain alcohol dehydrogenase |  |
| PGSC0003DMG400008826 | -1.58 | down | Laccase |  |
| PGSC0003DMG400008898 | -1.87 | down | Laccase |  |
| PGSC0003DMG400008947 | -1.28 | down | Desacetoxyvindoline 4-hydroxylase |  |
| PGSC0003DMG400009621 | -1.41 | down | Cytochrome P450 |  |
| PGSC0003DMG400009623 | -1.42 | down | Cytochrome P450 71D7 |  |
| PGSC0003DMG400009759 | -1.30 | down | Cytochrome P450 71D7 |  |
| PGSC0003DMG400010859 | -3.09 | down | Lipoxygenase |  |
| PGSC0003DMG400011429 | -2.48 | down | Conserved gene of unknown function |  |
| PGSC0003DMG400011640 | -1.02 | down | Peroxidase |  |
| PGSC0003DMG400012589 | -2.95 | down | Cationic peroxidase |  |
| PGSC0003DMG400013696 | -2.71 | down | Cytochrome P450 |  |
| PGSC0003DMG400014013 | -1.39 | down | Flavonoid 3-hydroxylase |  |
| PGSC0003DMG400014095 | -1.57 | down | 2,4-dienoyl-CoA reductase |  |
| PGSC0003DMG400014168 | -1.12 | down | Respiratory burst oxidase homolog protein C |  |
| PGSC0003DMG400014272 | -1.70 | down | (S)-N-methylcoclaurine 3'-hydroxylase isozyme |  |
| PGSC0003DMG400014442 | -2.19 | down | Flavonoid 3-hydroxylase |  |
| PGSC0003DMG400014867 | -1.47 | down | Peroxidase |  |
| PGSC0003DMG400015061 | -1.33 | down | Cytochrome P450 |  |
| PGSC0003DMG400015106 | -2.57 | down | Cell wall peroxidase |  |
| PGSC0003DMG400015228 | -1.31 | down | Peptide methionine sulfoxide reductase |  |
| PGSC0003DMG400015355 | -1.65 | down | Monooxygenase |  |
| PGSC0003DMG400015484 | -1.23 | down | Laccase |  |
| PGSC0003DMG400016043 | -4.33 | down | Cytochrome P450 |  |
| PGSC0003DMG400016623 | -1.65 | down | Cytochrome P450 |  |
| PGSC0003DMG400016778 | -1.08 | down | Cytochrome P450 |  |
| PGSC0003DMG400018131 | -1.03 | down | CYP71AU1 |  |
| PGSC0003DMG400018133 | -1.14 | down | Cytochrome P450 71A4 |  |
| PGSC0003DMG400018778 | -1.11 | down | 12-oxophytodienoate reductase 1 |  |
| PGSC0003DMG400018914 | -4.35 | down | Polyphenol oxidase |  |
| PGSC0003DMG400019185 | -1.60 | down | Diphenol oxidase |  |
| PGSC0003DMG400019718 | -1.05 | down | Amine oxidase |  |
| PGSC0003DMG400019872 | -1.02 | down | Adenylyl-sulfate reductase |  |
| PGSC0003DMG400020252 | -1.74 | down | Peroxidase 55 |  |
| PGSC0003DMG400020334 | -1.75 | down | Prephenate dehydrogenase |  |
| PGSC0003DMG400020345 | -2.06 | down | Diphenol oxidase |  |
| PGSC0003DMG400020618 | -1.79 | down | Tropinone reductase homolog |  |
| PGSC0003DMG400020799 | -2.76 | down | Cationic peroxidase 1 |  |
| PGSC0003DMG400020809 | -1.99 | down | Cytochrome P450 |  |
| PGSC0003DMG400021382 | -4.67 | down | Conserved gene of unknown function |  |
| PGSC0003DMG400022025 | -2.17 | down | Flavin monooxygenase |  |
| PGSC0003DMG400022075 | -1.30 | down | Cytochrome P450 |  |
| PGSC0003DMG400022341 | -1.04 | down | Suberization-associated anionic peroxidase 2 |  |
| PGSC0003DMG400022430 | -1.41 | down | Polyphenoloxidase |  |
| PGSC0003DMG400022541 | -1.10 | down | Peroxidase 72 |  |
| PGSC0003DMG400022892 | -1.02 | down | Elicitor-inducible cytochrome P450 |  |
| PGSC0003DMG400025333 | -1.05 | down | Short chain alcohol dehydrogenase |  |
| PGSC0003DMG400025795 | -1.61 | down | Cytochrome P450 |  |
| PGSC0003DMG400025924 | -1.11 | down | 2-oxoglutarate-dependent dioxygenase |  |
| PGSC0003DMG400026276 | -1.26 | down | Leucoanthocyanidin dioxygenase |  |
| PGSC0003DMG400026575 | -1.89 | down | Class III peroxidase |  |
| PGSC0003DMG400027333 | -1.60 | down | Leucoanthocyanidin dioxygenase |  |
| PGSC0003DMG400027631 | -1.32 | down | Gibberellin 2-oxidase |  |
| PGSC0003DMG400028175 | -1.40 | down | Cytochrome P450 76A2 |  |
| PGSC0003DMG400030413 | -2.45 | down | Cytochrome P450 |  |
| PGSC0003DMG400030419 | -2.83 | down | Conserved gene of unknown function |  |
| PGSC0003DMG400031515 | -1.99 | down | Conserved gene of unknown function |  |
| PGSC0003DMG400031519 | -1.45 | down | Conserved gene of unknown function |  |
| PGSC0003DMG400032121 | -1.96 | down | Short chain alcohol dehydrogenase |  |
| PGSC0003DMG400032157 | -4.06 | down | Oxidoreductase, 2OG-Fe(II) oxygenase family protein |  |
| PGSC0003DMG400033099 | -1.13 | down | Short chain dehydrogenase |  |
| PGSC0003DMG400033642 | -1.09 | down | 2,4-dienoyl-CoA reductase |  |
| PGSC0003DMG400033932 | -1.74 | down | Cytochrome P450 hydroxylase |  |
| PGSC0003DMG400035878 | -1.31 | down | Fatty acid desaturase |  |
| PGSC0003DMG400039221 | -1.08 | down | Desacetoxyvindoline 4-hydroxylase |  |
| PGSC0003DMG400044730 | -1.68 | down | Acyl- |  |
| PGSC0003DMG401000287 | -2.61 | down | Myo-inositol oxygenase |  |
| PGSC0003DMG401029332 | -1.43 | down | Peroxidase |  |
| PGSC0003DMG402000506 | -2.77 | down | Alpha-DOX2 |  |
| PGSC0003DMG402015497 | -2.09 | down | Pericarp peroxidase 3 |  |
| PGSC0003DMG400000148 | -2.23 | down | Cytochrome P450 | TR3a |
| PGSC0003DMG400000277 | -2.22 | down | Cytokinin oxidase/dehydrogenase 2 |  |
| PGSC0003DMG400000417 | -2.49 | down | Superoxide dismutase |  |
| PGSC0003DMG400000505 | -2.10 | down | Alpha-DOX1 |  |
| PGSC0003DMG400000582 | -1.33 | down | 1-aminocyclopropane-1-carboxylate oxidase |  |
| PGSC0003DMG400000798 | -1.05 | down | Cytochrome P450 |  |
| PGSC0003DMG400001774 | -1.88 | down | Peroxidase |  |
| PGSC0003DMG400002340 | -1.02 | down | Elicitor-inducible cytochrome P450 |  |
| PGSC0003DMG400003512 | -1.81 | down | Laccase |  |
| PGSC0003DMG400003748 | -1.86 | down | Peroxidase |  |
| PGSC0003DMG400004311 | -1.96 | down | 9-cis-epoxycarotenoid dioxygenase |  |
| PGSC0003DMG400004822 | -1.87 | down | Oxidoreductase |  |
| PGSC0003DMG400004824 | -1.17 | down | CYP92B3 |  |
| PGSC0003DMG400005279 | -1.22 | down | Peroxidase |  |
| PGSC0003DMG400005284 | -1.07 | down | 3-dehydroquinate dehydratase / shikimate dehydrogenase isoform 2 |  |
| PGSC0003DMG400005498 | -1.74 | down | Multifunctional protein |  |
| PGSC0003DMG400005515 | -1.96 | down | Ascorbate oxidase |  |
| PGSC0003DMG400005698 | -1.13 | down | Gibberellin 3-oxidase |  |
| PGSC0003DMG400006386 | -2.09 | down | Peroxidase |  |
| PGSC0003DMG400006764 | -1.36 | down | Cytokinin oxidase/dehydrogenase |  |
| PGSC0003DMG400007180 | -1.84 | down | P-coumaroyl quinate/shikimate 3'-hydroxylase |  |
| PGSC0003DMG400007514 | -5.65 | down | Glycolate oxidase |  |
| PGSC0003DMG400007639 | -1.16 | down | Cytochrome P450 |  |
| PGSC0003DMG400008267 | -1.76 | down | CYP72A54 |  |
| PGSC0003DMG400008389 | -1.20 | down | Short chain alcohol dehydrogenase |  |
| PGSC0003DMG400008826 | -1.94 | down | Laccase |  |
| PGSC0003DMG400008898 | -1.68 | down | Laccase |  |
| PGSC0003DMG400008947 | -1.06 | down | Desacetoxyvindoline 4-hydroxylase |  |
| PGSC0003DMG400009621 | -1.22 | down | Cytochrome P450 |  |
| PGSC0003DMG400009623 | -1.28 | down | Cytochrome P450 71D7 |  |
| PGSC0003DMG400009759 | -1.39 | down | Cytochrome P450 71D7 |  |
| PGSC0003DMG400010021 | -1.27 | down | Short-chain dehydrogenase |  |
| PGSC0003DMG400010660 | -1.39 | down | Superoxide dismutase |  |
| PGSC0003DMG400010859 | -2.31 | down | Lipoxygenase |  |
| PGSC0003DMG400011019 | -2.30 | down | Diphenol oxidase |  |
| PGSC0003DMG400011429 | -1.95 | down | Conserved gene of unknown function |  |
| PGSC0003DMG400011640 | -1.32 | down | Peroxidase |  |
| PGSC0003DMG400012589 | -2.42 | down | Cationic peroxidase |  |
| PGSC0003DMG400013352 | -1.15 | down | Dopamine beta-monooxygenase |  |
| PGSC0003DMG400013696 | -1.49 | down | Cytochrome P450 |  |
| PGSC0003DMG400014013 | -1.07 | down | Flavonoid 3-hydroxylase |  |
| PGSC0003DMG400014095 | -1.31 | down | 2,4-dienoyl-CoA reductase |  |
| PGSC0003DMG400014272 | -1.33 | down | (S)-N-methylcoclaurine 3'-hydroxylase isozyme |  |
| PGSC0003DMG400014442 | -1.80 | down | Flavonoid 3-hydroxylase |  |
| PGSC0003DMG400014867 | -1.52 | down | Peroxidase |  |
| PGSC0003DMG400015106 | -2.88 | down | Cell wall peroxidase |  |
| PGSC0003DMG400015228 | -1.19 | down | Peptide methionine sulfoxide reductase |  |
| PGSC0003DMG400015350 | -3.83 | down | Monooxygenase |  |
| PGSC0003DMG400015484 | -2.58 | down | Laccase |  |
| PGSC0003DMG400016623 | -1.17 | down | Cytochrome P450 |  |
| PGSC0003DMG400018114 | -1.26 | down | ATFRO8/FRO8 |  |
| PGSC0003DMG400018131 | -1.15 | down | CYP71AU1 |  |
| PGSC0003DMG400018778 | -1.23 | down | 12-oxophytodienoate reductase 1 |  |
| PGSC0003DMG400018914 | -3.91 | down | Polyphenol oxidase |  |
| PGSC0003DMG400019185 | -2.87 | down | Diphenol oxidase |  |
| PGSC0003DMG400020252 | -2.35 | down | Peroxidase 55 |  |
| PGSC0003DMG400020334 | -2.62 | down | Prephenate dehydrogenase |  |
| PGSC0003DMG400020345 | -3.09 | down | Diphenol oxidase |  |
| PGSC0003DMG400020355 | -2.00 | down | Conserved gene of unknown function |  |
| PGSC0003DMG400020618 | -1.76 | down | Tropinone reductase homolog |  |
| PGSC0003DMG400020799 | -2.77 | down | Cationic peroxidase 1 |  |
| PGSC0003DMG400020809 | -1.02 | down | Cytochrome P450 |  |
| PGSC0003DMG400021107 | -2.96 | down | Conserved gene of unknown function |  |
| PGSC0003DMG400021152 | -1.20 | down | Alcohol dehydrogenase |  |
| PGSC0003DMG400022025 | -2.45 | down | Flavin monooxygenase |  |
| PGSC0003DMG400022430 | -2.37 | down | Polyphenoloxidase |  |
| PGSC0003DMG400022541 | -1.56 | down | Peroxidase 72 |  |
| PGSC0003DMG400022892 | -1.49 | down | Elicitor-inducible cytochrome P450 |  |
| PGSC0003DMG400023193 | -1.32 | down | Short chain alcohol dehydrogenase |  |
| PGSC0003DMG400024285 | -1.58 | down | Peroxidase 44 |  |
| PGSC0003DMG400024967 | -1.00 | down | Peroxidase |  |
| PGSC0003DMG400025795 | -1.47 | down | Cytochrome P450 |  |
| PGSC0003DMG400026276 | -1.16 | down | Leucoanthocyanidin dioxygenase |  |
| PGSC0003DMG400026575 | -2.43 | down | Class III peroxidase |  |
| PGSC0003DMG400027333 | -1.53 | down | Leucoanthocyanidin dioxygenase |  |
| PGSC0003DMG400027614 | -4.66 | down | Cell wall peroxidase |  |
| PGSC0003DMG400027631 | -1.68 | down | Gibberellin 2-oxidase |  |
| PGSC0003DMG400027632 | -1.59 | down | Gibberellin 2-oxidase 2 |  |
| PGSC0003DMG400029330 | -3.22 | down | Monooxygenase |  |
| PGSC0003DMG400029575 | -1.02 | down | Catechol oxidase B, chloroplastic |  |
| PGSC0003DMG400030376 | -1.82 | down | Laccase |  |
| PGSC0003DMG400030413 | -2.07 | down | Cytochrome P450 |  |
| PGSC0003DMG400030419 | -3.81 | down | Conserved gene of unknown function |  |
| PGSC0003DMG400032121 | -1.36 | down | Short chain alcohol dehydrogenase |  |
| PGSC0003DMG400032155 | -1.00 | down | Lipoxygenase |  |
| PGSC0003DMG400032510 | -1.62 | down | L-ascorbate oxidase |  |
| PGSC0003DMG400033636 | -1.68 | down | Cytochrome P450 |  |
| PGSC0003DMG400033932 | -1.45 | down | Cytochrome P450 hydroxylase |  |
| PGSC0003DMG400035878 | -1.68 | down | Fatty acid desaturase |  |
| PGSC0003DMG401000287 | -2.19 | down | Myo-inositol oxygenase |  |
| PGSC0003DMG401011339 | -1.35 | down | NAD(P)H-quinone oxidoreductase subunit I, chloroplastic |  |
| PGSC0003DMG401018777 | -1.39 | down | 12-oxophytodienoate reductase 1 |  |
| PGSC0003DMG401029332 | -2.44 | down | Peroxidase |  |
| PGSC0003DMG402015497 | -1.79 | down | Pericarp peroxidase 3 |  |
| PGSC0003DMG402027116 | -2.84 | down | Laccase 90d |  |
| PGSC0003DMG400000022 | -4.17 | down | (+)-neomenthol dehydrogenase | TR3b |
| PGSC0003DMG400000277 | -2.36 | down | Cytokinin oxidase/dehydrogenase 2 |  |
| PGSC0003DMG400000417 | -3.14 | down | Superoxide dismutase |  |
| PGSC0003DMG400000505 | -4.68 | down | Alpha-DOX1 |  |
| PGSC0003DMG400000798 | -1.15 | down | Cytochrome P450 |  |
| PGSC0003DMG400001249 | -1.17 | down | Gibberellin 20 oxidase |  |
| PGSC0003DMG400001774 | -2.32 | down | Peroxidase |  |
| PGSC0003DMG400001932 | -1.10 | down | 6-phosphogluconate dehydrogenase, decarboxylating |  |
| PGSC0003DMG400003512 | -2.46 | down | Laccase |  |
| PGSC0003DMG400003645 | -1.38 | down | Ascorbate peroxidase |  |
| PGSC0003DMG400003654 | -1.37 | down | Peroxidase |  |
| PGSC0003DMG400003748 | -1.44 | down | Peroxidase |  |
| PGSC0003DMG400003754 | -1.15 | down | Cytokinin oxidase/dehydrogenase |  |
| PGSC0003DMG400004800 | -1.17 | down | Gene of unknown function |  |
| PGSC0003DMG400004822 | -2.09 | down | Oxidoreductase |  |
| PGSC0003DMG400004844 | -2.23 | down | Glucose-methanol-choline (Gmc) oxidoreductase |  |
| PGSC0003DMG400005279 | -1.27 | down | Peroxidase |  |
| PGSC0003DMG400005284 | -1.18 | down | 3-dehydroquinate dehydratase / shikimate dehydrogenase isoform 2 |  |
| PGSC0003DMG400005498 | -2.13 | down | Multifunctional protein |  |
| PGSC0003DMG400005515 | -1.77 | down | Ascorbate oxidase |  |
| PGSC0003DMG400005698 | -1.51 | down | Gibberellin 3-oxidase |  |
| PGSC0003DMG400006159 | -1.46 | down | Dopamine beta-monooxygenase |  |
| PGSC0003DMG400006386 | -1.90 | down | Peroxidase |  |
| PGSC0003DMG400006692 | -1.42 | down | Cytochrome P450 hydroxylase |  |
| PGSC0003DMG400006764 | -1.93 | down | Cytokinin oxidase/dehydrogenase |  |
| PGSC0003DMG400007180 | -1.98 | down | P-coumaroyl quinate/shikimate 3'-hydroxylase |  |
| PGSC0003DMG400007514 | -3.53 | down | Glycolate oxidase |  |
| PGSC0003DMG400007639 | -2.07 | down | Cytochrome P450 |  |
| PGSC0003DMG400008267 | -1.15 | down | CYP72A54 |  |
| PGSC0003DMG400008356 | -1.04 | down | NADH-glutamate dehydrogenase |  |
| PGSC0003DMG400008389 | -1.38 | down | Short chain alcohol dehydrogenase |  |
| PGSC0003DMG400008826 | -3.16 | down | Laccase |  |
| PGSC0003DMG400008898 | -3.10 | down | Laccase |  |
| PGSC0003DMG400008947 | -1.09 | down | Desacetoxyvindoline 4-hydroxylase |  |
| PGSC0003DMG400009621 | -1.44 | down | Cytochrome P450 |  |
| PGSC0003DMG400009623 | -1.16 | down | Cytochrome P450 71D7 |  |
| PGSC0003DMG400009759 | -1.53 | down | Cytochrome P450 71D7 |  |
| PGSC0003DMG400010660 | -1.91 | down | Superoxide dismutase |  |
| PGSC0003DMG400010859 | -2.89 | down | Lipoxygenase |  |
| PGSC0003DMG400011019 | -1.97 | down | Diphenol oxidase |  |
| PGSC0003DMG400011098 | -3.17 | down | Oxidoreductase |  |
| PGSC0003DMG400011429 | -2.79 | down | Conserved gene of unknown function |  |
| PGSC0003DMG400011640 | -1.89 | down | Peroxidase |  |
| PGSC0003DMG400012589 | -3.31 | down | Cationic peroxidase |  |
| PGSC0003DMG400013696 | -2.52 | down | Cytochrome P450 |  |
| PGSC0003DMG400013879 | -1.13 | down | Quinone reductase family protein |  |
| PGSC0003DMG400014013 | -1.23 | down | Flavonoid 3-hydroxylase |  |
| PGSC0003DMG400014095 | -1.18 | down | 2,4-dienoyl-CoA reductase |  |
| PGSC0003DMG400014168 | -1.01 | down | Respiratory burst oxidase homolog protein C |  |
| PGSC0003DMG400014272 | -1.72 | down | (S)-N-methylcoclaurine 3'-hydroxylase isozyme |  |
| PGSC0003DMG400014442 | -2.29 | down | Flavonoid 3-hydroxylase |  |
| PGSC0003DMG400014867 | -1.68 | down | Peroxidase |  |
| PGSC0003DMG400015106 | -4.70 | down | Cell wall peroxidase |  |
| PGSC0003DMG400015228 | -1.02 | down | Peptide methionine sulfoxide reductase |  |
| PGSC0003DMG400015355 | -1.57 | down | Monooxygenase |  |
| PGSC0003DMG400015484 | -2.87 | down | Laccase |  |
| PGSC0003DMG400015548 | -2.33 | down | Peroxidase 15 |  |
| PGSC0003DMG400016043 | -4.17 | down | Cytochrome P450 |  |
| PGSC0003DMG400016623 | -1.75 | down | Cytochrome P450 |  |
| PGSC0003DMG400016778 | -1.20 | down | Cytochrome P450 |  |
| PGSC0003DMG400018114 | -1.38 | down | ATFRO8/FRO8 |  |
| PGSC0003DMG400018131 | -1.29 | down | CYP71AU1 |  |
| PGSC0003DMG400018135 | -1.23 | down | Cytochrome P450 71A4 |  |
| PGSC0003DMG400018778 | -1.80 | down | 12-oxophytodienoate reductase 1 |  |
| PGSC0003DMG400018914 | -4.00 | down | Polyphenol oxidase |  |
| PGSC0003DMG400019185 | -3.95 | down | Diphenol oxidase |  |
| PGSC0003DMG400019718 | -1.13 | down | Amine oxidase |  |
| PGSC0003DMG400020345 | -5.54 | down | Diphenol oxidase |  |
| PGSC0003DMG400020355 | -2.71 | down | Conserved gene of unknown function |  |
| PGSC0003DMG400020618 | -1.88 | down | Tropinone reductase homolog |  |
| PGSC0003DMG400020799 | -3.08 | down | Cationic peroxidase 1 |  |
| PGSC0003DMG400021107 | -4.12 | down | Conserved gene of unknown function |  |
| PGSC0003DMG400021382 | -3.66 | down | Conserved gene of unknown function |  |
| PGSC0003DMG400022341 | -1.02 | down | Suberization-associated anionic peroxidase 2 |  |
| PGSC0003DMG400022405 | -1.03 | down | NADH dehydrogenase |  |
| PGSC0003DMG400022430 | -3.33 | down | Polyphenoloxidase |  |
| PGSC0003DMG400022541 | -1.30 | down | Peroxidase 72 |  |
| PGSC0003DMG400023193 | -1.06 | down | Short chain alcohol dehydrogenase |  |
| PGSC0003DMG400023957 | -1.38 | down | Prephenate dehydrogenase |  |
| PGSC0003DMG400024161 | -1.18 | down | Monoxygenase |  |
| PGSC0003DMG400024693 | -1.67 | down | Lipoxygenase |  |
| PGSC0003DMG400024967 | -1.10 | down | Peroxidase |  |
| PGSC0003DMG400025084 | -2.25 | down | Peroxidase 4 |  |
| PGSC0003DMG400025795 | -1.41 | down | Cytochrome P450 |  |
| PGSC0003DMG400026080 | -1.59 | down | Cytochrome P450 |  |
| PGSC0003DMG400026276 | -2.22 | down | Leucoanthocyanidin dioxygenase |  |
| PGSC0003DMG400026575 | -3.29 | down | Class III peroxidase |  |
| PGSC0003DMG400027333 | -1.77 | down | Leucoanthocyanidin dioxygenase |  |
| PGSC0003DMG400027614 | -4.45 | down | Cell wall peroxidase |  |
| PGSC0003DMG400027631 | -1.60 | down | Gibberellin 2-oxidase |  |
| PGSC0003DMG400027632 | -2.97 | down | Gibberellin 2-oxidase 2 |  |
| PGSC0003DMG400027681 | -2.71 | down | Dopamine beta-monooxygenase |  |
| PGSC0003DMG400028887 | -1.68 | down | Lipoxygenase |  |
| PGSC0003DMG400029562 | -1.64 | down | Cytochrome P450 |  |
| PGSC0003DMG400029575 | -1.43 | down | Catechol oxidase B, chloroplastic |  |
| PGSC0003DMG400030376 | -2.31 | down | Laccase |  |
| PGSC0003DMG400030413 | -2.84 | down | Cytochrome P450 |  |
| PGSC0003DMG400030419 | -4.05 | down | Conserved gene of unknown function |  |
| PGSC0003DMG400030430 | -1.06 | down | Anionic peroxidase swpa7 |  |
| PGSC0003DMG400030551 | -1.18 | down | Spore coat protein |  |
| PGSC0003DMG400031420 | -1.05 | down | Cytokinin oxidase/dehydrogenase |  |
| PGSC0003DMG400031519 | -1.43 | down | Conserved gene of unknown function |  |
| PGSC0003DMG400031836 | -2.18 | down | Primary amine oxidase |  |
| PGSC0003DMG400032121 | -1.64 | down | Short chain alcohol dehydrogenase |  |
| PGSC0003DMG400032510 | -1.98 | down | L-ascorbate oxidase |  |
| PGSC0003DMG400033099 | -1.77 | down | Short chain dehydrogenase |  |
| PGSC0003DMG400033636 | -1.73 | down | Cytochrome P450 |  |
| PGSC0003DMG400033932 | -1.79 | down | Cytochrome P450 hydroxylase |  |
| PGSC0003DMG400035769 | -1.06 | down | Cytochrome P450 |  |
| PGSC0003DMG401000287 | -2.13 | down | Myo-inositol oxygenase |  |
| PGSC0003DMG401018777 | -2.16 | down | 12-oxophytodienoate reductase 1 |  |
| PGSC0003DMG401019771 | -1.60 | down | Conserved gene of unknown function |  |
| PGSC0003DMG401029332 | -1.83 | down | Peroxidase |  |
| PGSC0003DMG402005074 | -1.13 | down | Adenylyl-sulfate reductase |  |
| PGSC0003DMG402015497 | -1.64 | down | Pericarp peroxidase 3 |  |
| PGSC0003DMG402027116 | -5.10 | down | Laccase 90d |  |
