# Supplementary material for: Comparison of defense responses of transgenic potato lines expressing three different Rpi genes to specific Phytophthora infestans races based on transcriptome profiling
Source: PeerJ. 2020 May 5;8:e9096. doi: 10.7717/peerj.9096 (PMC7207217; doi:10.7717/peerj.9096)
Supplement: Table S10 [file peerj-08-9096-s010.docx]

**Table S10. The comparison of RNA-Seq to log2 qRT-PCR levels.**

| **Gene ID** | ***R* genes** | **89148** | |  | **CN152** | |
| --- | --- | --- | --- | --- | --- | --- |
|  |  | **Log2(FPKM_R/ FPKM WT)** | **log2(qRT-PCR)** |  | **Log2(FPKM_R/ FPKM WT)** | **log2(qRT-PCR)** |
| PGSC0003DMG400008364 | *R1* | 1.53 | 1.44 |  | 2.77 | 2.81 |
|  | *R3a* | 1.88 | 1.75 |  | 1.02 | 0.35 |
|  | *R3b* | 2.92 | 2.43 |  | 3.58 | 4.87 |
| PGSC0003DMG400000417 | *R1* | -1.21 | -1.28 |  | -1.50 | -1.51 |
|  | *R3a* | -2.05 | -2.22 |  | -2.50 | -2.62 |
|  | *R3b* | -2.87 | -3.19 |  | -3.18 | -3.05 |
| PGSC0003DMG400010128 | *R1* | 8.23 | 4.62 |  | 5.29 | 3.42 |
|  | *R3a* | 7.31 | 4.02 |  | 6.86 | 4.29 |
|  | *R3b* | 9.49 | 6.02 |  | 8.93 | 6.13 |
| PGSC0003DMG400010139 | *R1* | 3.86 | 2.10 |  | 3.69 | 2.49 |
|  | *R3a* | 1.97 | 1.53 |  | 4.52 | 3.25 |
|  | *R3b* | 5.67 | 4.23 |  | 7.17 | 5.02 |
| PGSC0003DMG400015289 | *R1* | 1.50 | 1.50 |  | 1.71 | 1.11 |
|  | *R3a* | -0.17 | -0.17 |  | 1.31 | 0.50 |
|  | *R3b* | 2.59 | 2.74 |  | 3.24 | 3.18 |
| PGSC0003DMG400011502 | *R1* | 0.41 | 0.24 |  | -0.15 | -0.03 |
|  | *R3a* | 0.59 | 0.27 |  | -0.19 | -0.28 |
|  | *R3b* | 1.58 | 1.74 |  | 1.04 | 1.00 |
| PGSC0003DMG400018407 | *R1* | 0.59 | 0.83 |  | 0.25 | 0.13 |
|  | *R3a* | 0.17 | 0.27 |  | 0.59 | 0.24 |
|  | *R3b* | -0.07 | 0.07 |  | 0.40 | -0.12 |
| PGSC0003DMG400004062 | *R1* | -0.23 | -0.47 |  | 0.29 | 0.26 |
|  | *R3a* | -0.06 | -0.18 |  | 0.02 | 0.00 |
|  | *R3b* | -1.17 | -1.56 |  | -1.01 | -1.17 |
| PGSC0003DMG400002042 | *R1* | -0.71 | -0.54 |  | 1.40 | 1.56 |
|  | *R3a* | -1.38 | -1.35 |  | 1.91 | 1.83 |
|  | *R3b* | -2.31 | -2.24 |  | 1.34 | 1.12 |
| PGSC0003DMG400004064 | *R1* | -0.55 | -0.73 |  | -0.19 | -0.27 |
|  | *R3a* | -0.61 | -0.68 |  | -0.41 | -0.79 |
|  | *R3b* | -2.10 | -2.18 |  | -1.14 | -1.43 |
| PGSC0003DMG400006226 | *R1* | -1.18 | -1.19 |  | -0.75 | -0.92 |
|  | *R3a* | -1.11 | -0.96 |  | -0.93 | -0.86 |
|  | *R3b* | -0.92 | -0.82 |  | -0.92 | -0.59 |
| PGSC0003DMG400010815 | *R1* | -1.10 | -0.91 |  | -1.52 | -1.52 |
|  | *R3a* | -1.50 | -1.25 |  | -1.26 | -0.87 |
|  | *R3b* | -1.67 | -1.31 |  | -1.91 | -2.06 |
| PGSC0003DMG400020174 | *R1* | 1.64 | 1.53 |  | 1.30 | 1.83 |
|  | *R3a* | 0.92 | 0.85 |  | 1.19 | 1.51 |
|  | *R3b* | 1.11 | 0.66 |  | 1.47 | 2.21 |
| PGSC0003DMG400023458 | *R1* | -1.03 | -1.03 |  | -0.53 | -0.44 |
|  | *R3a* | -0.80 | -0.77 |  | -1.18 | -0.54 |
|  | *R3b* | -0.98 | -1.11 |  | -1.29 | -0.63 |
| PGSC0003DMG400023619 | *R1* | 0.83 | 1.44 |  | 0.20 | 0.32 |
|  | *R3a* | 0.66 | 0.80 |  | -0.35 | 0.13 |
|  | *R3b* | 1.14 | 1.58 |  | 1.47 | 1.65 |
| PGSC0003DMG400025263 | *R1* | -0.75 | -0.69 |  | -1.12 | -1.83 |
|  | *R3a* | -0.36 | -0.46 |  | -0.63 | -0.99 |
|  | *R3b* | -0.10 | -0.13 |  | -0.73 | -1.27 |
